# Evolving Research Focus on Diet and Cardiovascular Disease: A Systematic Review of 298 Cohort Studies Published from 2019 to 2024

**DOI:** 10.3390/nu17132126

**Published:** 2025-06-26

**Authors:** Vicky Wai Ki Chan, Gebretsadkan Gebremedhin Gebretsadik, Pooja Panchal, Noya Yue Zhu, Daniel Kam Wah Mok, Kwok Tai Chui, Kenneth Ka Hei Lo

**Affiliations:** 1 Department of Food Science and Nutrition, The Hong Kong Polytechnic University, Hong Kong SAR, China; waiki-vicky.chan@connect.polyu.hk (V.W.K.C.); gebretsadkan.gebretsadik@connect.polyu.hk (G.G.G.); poojapanchal20@gmail.com (P.P.); daniel.mok@polyu.edu.hk (D.K.W.M.); 2 Department of Nutrition and Dietetics, School of Public Health, Mekelle University, Mekelle 0231, Ethiopia; 3 Nutrition and Dietetics Program, Symbiosis School of Culinary Arts and Nutritional Sciences (SSCANS), Symbiosis International (Deemed University), Pune 412115, India; 4 Jockey Club School of Public Health and Primary Care, The Chinese University of Hong Kong, Hong Kong SAR, China; 1155218902@link.cuhk.edu.hk; 5 School of Science and Technology, Hong Kong Metropolitan University, Hong Kong SAR, China; 6 Research Institute for Smart Ageing, The Hong Kong Polytechnic University, Hong Kong SAR, China

**Keywords:** cardiovascular diseases, dietary exposures, cohort studies, dietary patterns, food groups, nutrients, systematic review, trend analysis

## Abstract

Background/Objectives: Cardiovascular diseases (CVDs) are a leading cause of mortality globally. Growing studies have been conducted to examine the diet–CVD association to alleviate the health and economic burdens associated with CVDs, but beneficial dietary factors may vary by study region and cohort. There was a need to identify the trends in diet–CVD research by study region and current emerging dietary exposures of interest, which could inform areas for future research and the regions where evidence is relatively limited. Methods: A comprehensive search of multiple databases was performed to identify eligible prospective cohorts examining diet–CVD associations published between 2019 and 2024. Trends in dietary exposure, including dietary patterns, food groups, and nutrients, were analyzed by publication year and geographical distribution. Results: A total of 298 studies were included in the review. While the United States continued to lead in the number of CVD–diet cohort studies, China has significantly increased its contributions over the past five years, increasing from 2.1% to 14.3%. The cohorts that contributed the most to the literature included the Nurses’ Health Study and the Danish Diet, Cancer, and Health cohort. Although food groups accounted for the highest number with respect to dietary exposure overall, there was a notable shift in diet–CVD cohort studies from a focus on nutrients to dietary patterns. Plant-based and Mediterranean diets were the most frequently investigated, while ultra-processed foods and country-specific dietary indices also gained prominence. Conclusions: This systematic review highlighted the shift towards dietary patterns in nutritional epidemiology, emphasizing the importance of understanding the role of nutrition in health through holistic dietary approaches. The observed trends in dietary exposure research suggested the need for future studies to delve deeper into the complexities of dietary patterns, including how cultural and socioeconomic elements defined the nuances of country-specific dietary patterns.

## 1. Introduction

Cardiovascular diseases (CVDs) are a leading cause of mortality globally [1]. A recent projection study suggested that the prevalence of CVDs was nearly double, increasing from 598 million in 2025 to 1.14 billion by 2050, which corresponds to an annual growth rate of 3.6% [2]. In 2019, around 17.9 million people died from CVDs, accounting for 32% of all global deaths [3]. By 2025, it is estimated that there could be 20.5 million global CVD deaths, a number that was expected to increase significantly to 35.6 million deaths by 2050, representing a 73.4% increase [2]. The economic burden of CVDs is equally concerning as the healthcare costs associated with CVDs remain substantial worldwide. For instance, in Europe, healthcare costs related to CVDs have exceeded EUR 280 billion [4]. In the US, healthcare costs for CVD care are over USD 400 billion, which is projected to exceed USD 1344 billion per annum by 2050 [5]. These alarming trends highlight the need for effective management strategies to alleviate the health and economic burdens associated with CVDs.

Alongside factors such as lack of exercise, overweight and obesity, stress, alcohol consumption, and smoking, healthy diet is a critical determinant in CVD prevention [6]. Earlier nutritional research primarily focused on individual nutrients, such as saturated fats, trans fats, and cholesterol, and their direct associations with cardiovascular health [7,8]. However, in recent decades, the field has shifted from an emphasis on single nutrients and specific foods to a more comprehensive understanding of dietary patterns [9]. The 2021 Dietary Guidance to Improve Cardiovascular Health by the American Heart Association underscored the significance of dietary patterns beyond individual nutrients or foods, and suggested people initiate heart-healthy dietary patterns early in life, including the Mediterranean diet, the Dietary Approaches to Stop Hypertension (DASH) diet, the Healthy US-Style diet, and healthy vegetarian diets [10]. In alignment with this, the 2021 Canadian Cardiovascular Society Guidelines suggested that adopting healthy dietary patterns, such as the Mediterranean diet, Portfolio diet, DASH diet, and other plant-based dietary patterns, was beneficial with respect to CVD prevention [11]. Additionally, the 2021 European Society of Cardiology guidelines on CVD prevention recommended the Mediterranean diet and a shift from animal-based to plant-based diets, emphasizing the intake of fruits, vegetables, wholegrain cereals, and low-fat protein sources [12].

To comprehend interindividual responses to nutrients or dietary exposure over time, randomized clinical trials or feeding trials are essential, but it is often impractical to perform these studies across all age groups and with sufficient follow-up periods [13]. Instead, high-quality longitudinal cohort studies have provided a more feasible channel to investigate the temporal relationships between dietary exposures and their associated health effects, aiding the formulation of dietary guidelines [13]. For example, the World Health Organization (WHO) strongly recommended both adults and children reduce daily trans-fatty acid intake to 1% of total energy intake based on the findings of a systematic review of prospective observational studies [14]. The WHO’s existing guidance to limit free sugar intake to less than 10% of total energy intake was also supported by available findings from cohort studies [15].

However, beneficial dietary factors may vary by study region and cohort. For instance, while it has previously been observed that soy and isoflavone intake was not significantly associated with CVD mortality risks in Chinese older adults in the Singapore Chinese Health Study [16], a higher intake of isoflavones and tofu was beneficial in terms of reducing the risk of CVD among US postmenopausal women according to three prospective cohort studies [17]. These inconsistencies underscore the effect of geographic context and cohort characteristics on study outcomes, leading to concerns about the contributions of different countries and the diversity of the utilized cohorts. It was necessary to examine the current trends of diet–CVD research to reduce inconsistent findings and provide further evidence for formulating dietary recommendations. Therefore, the aim of this systematic review is to identify trends in diet–CVD research by study region and current emerging dietary exposures of interest, which could inform areas for future research and the regions where evidence is relatively limited.

## 2. Methods

The protocol for this systematic review has been registered with PROSPERO (registration number: CRD42024589050). The search strategy of this systematic review was pre-defined in a previously published protocol [18] and was conducted according to the standards of the Meta-analysis of Observational Studies in Epidemiology [19]. This work was a nested study from the review of the validation procedures of food frequency questionnaires (FFQs).

### 2.1. Search Strategy

In this systematic review, a literature search was conducted across four databases, namely PubMed, Web of Science, Embase, and Scopus. Keywords related to “cohort studies”, “cardiovascular diseases,” and “dietary assessment methods” were used to identify potential articles published between 2019 and 2024 (see Table 1). The reference lists of the retrieved articles were also reviewed to identify further eligible studies.

### 2.2. Study Selection

Cohort studies that investigated the association between dietary exposure and the incidence of CVD outcomes were included if they met the following criteria: (1) human participants of any age and sex; (2) dietary exposure, including food groups, dietary patterns, or specific nutrients, with dietary assessments conducted using FFQs or diet history questionnaires; (3) CVD-related outcomes; and (4) cohort studies published from 2019 to 2024. Other study designs (i.e., intervention studies, non-cohort observational studies, non-peer-reviewed articles, abstracts, conference proceedings, and gray literature) and studies whose full text was not in English were excluded.

Only studies that employed FFQs for dietary exposure assessment were included as this tool could effectively reflect long-term dietary habits and was more common in terms of examining the prospective relationship with CVD risk [20].

### 2.3. Data Extraction

Two reviewers (G.G.G. and V.W.K.C.) independently screened the retrieved articles. In cases of discrepancies, decisions were made through discussions and consensus. The following information was extracted from each included study: namely, first author, year of publication, study origin, cohort used, sample size, mean or median follow-up periods, age and sex of participants, dietary exposure, dietary assessment tools used, and CVD outcomes.

### 2.4. Quality Assessment

Two independent reviewers (P.P. and V.W.K.C.) evaluated the quality of included studies using Nutrition Quality Evaluation Strengthening Tools (NUQUEST). This method combined risk of bias (RoB) components from existing assessment methods with nutrition-specific criteria, detailed guidance, and worksheets that addressed RoB issues in relation to dietary exposure [21].

This assessment tool comprised four components: selection of cohorts, comparability of cohorts, outcome determination, and nutrition-specific aspects. The rating system for each component offered a broad range of response options (yes/probably yes; probably no/no), with the addition of “not applicable” for items where a response was not valid. The overall rating options for each section were as follows: good, indicating that almost all criteria were met with little to no concerns and low RoB; neutral, indicating that most criteria were met with some flaws and moderate RoB; and poor, indicating that most or all criteria were not met, with significant flaws and high RoB [21].

### 2.5. Data Analysis

To identify trends in diet–CVD research, all included studies were categorized by publication year and analyzed according to how the focus on various types of dietary exposure has evolved over time. This involved assessing annual publication counts to detect shifts in research focus regarding dietary patterns, food groups, or specific nutrients. The number and proportion of studies in these categories were examined to highlight areas that have recently gained increased attention. Additionally, the contributions of different countries and cohorts to the body of research on the impact of diet on CVDs were evaluated, tracking changes in these factors over the years.

## 3. Results

### 3.1. Study Selection

**Figure 1:**
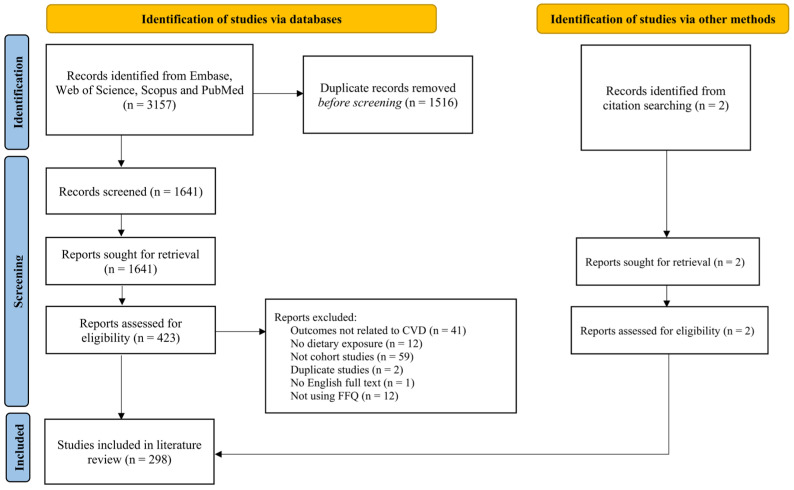
Flow diagram of the selection process used for CVD–diet cohort studies published between 2019 and 2024 included in this systematic review.

The initial database search yielded a total of 3157 articles. Following the screening of titles and abstracts, 423 papers were selected for full-text review. Additionally, two articles were identified through citation searching, resulting in a total of 298 articles included in this review. The complete flow diagram with respect to study selection is illustrated in Figure 1.

### 3.2. Characteristics of the Included Studies

**Figure 2:**
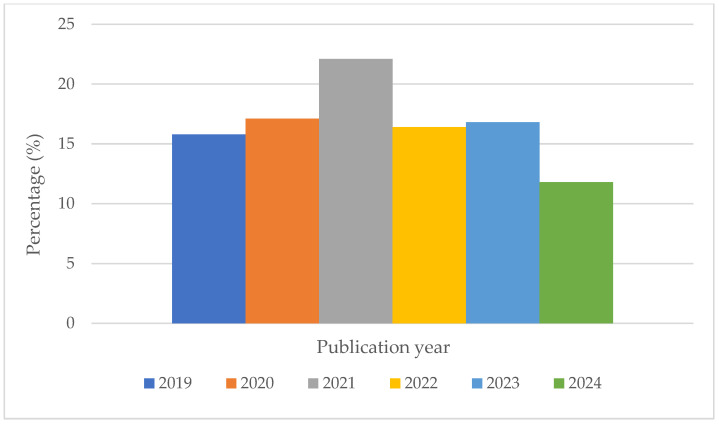
Distribution of CVD–diet cohort studies published between 2019 and 2024.

The detailed characteristics of the 298 cohort studies are summarized in Supplementary Table S1. The sample sizes of the cohorts varied significantly, ranging from 313 participants in the ATTICA study to 566,398 in the National Institutes of Health-AARP Diet and Health Study. All studies were prospective cohorts, with an average follow-up duration of 14.3 years and an average male participation rate of 45%. Notably, nearly a quarter of the studies were conducted in 2021, while the fewest studies were from 2024. Overall, the publication years were relatively evenly distributed (see Figure 2).

### 3.3. Country of Origin for Included Studies

Of the 298 articles included, 92 (30.9%) were based on cohorts from the United States, followed by Japan with 31 studies (10.4%). Iran and China each contributed 27 studies (9.1%), while Denmark was notable for its 19 studies (6.4%). Other significant contributors included South Korea with 14 studies (4.7%) and Australia with 12 studies (4.0%) (see Table 2).

### 3.4. Major Contributor Cohorts

In terms of cohort contributions to the included papers, the Nurses’ Health Study (NHS), Nurses’ Health Study II (NHSII), and Health Professionals Follow-up Study (HPFS) were the most prominent, with a combined total of 27 articles. Other notable cohorts included the Japan Public Health Center-based Prospective Study (JPHC) cohort (19 articles), the Danish Diet, Cancer, and Health cohort (16 articles), and the Tehran Lipid and Glucose Study (TLGS) (12 articles), as shown in Table 3.

### 3.5. Trends in Dietary Exposure

Of the 298 studies included in this review, 118 (39.6%) examined the association of food groups with CVD outcomes, 97 (32.6%) focused on dietary patterns, and 77 (25.8%) investigated specific nutrients (see Table 4).

**Figure 3:**
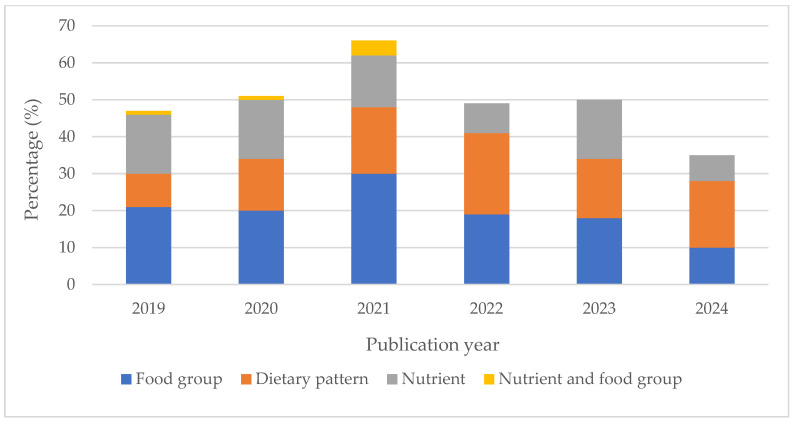
Distribution with respect to dietary exposure in CVD–diet cohort studies between 2019 and 2024.

Although food groups accounted for the highest number with respect to dietary exposure overall, there appeared to be a shift in diet–CVD cohort studies from a focus on nutrients to dietary patterns. The number of studies examining dietary patterns doubled, increasing from 9 in 2019 to 18 in 2024. In contrast, studies focused on nutrients decreased from 16 to 7 during the same period. Additionally, the number of studies analyzing food groups in terms of dietary exposure declined by 11 between 2019 and 2024 (see Figure 3).

#### 3.5.1. Dietary Patterns

Vegetarian or plant-based diets were the most frequently studied, comprising 22 studies (18.3%), followed by Mediterranean diet variations with 18 studies (15.0%) and country-specific indices at 17 studies (14.2%). The number of studies on dietary patterns increased from 16 in 2019 to 23 in 2024, indicating a growing interest in holistic dietary approaches (see Table 5).

#### 3.5.2. Food Groups

Animal-source foods were the most commonly investigated category, comprising 42 studies (30.4%), followed by fruits and vegetables with 23 studies (16.7%) and ultra-processed foods at 15 studies (10.9%). Despite being the most studied category overall, the number of studies on food groups fluctuated, peaking in 2021 with 45 studies and declining to 12 studies in 2024 (see Table 6).

#### 3.5.3. Nutrients

Lipids were the most frequently researched category, with 28 studies (27.2%), followed by minerals at 21 studies (20.4%) and phytochemicals at 17 studies (16.5%). The number of nutrient-focused studies declined from 18 in 2019 to 10 in 2024, indicating a shift away from single-nutrient analyses (see Table 7).

### 3.6. Quality Assessment

NUQUEST was utilized to evaluate the quality of the included cohorts. Out of 298 studies, there were 17 poor, 116 neutral, and 165 good studies. Among the studies rated as ‘poor’, the main risk of bias came from the ‘nutrition-specific’ domain because most of them failed to describe whether frequency and quantity of dietary exposure were accurately and reliably measured, and whether baseline dietary exposure differences between the groups was maintained over the whole study. The detailed ratings are summarized in Supplementary Table S2.

## 4. Discussion

### 4.1. Summary of Main Findings

A total of 298 articles were included in this systematic review. The studies exhibited diverse geographical representation, with a significant proportion originating from the United States, Japan, Iran, and China. The NHS, NHSII, HPFS, JPHC study, and the Danish Diet, Cancer, and Health cohorts were the major contributors to this review. Although food groups accounted for the highest number with respect to dietary exposure overall, there was a notable shift in diet–CVD cohort studies from a focus on nutrients to dietary patterns.

This review highlighted the importance of comprehensive, long-term, and well-designed studies in terms of elucidating the relationship between diet and CVD. Numerous studies utilizing these cohorts reported significant associations between diet and CVD outcomes. Research from these cohorts revealed important links between CVD outcomes and the intake of food groups such as nuts [22] and avocados [23], as well as nutrients such as vitamin D [24], proteins [25], and dietary sugar [26]. Dietary patterns, including the prudent and Western diets [27] and the planetary health diet [28], were also significantly associated with CVD outcomes. These studies demonstrated how the NHS and HPFS effectively advanced our understanding of prospective associations between diet and a range of CVD outcomes. Similarly, the JPHC study provided valuable data on traditional Japanese dietary patterns and their impact on cardiovascular health, which may prove critical in terms of informing public health recommendations and dietary guidelines. While the United States continues to lead in the number of CVD–diet cohort studies, China has significantly increased its contributions over the past five years, increasing from 2.1% to 14.3%. About 29.3% of studies from the United States primarily relied on the NHS, NHSII, and HPFS cohorts, and 8.7% of them utilized the WHI, MVP, and NIH-AARP cohorts, respectively. In contrast, studies from China employed a diverse range of cohorts, with 22.2% from the China Kadoorie Biobank Study, 11.1% from the Guangdong Biobank Study, and the remainder from other diverse cohorts.

### 4.2. The Emergence of Dietary Patterns

A notable trend observed in the studies included was a shift from focusing on individual nutrients to broader dietary patterns, particularly plant-based and Mediterranean diets, which emerged as the most frequently studied diets. For example, the Mediterranean diet, rich in plant-based foods and unsaturated fats, has been consistently linked to lower incidences of coronary heart disease and stroke [29]. Similarly, vegetarian or plant-based diets have been associated with improved cardiovascular outcomes, likely due to their high fiber and antioxidant content and lower levels of saturated fats [30]. Notably, country-specific dietary patterns (e.g., Nordic, Japanese, or Dutch diets) accounted for 14.2% of the dietary pattern studies in this analysis, reflecting a growing recognition of regional dietary habits and their relevance for public health applications. This emphasis aligned with evidence suggesting that localized dietary indices enhanced the precision of CVD risk assessments by incorporating cultural, environmental, and socioeconomic factors.

The review also highlighted a significant shift toward using diet quality indices and scores as exposure measures. The Alternative Healthy Eating Index, recently updated to align with current nutritional science, has shown stronger inverse associations with CVD risk compared to previous versions [31]. This evolution mirrored broader trends in nutritional epidemiology, where holistic tools like the Global Diet Quality Score and Planetary Health Diet Index were increasingly adopted to quantify adherence to evidence-based guidelines while integrating sustainability metrics [32]. This advancement in dietary assessment tools reflected a deeper understanding of how overall diet quality, rather than isolated components, affected cardiovascular health.

In parallel, research on specific food groups revealed distinct priorities. Animal-source foods were frequently investigated due to ongoing debates about their dual role as sources of essential nutrients and saturated fats associated with CVD risk [33]. Fruits and vegetables continued to receive consistent attention, supported by robust evidence linking their high fiber and antioxidant content to cardiovascular benefits [34]. Conversely, ultra-processed foods emerged as a growing concern, with studies highlighting their association with obesity and metabolic disorders [35]. Research on alcohol declined, likely reflecting a consensus on its harmful effects [36], while studies on tea and coffee expanded, emphasizing their polyphenol content and potential anti-inflammatory effects [37]. These trends underscored a shift toward prioritizing food quality and functional properties over isolated dietary components.

In contrast, the number of studies focusing on single nutrients, such as lipids and minerals, declined over the years. This trend likely reflected a growing recognition that understanding the complex interplay of various dietary components of a diet was more significant for chronic disease relationships than focusing on isolated nutrients [38]. Studies of individual nutrients were often less representative of real-world dietary habits [9]. While poor nutrition contributed to the initiation and maintenance of the atherosclerotic process, diet also played a crucial role in reversing heart disease [39]. Furthermore, diets emphasizing single nutrients were found to lead to undesirable increases in the consumption of other nutrients (substitution effects) and associated foods [27]. The rising popularity of certain successful dietary patterns could be attributed to various factors, including evolutionary processes, cultural traditions unique to specific geographical and historical contexts, personal preferences, cost-effectiveness, seasonal availability of local food resources, and lifestyle choices [40].

### 4.3. Quality Assessment of Included Studies

NUQUEST revealed that most included studies demonstrated satisfactory quality, with strong cohort selection and comparability, though some had limitations in outcome ascertainment and nutrition-specific assessments. While variability exists, the overall robustness of the evidence supports the validity of the associations between diet and cardiovascular diseases. The distinct validity of different FFQs across nutrients and food groups could lead to inconsistent findings. This inconsistency may affect the reliability of conclusions drawn about the relationship between diet and CVD outcomes. It was observed that over 90% of the studies included claimed their FFQs were validated, but 4.4% of them did not provide references to any validation papers or validation metrics in text. Although the remaining included studies have cited validation papers, only about 20% aligned dietary exposure with validated nutrient factors. This discrepancy revealed a widespread misunderstanding of the intended use of FFQs, due to a conflation between the terms “validated” and “valid”. Researchers unfamiliar with FFQs may mistakenly assume “validated” FFQs were valid, unaware of the specific factors for which it was validated. Proper FFQ validation was critical because incorrect information could lead to false associations between dietary exposure and disease. This highlighted the importance for researchers to reference any validation papers and include key validation metrics in text to justify their use. Future research should prioritize standardized dietary assessment tools, rigorous follow-up protocols, and clear reporting of key validation metrics in measuring dietary exposures of interest in the methods section before implementation to reduce bias and enhance the reliability of dietary exposure measurements.

### 4.4. Strengths and Limitations

This systematic review has provided an updated summary of trends in diet–CVD research by study region and highlighted emerging dietary exposures of interest. The review adhered to PRISMA guidelines, and studies were included regardless of age and sex to minimize potential bias.

However, a few limitations should be acknowledged. First, the review focused exclusively on studies published within the last five years for feasibility, which still allowed for the identification of recent trends. Second, this review only evaluated studies that utilized FFQs, excluding those that employed other dietary assessment methods. Third, the current trend analysis may overlook emerging dietary patterns that warrant further investigation.

### 4.5. Implications for Future Research

While high-income countries continued to dominate this review, recent studies from rapidly developing nations provided crucial insights into how nutrition transitions impacted CVD risk profiles [41]. This diversity underscored the global relevance of such studies for the prevention and management of CVDs. However, the predominance of studies from high-income countries may have limited the generalizability of findings to low- and middle-income countries, where dietary transitions and CVD burdens were rapidly increasing [1,42]. The predominance of studies from the United States likely reflected the availability of extensive health databases and resources, but it also emphasized the need for more diverse population cohort studies to enhance the applicability of results across different cultural and dietary contexts. Significant challenges remained in translating this evidence into population-level behavioral change, particularly in resource-limited settings [43]. Future research should have prioritized diverse populations to address this gap and account for cultural, socioeconomic, and environmental influences on dietary habits and CVD risk [44].

The findings of this review held significant ramifications for public health initiatives aimed at reducing the risk of CVDs. Studies had shown a continuous increase in publications on dietary behaviors and CVDs over the past two decades [45]. Although studying dietary patterns had its own set of difficulties, including the categorization of food items and limitations of food composition databases [46], the importance of prioritizing dietary patterns over individual nutrients was emphasized.

Moreover, the observed trends in dietary exposure research suggested the need for future studies to delve deeper into the complexities of dietary patterns, including how cultural and socioeconomic elements defined the nuances of country-specific dietary patterns. Longitudinal studies that tracked changes in dietary patterns over time and their corresponding associations with CVD outcomes were essential for establishing causal relationships.

## 5. Conclusions

To conclude, this systematic review identified evolving trends in dietary research related to CVDs. The shift toward examining dietary patterns reflected a broader understanding of the role of nutrition in health, highlighting the need for comprehensive dietary guidelines and the importance of using validated dietary assessment tools. As research continues to evolve, it is essential to address the complexities of dietary habits and their implications for public health, ultimately aiming to reduce the global incidence of CVDs.

## Figures and Tables

**Table 1:** Search strategy used in this systematic review.

Databases	Keywords Used
PubMed	((“prospective cohort *” OR “prospective stud *” OR “retrospective cohort *” OR “retrospective stud *” OR “longitudinal stud *” OR “follow-up stud *”) AND (“cardiovascular disease” OR “CVD” OR “ischemic heart disease” OR “angina” OR “heart attack” OR “myocardial infarction” OR “coronary heart disease” OR “coronary artery disease” OR “CHD” OR “oedema” OR “edema” OR “heart failure” OR “HF” OR “stroke” OR “cerebrovascular disease” OR “atherosclerosis” OR “peripheral vascular disease” OR “peripheral arterial disease”)) AND (“food frequency questionnaire” OR “FFQ” OR “diet history questionnaire” OR “DHQ”) Filters: from 2019–2024
Web of Science	(((ALL = (“prospective cohort *” OR “prospective stud *” OR “retrospective cohort *” OR “retrospective stud *” OR “longitudinal stud *” OR “follow-up stud *”)) AND ALL = (“cardiovascular disease” OR “CVD” OR “ischemic heart disease” OR “angina” OR “heart attack” OR “myocardial infarction” OR “coronary heart disease” OR “coronary artery disease” OR “CHD” OR “oedema” OR “edema” OR “heart failure” OR “HF” OR “stroke” OR “cerebrovascular disease” OR “atherosclerosis” OR “peripheral vascular disease” OR “peripheral arterial disease”)) AND ALL = (“food frequency questionnaire” OR “FFQ” OR “diet history questionnaire” OR “DHQ”)) AND PY = (2019–2024)
Embase	(‘prospective cohort *’ OR ‘prospective stud *’ OR ‘retrospective cohort *’ OR ‘retrospective stud *’ OR ‘longitudinal stud *’ OR ‘follow-up stud *’) AND (‘cardiovascular disease’/exp OR ‘cardiovascular disease’ OR ‘cvd’ OR ‘ischemic heart disease’/exp OR ‘ischemic heart disease’ OR ‘angina’/exp OR ‘angina’ OR ‘heart attack’/exp OR ‘heart attack’ OR ‘myocardial infarction’/exp OR ‘myocardial infarction’ OR ‘coronary heart disease’/exp OR ‘coronary heart disease’ OR ‘coronary artery disease’/exp OR ‘coronary artery disease’ OR ‘chd’ OR ‘oedema’/exp OR ‘oedema’ OR ‘edema’/exp OR ‘edema’ OR ‘heart failure’/exp OR ‘heart failure’ OR ‘hf’/exp OR ‘hf’ OR ‘stroke’/exp OR ‘stroke’ OR ‘cerebrovascular disease’/exp OR ‘cerebrovascular disease’ OR ‘atherosclerosis’/exp OR ‘atherosclerosis’ OR ‘peripheral vascular disease’/exp OR ‘peripheral vascular disease’ OR ‘peripheral arterial disease’/exp OR ‘peripheral arterial disease’) AND (‘food frequency questionnaire’/exp OR ‘food frequency questionnaire’ OR ‘ffq’ OR ‘diet history questionnaire’/exp OR ‘diet history questionnaire’ OR ‘dhq’) AND [2019–2024]/py
Scopus	(TITLE-ABS-KEY (“prospective cohort *” OR “prospective stud *” OR “retrospective cohort *” OR “retrospective stud *” OR “longitudinal stud *” OR “follow-up stud *”) AND TITLE-ABS-KEY (“cardiovascular disease” OR “CVD” OR “ischemic heart disease” OR “angina” OR “heart attack” OR “myocardial infarction” OR “coronary heart disease” OR “coronary artery disease” OR “CHD” OR “oedema” OR “edema” OR “heart failure” OR “HF” OR “stroke” OR “cerebrovascular disease” OR “atherosclerosis” OR “peripheral vascular disease” OR “peripheral arterial disease”) AND TITLE-ABS-KEY (“food frequency questionnaire” OR “FFQ” OR “diet history questionnaire” OR “DHQ”)) AND PUBYEAR > 2018 AND PUBYEAR < 2025

Footnote: The * is a wildcard character used to represent variations of the term in search queries or text patterns.

**Table 2:** Countries of origin related to CVD–diet cohort studies between 2019 and 2024 (*n* = 298).

Country	Total [*n* (%)]	2019 [*n* (%)]	2020 [*n* (%)]	2021 [*n* (%)]	2022 [*n* (%)]	2023 [*n* (%)]	2024 [*n* (%)]
USA	92 (30.9%)	14 (29.8%)	19 (37.3%)	24 (36.4%)	17 (34.7%)	10 (20%)	8 (22.9%)
Japan	31 (10.4%)	7 (14.9%)	6 (11.8%)	10 (15.2%)	4 (8.2%)	4 (8%)	0 (0%)
Iran	27 (9.1%)	5 (10.6%)	5 (9.8%)	3 (4.5%)	6 (12.2%)	4 (8%)	4 (11.4%)
China	27 (9.1%)	1 (2.1%)	1 (2.0%)	5 (7.6%)	5 (10.2%)	10 (20%)	5 (14.3%)
Denmark	19 (6.4%)	4 (8.5%)	2 (3.9%)	6 (9.1%)	2 (4.1%)	4 (8%)	1 (2.9%)
South Korea	14 (4.7%)	2 (4.3%)	1 (2.0%)	2 (3.0%)	1 (2.0%)	6 (12%)	2 (5.7%)
Australia	12 (4.0%)	3 (6.4%)	2 (3.9%)	1 (1.5%)	2 (4.1%)	2 (4%)	2 (5.7%)
The Netherlands	9 (3.0%)	1 (2.1%)	1 (2.0%)	4 (6.1%)	1 (2.0%)	1 (2%)	1 (2.9%)
Greece	10 (3.4%)	1 (2.1%)	2 (3.9%)	1 (1.5%)	1 (2.0%)	2 (4%)	3 (8.6%)
Sweden	9 (3.0%)	2 (4.3%)	3 (5.9%)	0 (0%)	1 (2.0%)	1 (2%)	2 (5.7%)
Spain	8 (2.7%)	2 (4.3%)	0 (0%)	2 (3.0%)	4 (8.2%)	0 (0%)	0 (0%)
Norway	7 (2.3%)	0 (0%)	3 (5.9%)	1 (1.5%)	1 (2.0%)	0 (0%)	2 (5.7%)
Italy	5 (1.7%)	0 (0%)	1 (2.0%)	2 (3.0%)	0 (0%)	1 (2%)	1 (2.9%)
Others *	28 (9.4%)	5 (10.6%)	5 (9.8%)	5 (7.6%)	4 (8.2%)	5 (10%)	4 (11.4%)

* Countries with fewer than three studies, as well as combined regions (two or more countries or continents).

**Table 3:** Key study cohorts with higher contributions to this systematic review.

Cohort Name *	Frequency (%)
Nurses’ Health Study (NHS), NHSII and Health Professionals Follow-up Study (HPFS)	27 (9.1%)
Japan Public Health Center-based Prospective (JPHC) Study	19 (6.4%)
Danish Diet, Cancer, and Health cohort	16 (5.4%)
Tehran Lipid and Glucose Study (TLGS)	12 (4.0%)
ATTICA study	10 (3.4%)
Korean Genome and Epidemiology Study	9 (3.0%)
Million Veteran Program (MVP)	8 (2.7%)
National Institutes of Health (NIH)-AARP Diet and Health Study	8 (2.7%)
Isfahan Cohort Study	8 (2.7%)
Women’s Health Initiative (WHI)	8 (2.7%)

* Only cohorts contributing to eight or more papers are displayed.

**Table 4:** Types of dietary exposures in CVD–diet cohort studies between 2019 and 2024 (*n* = 298).

Dietary Exposure	Total [*n* (%)]
Dietary patterns	118 (39.6%)
Food groups	97 (32.6%)
Nutrients	77 (25.8%)
Nutrients and food groups	6 (2.0%)

**Table 5:** Dietary pattern exposure trends in CVD–diet cohort studies between 2019 and 2024.

Exposure *	Total [*n* (%)]	2019 [*n* (%)]	2020 [*n* (%)]	2021 [*n* (%)]	2022 [*n* (%)]	2023 [*n* (%)]	2024 [*n* (%)]
Dietary patterns (*n* = 92) *	^ 120 (100%)	16 (100%)	18 (100%)	21 (100%)	27 (100%)	15 (100%)	23 (100%)
Vegetarian or plant-based diet	22 (18.3%)	2 (12.5%)	3 (16.7%)	3 (14.3%)	4 (14.8%)	4 (26.7%)	6 (26.1%)
Mediterranean diet variations	18 (15.0%)	3 (18.8%)	3 (16.7%)	5 (23.8%)	4 (14.8%)	0 (0.0%)	3 (13.0%)
Country-specific indices	17 (14.2%)	2 (12.5%)	3 (16.7%)	4 (19.0%)	2 (7.4%)	1 (6.7%)	5 (21.7%)
Anti-inflammatory diet	11 (9.2%)	1 (6.3%)	4 (22.2%)	1 (4.8%)	2 (7.4%)	2 (13.3%)	1 (4.3%)
Diet quality indices	11 (9.2%)	0 (0.0%)	1 (5.6%)	0 (0.0%)	4 (14.8%)	4 (26.7%)	2 (8.7%)
DASH diet scores	9 (7.5%)	4 (25.0%)	0 (0.0%)	3 (14.3%)	1 (3.7%)	0 (0.0%)	1 (4.3%)
HEI variations	8 (6.7%)	2 (12.5%)	2 (11.1%)	2 (9.5%)	1 (3.7%)	0 (0.0%)	1 (4.3%)
Low-carbohydrate diet	3 (2.5%)	0 (0.0%)	1 (5.6%)	1 (4.8%)	1 (3.7%)	0 (0.0%)	0 (0.0%)
Others	21 (17.5%)	2 (12.5%)	1 (5.6%)	2 (9.5%)	8 (29.6%)	4 (26.7%)	4 (17.4%)

Abbreviations: DASH, Dietary Approaches to Stop Hypertension; HEI, Healthy Eating Index. * Unique research articles reporting dietary patterns—92; overlapping articles with multiple dietary patterns—28 [vegetarian or plant-based diets = 3, anti-inflammatory diets = 2, Mediterranean diet variations = 5, HEI variations = 3, DASH diet scores = 7, diet quality indices = 1, country-specific indices = 4, others = 3]; ^ total research articles = unique + overlapping research articles.

**Table 6:** Food group exposure trends in CVD–diet cohort studies between 2019 and 2024.

Exposure *	Total [*n* (%)]	2019 [*n* (%)]	2020 [*n* (%)]	2021 [*n* (%)]	2022 [*n* (%)]	2023 [*n* (%)]	2024 [*n* (%)]
Food groups (*n* = 125) *	^ 138 (100%)	22 (100%)	21 (100%)	45 (100%)	19 (100%)	19 (100%)	12 (100%)
Animal-source foods	42 (30.4%)	7 (31.8%)	8 (38.1%)	11 (24.4%)	7 (36.8%)	7 (36.8%)	2 (16.7%)
Fruits and vegetables	23 (16.7%)	4 (18.2%)	3 (14.3%)	7 (15.6%)	3 (15.8%)	3 (15.8%)	3 (25.0%)
Ultra-processed foods	15 (10.9%)	1 (4.5%)	1 (4.8%)	4 (8.9%)	1 (5.3%)	5 (26.3%)	3 (25.0%)
Nuts and seeds	10 (7.2%)	2 (9.1%)	2 (9.5%)	5 (11.1%)	1 (5.3%)	0 (0.0%)	0 (0.0%)
Legumes	9 (6.5%)	1 (4.5%)	1 (4.8%)	6 (13.3%)	1 (5.3%)	0 (0.0%)	0 (0.0%)
Sugars and sweetened beverages	9 (6.5%)	1 (4.5%)	3 (14.3%)	2 (4.4%)	1 (5.3%)	2 (10.5%)	0 (0.0%)
Tea and coffee	8 (5.8%)	2 (9.1%)	2 (9.5%)	3 (6.7%)	1 (5.3%)	0 (0.0%)	0 (0.0%)
Whole grains	5 (3.6%)	0 (0.0%)	0 (0.0%)	2 (4.4%)	2 (10.5%)	1 (5.3%)	0 (0.0%)
Unsaturated plant oils and spreads	4 (2.9%)	0 (0.0%)	1 (4.8%)	1 (2.2%)	1 (5.3%)	0 (0.0%)	1 (8.3%)
Saturated plant fats	2 (1.4%)	0 (0.0%)	0 (0.0%)	1 (2.2%)	0 (0.0%)	0 (0.0%)	1 (8.3%)
Alcohol	2 (1.4%)	1 (4.5%)	0 (0.0%)	0 (0.0%)	0 (0.0%)	0 (0.0%)	1 (8.3%)
Others	9 (6.5%)	3 (13.6%)	0 (0.0%)	3 (6.7%)	1 (5.3%)	1 (5.3%)	1 (8.3%)

* Unique research articles reporting food groups—125; overlapping articles with multiple food groups—13 [alcohol—1, animal-source foods—1, ultra-processed food—3, nuts and seeds—1, legumes—2, whole grains—1, fruits and vegetables—1, saturated plant fats—1, sugars and sweetened beverages—1, others—1]; ^ total research articles = unique + overlapping research articles.

**Table 7:** Nutrient exposure trends in CVD–diet cohort studies between 2019 and 2024.

Exposure *	Total [*n* (%)]	2019 [*n* (%)]	2020 [*n* (%)]	2021 [*n* (%)]	2022 [*n* (%)]	2023 [*n* (%)]	2024 [*n* (%)]
Nutrients (*n* = 87) *	^ 103 (100%)	18 (100%)	20 (100%)	26 (100%)	12 (100%)	17 (100%)	10 (100%)
Lipids	28 (27.2%)	7 (38.9%)	4 (20.0%)	9 (34.6%)	2 (16.7%)	3 (17.6%)	3 (30.0%)
Minerals	21 (20.4%)	6 (33.3%)	2 (10.0%)	4 (15.4%)	3 (25.0%)	4 (23.5%)	2 (20.0%)
Phytochemicals	17 (16.5%)	2 (11.1%)	3 (15.0%)	4 (15.4%)	2 (16.7%)	4 (23.5%)	2 (20.0%)
Proteins	11 (10.7%)	2 (11.1%)	3 (15.0%)	2 (7.7%)	1 (8.3%)	2 (11.8%)	1 (10.0%)
Vitamins	10 (9.7%)	1 (5.6%)	2 (10.0%)	4 (15.4%)	1 (8.3%)	1 (5.9%)	1 (10.0%)
Carbohydrates	8 (7.8%)	0 (0.0%)	1 (5.0%)	2 (7.7%)	2 (16.7%)	2 (11.8%)	1 (10.0%)
Others	8 (7.8%)	0 (0.0%)	5 (25.0%)	1 (3.8%)	1 (8.3%)	1 (5.9%)	0 (0.0%)

* Unique research articles reporting nutrients—87; overlapping articles with multiple nutrients—16 [vitamins—2, minerals—1, carbohydrates—5, proteins—4, lipids—2, others—2]; ^ total research articles = unique + overlapping research articles.

## Data Availability

The data presented in this study are available on request from the corresponding authors.

## Supplementary Materials

The following supporting information can be downloaded at https://www.mdpi.com/article/10.3390/nu17132126/s1, Table S1, Characteristics of included studies; Table S2, Quality assessment of included cohort studies.

## Abbreviations

The following abbreviations are used in this manuscript:

CVD(s)	Cardiovascular disease(s)
DASH	Dietary Approaches to Stop Hypertension
HEI	Healthy Eating Index
FFQs	Food frequency questionnaires
HPFS	Health Professionals Follow-up Study
JPHC	Japan Public Health Center-based Prospective Study
NUQUEST	Nutrition Quality Evaluation Strengthening Tools
NHS	Nurses’ Health Study
NHSⅡ	Nurses’ Health Study II
PRISMA	Preferred Reporting Items for Systematic reviews and Meta-Analyses
RoB	Risk of bias
TLGS	Tehran Lipid and Glucose Study
WHO	World Health Organization
