# Supplementary material for: Evolving Research Focus on Diet and Cardiovascular Disease: A Systematic Review of 298 Cohort Studies Published from 2019 to 2024
Source: Nutrients. 2025 Jun 26;17(13):2126. doi: 10.3390/nu17132126 (PMC12252054; doi:10.3390/nu17132126)
Supplement: Supplementary file 1 [file nutrients-17-02126-s001.zip › nutrients-3695591-supplementary.pdf]

Supplementary Table S1. Characteristics of included studies (n=298).

Author, year	Country	Study design and mean/median follow-up period	Cohort used	No. of participants (% of male) and age at baseline	Dietary exposure and assessment tool	CVD outcomes
Abris et al., 2024 (1)	USA	Prospective cohort studies with median follow-up 11.1 years	Adventist Health Study-2 (AHS-2)	88,400 (35%); 30 years or above	Vegetarian Diet; FFQ	Mortality from ischemic heart disease, select cardiac disease, and other cardiovascular diseases
Akter et al., 2021 (2)	Japan	Prospective cohort studies with median follow-up 16.9 years	Japan Public Health Center–based Prospective (JPHC) Study	93,654 (46%); 45-75 years	Low-carbohydrate diet; 147-item FFQ	Cardiovascular disease mortality
Al-Ramady et al., 2022 (3)	USA	Prospective cohort studies with mean follow-up 3.3 years	Million Veteran Program (MVP)	233,792 (92%); 66 ± 12 years	Egg consumption; Willet semi-quantitative FFQ	Incidence of fatal and non-fatal ischemic stroke
Al-Shaar et al., 2020 (4)	USA	Prospective cohort studies with mean follow-up 23.7 years	Health Professionals Follow-up Study	43,272 (100%); 53 ± 10 years	Red meat intake; semiquantitative FFQ	Incidence of coronary heart disease
Amba et al., 2019 (5)	USA	Prospective cohort studies with median follow-up 15.5 years	National Institutes of Health-AARP (NIH-AARP) Diet and Health Study	566,398 (56%); 50–71 years	Nut and peanut butter consumption; 124-item FFQ	Cardiovascular disease mortality
Arnesen et al., 2024a (6)	Norway	Prospective cohort studies with median follow-up 33.5 years	Norwegian Counties Study	77,297 (not reported); 18-64 years	Potato consumption; semi-quantitative FFQ	Cardiovascular disease mortality
Arnesen et al., 2024b (7)	Norway	Prospective cohort studies with median follow-up 33.5 years	Norwegian Counties Study	78,725 (50%); 20-49 years	Saturated fatty acids; semi-quantitative FFQ	Cardiovascular disease mortality
Asadi et al., 2019 (8)	Iran	Prospective cohort studies with mean follow-up 6 years	Mashhad stroke and heart atherosclerotic disorder (MASHAD) study	5,706 (41%); 35-76 years	Balanced dietary pattern and western dietary pattern; 65-item FFQ	Incidence of cardiovascular events
Asadi et al., 2020 (9)	Iran	Prospective cohort studies with mean follow-up 5-6 years	Mashhad stroke and heart atherosclerotic disorder (MASHAD) study	4,672 (42%); 35–65 years	Dietary inflammatory index (DII); 65-item FFQ	Incidence of cardiovascular diseases
Asghari et al., 2022 (10)	Iran	Prospective cohort studies with median follow-up 6.7 years	Tehran Lipid and Glucose Study (TLGS)	2,195 (45%); 39 ±13 years	Dietary Diabetes Risk Reduction Score; 168-item FFQ	Incidence of cardiovascular diseases, coronary heart disease events, stroke
Atefatfar et al., 2023 (11)	Iran	Prospective cohort studies with median follow-up 17 years	Isfahan Cohort Study (ICS)	6,504 (51%); 35 years or above	Dietary quality index (DQI); 8-item FFQ	Cardiovascular disease mortality
Baden et al., 2019 (12)	USA	Prospective cohort studies (follow-up period not reported)	Nurses' health study (NHS), NHSII, and Health Professionals Follow-up Study (HPFS)	75,314 (34%); 30-75 years	Plant-based diet index; FFQ	Cardiovascular disease mortality
Bahadoran et al., 2022 (13)	Iran	Prospective cohort studies with median follow-up 10.6 years	Tehran Lipid and Glucose Study (TLGS)	2,966 (45%); 39 ± 14 years	Dietary oxalate to calcium ratio; 168-item FFQ	Incidence of cardiovascular events
Bellinge et al., 2021 (14)	Denmark	Prospective cohort studies with mean follow-up 21 years	Danish Diet, Cancer, and Health cohort	53,372 (47%); 52–60 years	Vitamin K intake; 192-item semi-quantitative FFQ	Incidence of atherosclerotic cardiovascular disease
Blekkenhorst et al., 2020 (15)	Australia	Prospective cohort studies with median follow-up 15 years	Prospective population-based cohort study (PLSAW)	1,226 (0%); 75 ± 3 years	Vegetable diversity; semi-quantitative FFQ	Atherosclerotic vascular disease mortality
Bodar et al., 2020 (16)	USA	Prospective cohort studies with mean follow-up 9.3 years	Physicians' Health Study	20,433 (100%); 66 ± 9 years	Coffee consumption; semiquantitative FFQ	Incidence of heart failure
Bodar et al., 2021 (17)	USA	Prospective cohort studies with mean follow-up 2.7 years	Million Veteran Program (MVP)	148,671 (90%); 64 ± 12 years	Consumption of potatoes; semiquantitative FFQ	Incidence of coronary artery disease
Bonaccio et al., 2023 (18)	Italy	Prospective cohort studies with median follow-up 11.6 years	Moli-sani Study	1,065 (61%); 35 years or above	Ultra-processed food consumption; 188-item FFQ	Cardiovascular disease mortality
Bonekamp et al., 2024 (19)	The Netherlands	Prospective cohort studies with median follow-up 8.7 years	Utrecht Cardiovascular Cohort-Secondary Manifestations of ARTerial disease (UCC-SMART) prospective cohort study	2,656 (77%); 59 ± 9 years	The Dutch Healthy Diet 2015 (DHD-15) index; 106-item FFQ	Incidence of cardiovascular disease, MI, Stroke
Bork et al., 2019 (20)	Denmark	Prospective cohort studies with median follow-up 13.6 years	Danish Diet, Cancer, and Health cohort	55,248 (48%); 50–64 years	Intake of α-linolenic acid; 192-item semi-quantitative FFQ	Incidence of peripheral artery disease
Bork et al., 2023 (21)	Denmark	Prospective cohort studies with median follow-up 13.5 years	Danish Diet, Cancer, and Health cohort	53,909 (47%); 50-64 years	Intake of marine and plant-derived n-3 fatty acids; 192-item FFQ	Incidence of atherosclerotic cardiovascular disease
Budhathoki et al., 2019 (22)	Japan	Prospective cohort studies with mean follow-up 18 years	Japan Public Health Center–based Prospective (JPHC) Study	70,696 (46%); 45-74 years	Animal and plant protein intake; 138-item FFQ	Cardiovascular disease mortality
Bui et al., 2024 (23)	USA	Prospective cohort studies with mean follow-up up to 34 years	Nurses' health study (NHS), NHSII, and Health Professionals Follow-up Study (HPFS)	206,404 (23%); 25-75 years	Planetary Health Diet Index; semiquantitative FFQ	Cardiovascular disease mortality
Buziau et al., 2019 (24)	Australia	Prospective cohort studies with mean follow-up 15 years	Australian Longitudinal Study on Women's Health (ALSWH).	7,633 (0%); 45-50 years	Fermented dairy consumption; 101-item FFQ	Incidence of cardiovascular disease
Carballo-Casla et al., 2024 (25)	Spain, UK, Czechia and Poland	Prospective cohort studies with median follow-up 13.6 years	ENRICA, HAPIEE and Whitehall II	35,917 (51%); 18–96 years	Southern European Atlantic diet; semi-quantitative FFQ and electronic diet history	Cardiovascular disease mortality
Carroll et al., 2024 (26)	Sweden	Prospective cohort studies with mean follow-up 20 years	Malmö Diet and Cancer Study (MDCS)	25,369 (38%); 51-64 years	Water intake; 168-item FFQ	Incidence of coronary artery disease
Castañeda et al., 2024 (27)	Mexico	Prospective cohort studies with median follow-up 11.2 years	Mexican Teachers' Cohort	95,313 (0%); not reported	Dietary Intake of (Poly)phenols; 140-item FFQ	Cardiovascular disease mortality
Chan et al., 2019 (28)	China	Prospective cohort studies with mean follow-up 13.8 years	Mr. OS and Ms. OS study	3,020 (49%); 65 years or above	Protein intake; 280-item FFQ	Cardiovascular disease mortality
Chen et al., 2021 (29)	USA	Prospective cohort studies with median follow-up 17.8 years	Women's Health Initiative (WHI)	96,831 (0%); 50 - 79 years	Dietary cholesterol and egg intake; 122-item FFQ	Incidence of cardiovascular disease, ischemic heart disease, hemorrhagic stroke, ischemic stroke, and cardiovascular disease mortality
Chen et al., 2023a (30)	USA	Prospective cohort studies with mean follow-up over 30 years	Nurses' Health Study (NHS) and Health Professionals Follow-up Study (HPFS)	15,040 (not reported); 30-75 years	Dietary glutamine and glutamate; ~131-item semiquantitative FFQ	Incidence of cardiovascular disease, coronary heart disease, stroke, and cardiovascular mortality
Chen et al., 2023b (31)	USA	Prospective cohort studies with mean follow-up up to 30 years	Nurses' Health Study (NHS) and Health Professionals Follow-up Study (HPFS)	119,982 (37%); 30 - 75 years	Dietary phytoestrogens; 116-item FFQ	Cardiovascular disease mortality
Chiu et al., 2020 (32)	Taiwan	Prospective cohort studies with median follow-up 7.5 years	Tzu Chi Health Study, Tzu Chi Vegetarian Study	13,352 (38%); 50 years	Vegetarian diet; quantitative FFQ	Incidence of ischemic and hemorrhagic strokes
Chuang et al., 2021 (33)	Taiwan	Prospective cohort studies with mean follow-up 17.8 years	Nutrition and Health Survey	2,475 (47%); 18–65 years	Healthy Taiwanese Eating Approach; 28-item FFQ	Cardiovascular disease mortality
Chung et al., 2023 (34)	South Korea	Prospective cohort studies with mean follow-up 11.6 years	Ansan–Ansung cohort study, Health Examinees (HEXA) study, and Cardiovascular Disease Association Study (CAVAS)	134,586 (35%); 40 - 69 years	Meat and fish/seafood intake; Semiquantitative FFQ	Cardiovascular disease mortality

Author, year	Country	Study design and mean/median follow-up period	Cohort used	No. of participants (% of male) and age at baseline	Dietary exposure and assessment tool	CVD outcomes
Colizzi et al., 2023 (35)	The Netherlands	Prospective cohort studies with median follow-up 15.1 years	European Prospective Investigation into Cancer and Nutrition-Netherlands cohort (EPIC-NL)	35,496 (26%); 20-70 years	EAT-Lancet healthy reference diet; 178-item FFQ	Incidence of cardiovascular disease, coronary heart disease, and stroke
Cordova et al., 2023 (36)	Seven countries (Denmark, the Netherlands, Spain, Sweden, the UK, Italy, Germany)	Prospective cohort studies with median follow-up 11.2 years	European Prospective Investigation into Cancer and Nutrition cohort (EPIC)	266,666 (40%); 52 ± 10 years	Ultra-processed food consumption; country-specific FFQ	Incidence of cardiovascular diseases
Critselis et al., 2021 (37)	Greece	Prospective cohort studies with mean follow-up 10 years	ATTICA study	2,020 (50%); 45 ± 14 years	Mediterranean diet and Dietary approaches to stop hypertension (DASH) diet; semiquantitative FFQ	Incidence of cardiovascular disease
Critselis et al., 2023 (38)	Greece	Prospective cohort studies with mean follow-up 20 years	ATTICA study	2,020 (50%); 45 ± 14 years	Fish intake rich in n-3 fatty acids; 156-item semi-quantitative FFQ	Incidence of cardiovascular disease
Cruijsen et al., 2021 (39)	The Netherlands	Prospective cohort studies with mean follow-up 12 years	Alpha Omega Cohort (AOC)	4,365 (79%); 69 ± 6 years	Dairy consumption; 203-item FFQ	Cardiovascular disease, ischemic heart disease, and stroke mortality
Cui et al., 2022 (40)	China	Prospective cohort studies with mean follow-up 4.6 years	Shanghai Suburban Adult Cohort and Biobank (SSACB) study and a cohort study in Minhang district, Shanghai	57,701 (40%); 51–65 years	Fish consumption; 29-item FFQ	Incidence of total stroke, ischemic stroke and hemorrhagic stroke
Cupino et al., 2022 (41)	USA	Prospective cohort studies with mean follow-up 11.2 years	Adventist Health Study-2 (AHS-2)	78,335 (35%); 30 years or above	Total omega-3 and omega-6 polyunsaturated fatty acids; FFQ	Incidence of fatal Stroke
Dalgaard et al., 2019 (42)	Denmark	Prospective cohort studies with median follow-up 21 years	Danish Diet, Cancer, and Health cohort	53,552 (47%); 52 - 60 years	Dietary isoflavonoid intake; 192-item FFQ	Atherosclerotic cardiovascular disease hospital admissions
Damigou et al., 2023 (43)	Greece	Prospective cohort studies with total follow-up 20 years	ATTICA study	2,169 (not reported); 44 ± 14 years	Global diet quality score (GDQS); 156-item FFQ	Incidence of cardiovascular disease
Damigou et al., 2024a (44)	Greece	Prospective cohort studies with mean follow-up 10 to 20 years	ATTICA study	759 (51%); 40 ± 11 years	Food Compass Score; 156-item semi-quantitative FFQ	Incidence of cardiovascular disease
Damigou et al., 2024b (45)	Greece	Prospective cohort studies with mean follow-up 20 years	ATTICA study	313 (57%); >18 years	Mediterranean diet; FFQ	Incidence of cardiovascular disease
Das et al., 2021 (46)	Australia	Prospective cohort studies with median follow-up 5.2 years	Concord Health and Ageing in Men Project (CHAMP)	794 (100%); 81 ± 5 years	Dietary antioxidant intake; DHQ	Incidence of major adverse cardiovascular events (MACE) (death, MI, ischemic stroke, congestive cardiac failure (CCF), and revascularization)
Das et al., 2022 (47)	Australia	Prospective cohort studies with median follow-up 3.7 years	Concord Health and Ageing in Men Project (CHAMP)	782 (100%); 81 years	Dietary intake of total protein and sources of protein; DHQ	Cardiovascular disease mortality
Davitte et al., 2021 (48)	USA	Prospective cohort studies with mean follow-up 13.5 ± 6.2 years	Rancho Bernardo Study	1,363 (40%); 71 ± 11 years	Potassium intake; 153-item semiquantitative Willett FFQ	Cardiovascular disease mortality and stroke mortality
de la O et al., 2022 (49)	Spain	Prospective cohort studies with median follow-up 12.2 years	Seguimiento University of Navarra (SUN) project	18,210 (61%); 38 ± 12 years	Paleolithic diet and mediterranean diet scores; 136-item semi-quantitative FFQ	Incidence of Cardiovascular disease, myocardial infarction, non-fatal stroke, and cardiovascular disease mortality
de Souza et al., 2020 (50)	16 countries	Prospective cohort studies with median follow-up 9.5 years	Prospective Urban and Rural Epidemiological (PURE) study	124,329 (42%); 51±10 years	Nut consumption; country-specific FFQ	Incidence of cardiovascular diseases and cardiovascular disease mortality
Dehghan et al., 2020 (51)	21 countries	Prospective cohort studies with median follow-up 9.5 years	Prospective Urban and Rural Epidemiological (PURE) study	146,011 (42%); 51 ± 10 years	Egg intake; country-specific FFQ	Cardiovascular disease mortality
Dehghan et al., 2023 (52)	21 countries	Prospective cohort studies with median follow-up 10.2 years	Prospective Urban and Rural Epidemiological (PURE) study	138,076 (42%); 35-70 years	Ultra-processed food consumption; semiquantitative FFQ	Incidence of cardiovascular diseases and cardiovascular disease mortality
Dennis et al., 2023 (53)	USA	Prospective cohort studies with mean follow-up up to 40 years	Nurses' Health Study (NHS) and Health Professionals Follow-up Study (HPFS)	115,693 (34%); not reported	Dietary sugar; semiquantitative FFQ	Incidence of coronary heart disease and mortality
Devaraj et al., 2021 (54)	USA	Prospective cohort studies with mean follow-up 30 years	Pittsburgh Epidemiology of Diabetes Complications (EDC) cohort	514 (50%); 28 years	Patterns of nutrient intake; FFQ	Incidence of Coronary artery disease
Dibaba et al., 2019 (55)	USA	Prospective cohort studies with mean follow-up 8.3 ± 3.2 years	REasons for Geographic and Racial Differences in Stroke (REGARDS) study	19,553 (not reported); 65 years	Calcium intake; Block 98 FFQ	Incidence of ischemic stroke
Djoussé et al., 2020 (56)	USA	Prospective cohort studies with mean follow-up 3.2 years	Million Veteran Program (MVP)	188,267 (90%); 64 ± 12 years	Egg consumption; Willet semi-quantitative FFQ	Incidence of fatal and non-fatal myocardial infarction
Djoussé et al., 2021 (57)	USA	Prospective cohort studies with mean follow-up 7 to 20.8 years	Atherosclerosis Risk in Communities (ARIC), Cardiovascular Health Study (CHS), The Jackson Heart Study (JHS), Multi-Ethnic Study of Atherosclerosis (MESA), Physicians' Health Study (PHS), Women's Antioxidant Cardiovascular Study (WACS), Women's Health Study (WHS)	103,811 (35%); 53-78 years	Egg consumption; FFQ or diet history	Incidence of coronary heart disease
Donat-Vargas et al., 2020 (58)	Sweden	Prospective cohort studies with mean follow-up 15.5 years	Swedish Mammography Cohort (SMC) and Cohort of Swedish Men (COSM)	69,497 (53%); not reported	Dietary exposure to polychlorinated biphenyls (PCBs) and long-chain omega-3 fish fatty acids intake; 96-item FFQ	Cardiovascular disease mortality
Du et al., 2021 (59)	USA	Prospective cohort studies with median follow-up 27 years	Atherosclerosis Risk in Communities (ARIC) study	13,548 (44%); 56 years	Ultra-processed food consumption; 66-item FFQ	Incidence of coronary artery disease
Dupuy et al., 2024 (60)	Australia	Prospective cohort studies with median follow-up 15 years	Perth Longitudinal Study of Ageing Women	1,436 (0%); 75 ± 3 years	Vitamin K1 intake; semiquantitative FFQ	Cardiovascular disease mortality
Ergas et al., 2024 (61)	USA	Prospective cohort studies with mean follow-up 11.5 years	Pathways Study	3,415 (0%); 60 ± 12 years	Dietary Approaches to Stop Hypertension (DASH), healthy plant-based dietary index, 2020 Healthy Eating Index (HEI), American Cancer Society (ACS) nutrition guidelines for cancer prevention, and alternate Mediterranean dietary index; 139-item FFQ	Incidence of cardiovascular disease events (ischemic heart disease, heart failure, cardiomyopathy, and stroke)
Evers et al., 2022 (62)	The Netherlands	Prospective cohort studies with median follow-up 12.4 years	Alpha Omega Cohort (AOC)	4,365 (79%); 69 ± 6 years	Dietary magnesium; 203-item FFQ	Cardiovascular disease and coronary heart disease mortality
Fang et al., 2024 (63)	USA	Prospective cohort studies with median follow-up 34 years	Nurses' Health Study (NHS) and Health Professionals Follow-up Study (HPFS)	111,064 (36%); 64 - 66 years	Ultra-processed food consumption; semiquantitative FFQ	Cardiovascular disease mortality
Farhadnejad et al., 2020 (64)	Iran	Prospective cohort studies with mean follow-up 6.7 years	Tehran Lipid and Glucose Study (TLGS)	2,188 (45%); 39 ± 13 years	Low-carbohydrate diet; 168-item semi-quantitative FFQ	Incidence of cardiovascular disease

Author, year	Country	Study design and mean/median follow-up period	Cohort used	No. of participants (% of male) and age at baseline	Dietary exposure and assessment tool	CVD outcomes
Frederiksen et al., 2021 (65)	Denmark	Prospective cohort studies with median follow-up 13.5 years	Danish Diet, Cancer, and Health cohort	55,061 (48%); 50 - 64 years	Prudent pattern, a western pattern and a wine, and snacks pattern; 192-item semiquantitative FFQ	Incidence of stroke
Fruh et al., 2024 (66)	Denmark	Prospective cohort studies with median follow-up 13.6 to 19.8 years	Danish Diet, Cancer, and Health cohort	19,394 (53%); 50 - 64 years	Combined dietary intake (CDI) of magnesium (Mg), calcium (Ca), and potassium (K); 192-item semiquantitative FFQ	Incidence of heart failure, acute myocardial infarction, and stroke
Fu et al., 2024 (67)	China	Prospective cohort studies (follow-up period not reported)	Beijing MJ Health Screening Center	13,989 (61%); 43 ± 9 years	a priori dietary pattern and a posteriori dietary pattern; 25-item semiquantitative FFQ	Carotid atherosclerosis (CAS)
Gaeini et al., 2019 (68)	Iran	Prospective cohort studies with median follow-up 6 years	Tehran Lipid and Glucose Study (TLGS)	6,027 (43%); 19 years or above	Consumption of coffee and tea; 168-item FFQ	Incidence of cardiovascular disease
Gaeini et al., 2021 (69)	Iran	Prospective cohort studies with mean follow-up 10.6 years	Tehran Lipid and Glucose Study (TLGS)	2,809 (44%); 19 years or above	Dietary fats intake; 168-item semi-quantitative FFQ	Incidence of cardiovascular disease
Gamba et al., 2023 (70)	Switzerland	Prospective cohort studies with median follow-up 9 years	The CoLaus study	3,721 (45.2%); 57 ± 10 years	Dietary Phytochemical Index (DPI); 97-item FFQ	Incidence of cardiovascular disease and mortality
Ganbat et al., 2024 (71)	South Korea	Prospective cohort studies with median follow-up 4.2 years	Health Examinees study (HEXA)	12,356 (100%); 40 years or above	Plant-based diet index; 108-item semiquantitative FFQ	Incidence of coronary heart disease
Gao et al., 2021a (72)	Japan	Prospective cohort studies with median follow-up 13.1 years	Japan Public Health Center–based Prospective (JPHC) Study	87,177 (46%); 44-76 years	Consumption of flavonoid-rich fruits, flavonoids from fruits; 138-item FFQ	Incidence of Stroke
Gao et al., 2021b (73)	Japan	Prospective cohort studies with median follow-up 19.3 years	Japan Collaborative Cohort Study (JACC)	58,646 (39%); 40-79 years	Dietary carotene intake; 40-item FFQ	Cardiovascular disease mortality
Ge et al., 2023 (74)	Japan	Prospective cohort studies with mean follow-up 19.3 years	Japan Public Health Center–based Prospective (JPHC) Study	93,310 (46%); 55-57 years	Dairy intake; 147-item FFQ	Cardiovascular disease, heart disease, and cerebrovascular disease mortality
Georgoulis et al., 2024 (75)	Greece	Prospective cohort studies with mean follow-up 20 years	ATTICA study	1,988 (50%); 35-65 years	Adherence to the Mediterranean diet; 156-item semi-quantitative FFQ	Incidence of cardiovascular disease
Glenn et al., 2021 (76)	USA	Prospective cohort studies with mean follow-up 15.3 years	Women's Health Initiative (WHI)	123,330 (0%); 50-79 years	Plant-Based Dietary Portfolio; FFQ	Incidence of cardiovascular diseases
Glenn et al., 2023 (77)	USA	Prospective cohort studies with mean follow-up up to 30 years	Nurses' health study (NHS), NHSII, and Health Professionals Follow-up Study (HPFS)	210,240 (21%); 25-75 years	Portfolio Diet Score; semiquantitative FFQ	Incidence of total cardiovascular disease, coronary heart disease and stroke
Golzarand et al., 2022 (78)	Iran	Prospective cohort studies with median follow-up 10.6 years	Tehran Lipid and Glucose Study (TLGS)	2,863 (44%); not reported	The Mediterranean-DASH intervention for neurodegenerative delay (MIND) diet; 168-item FFQ	Incidence of coronary heart disease (CHD), stroke, and cardiovascular disease related mortality
Gómez-Donoso et al., 2021 (79)	Spain	Prospective cohort studies with median follow-up 10.9 years	Seguimiento University of Navarra (SUN) project	20,503 (39%); 38 ± 12 years	Food standards agency nutrient profiling system (FSAm-NPS); 136-item semiquantitative FFQ	Cardiovascular disease mortality
Grau et al., 2022 (80)	Iran	Prospective cohort studies with median follow-up 11.2 years	Isfahan Cohort Study (ICS)	5,432 (not reported); 35 years or above	Red and processed meat consumption; 48-item FFQ	Incidence of cardiovascular disease and mortality
Gu et al., 2022 (81)	USA	Prospective cohort studies with mean follow-up 20 years	Health Professionals Follow-up Study	44,525 (100%); 40–75 years	Alternative Healthy Eating Index (AHEI), Prime Diet Quality Score (PDQS); 131- to 152-item semiquantitative FFQ	Incidence of congestive heart failure (HF), HF with preserved ejection fraction and HF with reduced ejection fraction
Guasch-Ferré et al., 2019 (82)	USA	Prospective cohort studies with median follow-up 22 years	Nurses' Health Study (NHS) and Health Professionals Follow-up Study (HPFS)	93,378 (32%); 30-75 years	Monounsaturated fatty acids from plant and animal sources; >130-item FFQ	Cardiovascular disease mortality
Guasch-Ferre et al., 2020 (83)	USA	Prospective cohort studies with mean follow-up 24 years	Nurses' Health Study (NHS) and Health Professionals Follow-up Study (HPFS)	92,978 (34%); 30-75 years	Olive oil intake; >130-item semiquantitative FFQ	Incidence of total cardiovascular disease, coronary heart disease and stroke
Guasch-Ferré et al., 2022 (84)	USA	Prospective cohort studies with mean follow-up up to 28 years	Nurses' Health Study (NHS) and Health Professionals Follow-up Study (HPFS)	92,383 (34%); 30-75 years	Consumption of olive oil; >138-item FFQ	Cardiovascular disease mortality
Hansen et al., 2021 (85)	Denmark	Prospective cohort studies with mean follow-up 13.5 years	Danish Diet, Cancer, and Health cohort	57,053 (48%); 50–64 years	Substitutions between potatoes and other vegetables; 192-item semi-quantitative FFQ	Incidence of ischemic stroke
Harbers et al., 2020 (86)	Netherlands	Prospective cohort studies with median follow-up 15.2 years	European Prospective Investigation into Cancer and Nutrition-Netherlands cohort (EPIC-NL)	37,468 (not reported); 20 - 70 years	Dutch healthy diet 2015 index (DHD15-index); 178-item FFQ	Incidence of heart failure (HF) and mortality
Hashemian et al., 2019a (87)	Iran	Prospective cohort studies with mean follow-up 10.6 years	Golestan Cohort Study (GCS)	42,373 (42%); 52 ± 9 years	Healthy Eating Index (HEI-2015), Alternative Healthy Eating Index (AHEI-2010), Alternative Mediterranean Diet (AMED), Dietary Approach to Stop Hypertension created by Fung (DASH-Fung) and Mellen (DASH-Mellen), and the World Cancer Research Fund (WCRF/AICR) index; 116-item FFQ	Cardiovascular disease mortality
Hashemian et al., 2019b (88)	Iran	Prospective cohort studies with mean follow-up 11 years	Golestan Cohort Study (GCS)	44,398 (not reported); 40-75 years	Spice consumption; 116-item FFQ	Incidence of cardiovascular diseases and cardiovascular disease mortality
Haugsgjerd et al., 2020 (89)	Norway	Prospective cohort studies with median follow-up 11 years	The Hordaland Health Study Cohort	2,987 (43%); 48 years	Dietary vitamin K; FFQ	Incidence of coronary heart disease
Haugsgjerd et al., 2022 (90)	Norway	Prospective cohort studies with mean follow-up 10.8 years	Hordaland Health Study (HUSK)	2,995 (43%); 48 ± 1 years	Carbohydrates and saturated fatty acid intake; 169-item semi-quantitative FFQ	Incidence of fatal or non-fatal coronary heart disease
Hejazi et al., 2020 (91)	Iran	Prospective cohort studies with mean follow-up 10.6 years	Golestan Cohort Study (GCS)	42,605 (43%); 40–75 years	Dietary intake of flavonoids; 116-item semi-quantitative FFQ	Cardiovascular disease mortality
Hirahatake et al., 2019 (92)	USA	Prospective cohort studies with mean follow-up 12.4 years	Women's Health Initiative (WHI)	5,809 (0%); 64 ± 7 years	The alternate Mediterranean (aMed), DASH diets, the American Diabetes Association (ADA) dietary recommendations, and a Paleolithic (Paleo) diet; FFQ	Incidence of cardiovascular disease, coronary heart disease, and stroke
Hjelmgaard et al., 2023 (93)	Denmark	Prospective cohort studies with median follow-up 13.5 years	Danish Diet, Cancer, and Health cohort	394 (52%); 58 years	Intake of marine n-3 polyunsaturated fatty acids; 192-item semiquantitative FFQ	Incident hemorrhagic stroke, intracerebral hemorrhage and subarachnoid hemorrhage
Horikawa et al., 2019 (94)	Japan	Prospective cohort studies with mean follow-up 8 years	Japan Diabetes Complications Study (JDCS)	1,353 (53%); 40-70 years	Meat intake; FFQ based on food groups	Incidence of cardiovascular disease
Horikawa et al., 2021 (95)	Japan	Prospective cohort studies with mean follow-up 6 years	Japanese Elderly Diabetes Intervention Study (J-EDIT)	912 (45%); 65–85 years	Sodium intake; FFQ	Cardiovascular disease mortality
Hosseini-Esfahani et al., 2020 (96)	Iran	Prospective cohort studies with mean follow-up 5.3 years	Tehran Lipid and Glucose Study (TLGS)	5,102 (44%); 20-70 years	Intake of carbohydrates, protein, fat; 168-item semi-quantitative FFQ	Incidence of cardiovascular disease
Hu et al., 2019 (97)	USA	Prospective cohort studies with median follow-up 24 years	Atherosclerosis Risk in Communities (ARIC) study	12,413 (44%); 45–64 years	Healthy Eating Index (HEI)-2015, Alternative Healthy Eating Index–2010 (AHEI-2010), alternate Mediterranean (aMed) diet, and Dietary	Incidence cardiovascular disease and mortality

Author, year	Country	Study design and mean/median follow-up period	Cohort used	No. of participants (% of male) and age at baseline	Dietary exposure and assessment tool	CVD outcomes
					Approaches to Stop Hypertension diet (DASH); 66-item FFQ	
Hu et al., 2020 (98)	USA	Prospective cohort studies with median follow-up 7 years	Chronic Renal Insufficiency Cohort (CRIC)	2,283 (94%); 21-74 years	Healthy beverage score; 124-item diet history questionnaire	Incidence of myocardial infarction (MI), congestive heart failure (CHF), stroke and cardiovascular disease mortality
Hu et al., 2021 (99)	USA	Prospective cohort studies with median follow-up 25.8 years	Nurses' health study (NHS), NHSII, and Health Professionals Follow-up Study (HPFS)	201,300 (19%); 25–75 years	Lignan Intake; FFQ	Incidence of non-fatal myocardial infarction and fatal coronary heart disease
Hu et al., 2022 (100)	USA	Prospective cohort studies with mean follow-up 25.8 years	Nurses' health study (NHS), NHSII, and Health Professionals Follow-up Study (HPFS)	205,129 (19%); 25-75 years	Intake of whole grain foods; semi-quantitative food frequency questionnaire	Incidence of coronary heart disease
Huang et al., 2020 (101)	USA	Prospective cohort studies with median follow-up 15.5 years	National Institutes of Health-AARP (NIH-AARP) Diet and Health Study	406,104 (57%); 62 years	Animal and plant protein intake; 124-item DHQ	Cardiovascular disease mortality
Huang et al., 2021a (102)	Japan	Prospective cohort studies with mean follow-up 17.1 years	Japan Public Health Center–based Prospective (JPHC) Study	70,486 (44%); 45 - 74 years	Sugary drink consumption; 138-item FFQ	Heart disease and cerebrovascular disease mortality
Huang et al., 2021b (103)	USA	Prospective cohort studies with mean follow-up up to 20 years	Women's Health Initiative (WHI)	84,555 (0%); 50–79 years	Pasta meal intake; semiquantitative FFQ	Incidence of coronary heart disease and stroke
Ibsen et al., 2022 (104)	Denmark	Prospective cohort studies with median follow-up 15 years	Danish Diet, Cancer, and Health cohort	55,016 (48%); 50 - 64 years	EAT-Lancet diet; 192-item FFQ	Incidence of total stroke, ischemic stroke, intracerebral hemorrhage, subarachnoid hemorrhage
Ikehara et al., 2021 (105)	Japan	Prospective cohort studies with median follow-up 14.8 years	Japan Public Health Center–based Prospective (JPHC) Study	74,793 (47%); 45-74 years	Peanut consumption; 138-item FFQ	Incidence of cardiovascular disease
Im et al., 2021 (106)	South Korea	Prospective cohort studies with mean follow-up 7.4 years	Ansan–Ansung cohort study	4,713 (0%); 40 - 69 years	Soy food and soy isoflavones; semiquantitative FFQ	Incidence of cardiovascular disease
Imran et al., 2021 (107)	USA	Prospective cohort studies with mean follow-up 19 years	Women's Health Study	39,167 (0%); 39–90 years	Nut consumption; FFQ	Cardiovascular disease mortality
Ivey et al., 2021 (108)	USA	Prospective cohort studies with median follow-up 3.5 years	Million Veteran Program (MVP)	179,827 (90%); 64 ± 12 years	Nut consumption; 67-item FFQ	Incidence of ischemic stroke, hemorrhagic stroke, coronary artery disease and cardiovascular disease mortality.
Jackson et al., 2019a (109)	USA	Prospective cohort studies with mean follow-up up to 26 years	Nurses' health study (NHS)	62,535 (0%); 30 - 55 years	Dietary nitrate consumption; semiquantitative FFQ	Incidence of coronary heart disease (CHD)
Jackson et al., 2019b (110)	Australia	Prospective cohort studies with median follow-up 15 years	Australian Longitudinal Study on Women's Health (ALSWH).	5,324 (0%); 52 ± 2 years	Dietary nitrate intakes; FFQ	Incidence of cardiovascular disease
Jackson et al., 2020 (111)	Australia	Prospective cohort studies with mean follow-up over 15 years	Australian Longitudinal Study on Women's Health (ALSWH).	5,325 (0%); 52 ± 2 years	Adherence to the Australian Dietary Guidelines (ADG) and the Mediterranean diet (MedDiet); Dietary Questionnaire for Epidemiological Studies (DQES) FFQ	Incidence of non-fatal cardiovascular disease
Jalali et al., 2024 (112)	Iran	Prospective cohort studies with median follow-up 10.6 years	Tehran Lipid and Glucose Study (TLGS)	2,050 (46%); 46 ± 11 years	Ultra-processed food intake; 168-items semi-quantitative FFQ	Incidence of cardiovascular disease
Jeon & Park, 2019 (113)	South Korea	Prospective cohort studies with mean follow-up 7.4 years	Korean Genome and Epidemiology Study	9,142 (48%); 40–69 years	Vitamin B6 intake; 103-item semi-quantitative FFQ	Incidence of cardiovascular disease
Jeong et al., 2023 (114)	South Korea	Prospective cohort studies with median follow-up up to 5 years	Korean Genome and Epidemiology Study	13,293 (100%); 40-69 years	Meat intake; 106-item semi-quantitative FFQ	Incidence of coronary heart disease
Jo & Park, 2023 (115)	South Korea	Prospective cohort studies with median follow-up 9.59 (KARE) and 4.25 (HEXA) years	Korean Association Resource (KARE) and Health Examinee (HEXA) studies	173,696 (35%); 40 years or above	Carbohydrate-based diet; semi-quantitative FFQ	Incidence of cardiovascular disease
Johansson et al., 2019 (116)	Sweden	Prospective cohort studies with mean follow-up 14.2 years	Northern Sweden Health and Disease Study (NSHDS)	108,065 (49%); 52 ± 9 years	Dairy product Intake; FFQ	Incidence of myocardial infarction or stroke
Johansson et al., 2020 (117)	Sweden	Prospective cohort studies with median follow-up 21.8 years	Malmö Diet and Cancer Study (MDCS)	30,447 (33%); not reported	Diet quality index; modified diet history and 168-item FFQ	Incidence of carotid artery disease
Jung et al., 2023 (118)	South Korea	Prospective cohort studies with mean follow-up 11.6 years	Korean Genome and Epidemiology Study	152,828 (36%); 40 years or above	Edible mushroom intake; 103-item FFQ	Cardiovascular disease mortality
Juul et al., 2021 (119)	USA	Prospective cohort studies with mean follow-up 18 years	Framingham Offspring Cohort	3,003 (45%); 54 years	Ultra-processed food consumption; 131-item FFQ	Incidence of cardiovascular diseases and cardiovascular disease mortality
Kang et al., 2020 (120)	USA	Prospective cohort studies with mean follow-up 18.1 years	Multiethnic Cohort Study	156,434 (45%); 45-75 years	Total energy intake; >180-item quantitative FFQ	Cardiovascular disease mortality
Kashino et al., 2019 (121)	Japan	Prospective cohort studies with mean follow-up 16.2 years	Japan Public Health Center–based Prospective (JPHC) Study	92,727 (46%); 44–76 years	Dietary non-enzymatic antioxidant capacity; 147-item FFQ	Cardiovascular disease mortality
Katagiri et al., 2020a (122)	Japan	Prospective cohort studies with mean follow-up 14.8 years	Japan Public Health Center–based Prospective (JPHC) Study	92,915 (46%); 45 - 74 years	Soy and fermented soy product intake; 138-item FFQ	Cardiovascular disease, heart disease, cerebrovascular disease mortality
Katagiri et al., 2020b (123)	Japan	Prospective cohort studies with mean follow-up 16.8 years	Japan Public Health Center–based Prospective (JPHC) Study	92,924 (46%); 45 - 74 years	Dietary fiber intake; 138-item FFQ	Cardiovascular disease, heart disease, and cerebrovascular disease mortality
Kazemi et al., 2022 (124)	Iran	Prospective cohort studies with median follow-up 13.9 years	Golestan Cohort Study (GCS)	50,045 (35%); 40 years or above	Dietary approaches to stop hypertension diet score; 116-item FFQ	Cardiovascular disease mortality
Keller et al., 2020 (125)	USA	Prospective cohort studies with median follow-up 8.2 years	Harvard Pooling Project of Diet and Coronary Disease	284,345 (24%); 35 years or above	Sugar-sweetened beverages; FFQ	Incidence of coronary heart disease
Kermani-Alghoraishi et al., 2024 (126)	Iran	Prospective cohort studies with mean follow-up 11.3 years	Isfahan Cohort Study (ICS)	5,432 (49%); 51 ± 12 years	Ultra-processed food consumption; 48-item FFQ	Incidence of cardiovascular diseases and cardiovascular disease mortality
Khan et al., 2020 (127)	South Korea	Prospective cohort studies with mean follow-up 7.4 years	Korean Genome and Epidemiology Study	162,773 (34%); 40-79 years	Dietary inflammatory index; semi-quantitative FFQ	Incidence of cardiovascular disease
Kim et al., 2021 (128)	South Korea	Prospective cohort studies with mean follow-up 10 years	Korean Genome and Epidemiology Study	118,577 (35%); 40-69 years	Plant-based diet; FFQ	Cardiovascular disease mortality
Kityo & Lee, 2023 (129)	South Korea	Prospective cohort studies with median follow-up 10.6 years	Korean Genome and Epidemiology Study	113,576 (34%); 40-69 years	Intake of ultra-processed foods; 106-item FFQ	Cardiovascular disease mortality
Kjeldsen et al., 2022 (130)	Denmark	Prospective cohort studies with median follow-up 10 years	Copenhagen General Population Study	94,321 (45%); 46-70 years	Adherence to Danish dietary guidelines; FFQ	Incidence of atherosclerotic cardiovascular disease

Author, year	Country	Study design and mean/median follow-up period	Cohort used	No. of participants (% of male) and age at baseline	Dietary exposure and assessment tool	CVD outcomes
Kouvari et al., 2020a (131)	Greece	Prospective cohort studies with mean follow-up 8.4 years	ATTICA study	3,042 (50%); 46 ± 14 years	Dairy products; FFQ	Incidence of cardiovascular disease
Kouvari et al., 2020b (132)	Greece	Prospective cohort studies with mean follow-up 10 years	ATTICA study	1,885 (54%); >18 years	Vitamin D intake; 163-item semi-quantitative FFQ	Development of a fatal or non-fatal CVD event
Kouvari et al., 2022 (133)	Greece	Prospective cohort studies with median follow-up 8.4 years	ATTICA study	2,020 (50%); 18 years or above	Plant-based diets; semi-quantitative FFQ	Incidence of cardiovascular disease
Kvist et al., 2020 (134)	Denmark	Prospective cohort studies with median follow-up 15.9 years	Danish Diet, Cancer, and Health cohort	54,903 (47%); 50-64 years	Replacement of whole-fat dairy products with low-fat variants; 192-item semi-quantitative FFQ	Incidence of myocardial infarction
Kwon et al., 2022 (135)	South Korea	Prospective cohort studies with mean follow-up 10.1 years	Korean Genome and Epidemiology Study	143,050 (36%); 54 ± 9 years	Dietary fiber intake: FFQ	Cardiovascular disease mortality
Kwon et al., 2023 (136)	South Korea	Prospective cohort studies with mean follow-up 10.1 years	Korean Genome and Epidemiology Study	143,050 (37%); 40 years and older	Zinc intake; 103-item semi-quantitative FFQ	Cardiovascular disease mortality
Langsetmo et al., 2020 (137)	USA	Prospective cohort studies with mean follow-up <10 years	Osteoporotic Fractures in Men (MrOS) study	5,790 (100%); 65 years or above	Total energy and protein intake; 69-item FFQ	Cardiovascular disease mortality
Lara et al., 2019 (138)	USA	Prospective cohort studies with median follow-up 8.7 years	REasons for Geographic and Racial Differences in Stroke (REGARDS) study	16,068 (41%); 64 ± 9 years	Five dietary patterns (convenience, plant-based, sweets, Southern, and alcohol/salads); 107-item FFQ	Incidence of heart failure
Laursen et al., 2019 (139)	Denmark	Prospective cohort studies with median follow-up 15.2 years	European Prospective Investigation into Cancer and Nutrition-Netherlands cohort (EPIC-NL)	36,886 (25%); 49-70 years	Intake of different dairy products; 178-item FFQ	Incidence of stroke
Lee et al., 2019 (140)	USA	Prospective cohort studies with mean follow-up 26 years	Nurses' Health Study (NHS) and Health Professionals Follow-up Study (HPFS)	110,680 (39%); 30-75 years	Mushroom consumption; FFQ	Incidence of coronary heart disease and stroke
Li et al., 2020a (141)	USA	Prospective cohort studies with mean follow-up up to 32 years	Nurses' health study (NHS), NHSII, and Health Professionals Follow-up Study (HPFS)	210,145 (21%); 25 - 75 years	Dietary inflammatory potential; FFQ	Incidence of cardiovascular disease, coronary heart disease, stroke, and cardiovascular disease mortality
Li et al., 2020b (142)	USA	Prospective cohort studies with mean follow-up 10.5 years	Women's Health Initiative (WHI)	153,569 (0%); 63 years	Dietary magnesium; 122-item semiquantitative FFQ	Incidence of fatal coronary heart disease and sudden cardiac death
Li et al., 2021 (143)	USA	Prospective cohort studies with mean follow-up 22 years	Iowa Women's Health Study (IWHS)	33,155 (0%); 55–69 years	Dietary inflammation score; 127-item Willett FFQ	Cardiovascular disease mortality
Li et al., 2023a (144)	China	Prospective cohort studies with median follow-up 11.9 years	Prospective Urban and Rural Epidemiological (PURE) China study	47,931 (42%); 51 ± 10 years	Dietary copper intake: FFQ	Incidence of cardiovascular diseases and cardiovascular disease mortality
Li et al., 2023b (145)	China	Prospective cohort studies with median follow-up 8.5 years	China Kadoorie Biobank study	26,163 (39%); 30-79 years	Spicy food consumption; qualitative FFQ	Incidence of ischemic heart disease, stroke and cardiovascular mortality
Liang et al., 2022 (146)	USA	Prospective cohort studies with median follow-up 17 years	Prostate, Lung, Colorectal, and Ovarian (PLCO) Cancer Screening Trial	101,832 (not reported); 62 ± 5 years	Dietary inflammatory Index; DHQ	Cardiovascular disease mortality
Lilja et al., 2019 (147)	Sweden	Prospective cohort studies with median follow-up 19.7 years	Malmö Diet and Cancer Study (MDCS)	1,112 (51%); 45-64 years	Intake of fish and shellfish; 168-item FFQ	Incidence of symptomatic peripheral arterial disease
Lim et al., 2022 (148)	Singapore	Prospective cohort studies with mean follow-up 10.1 years	Singapore Multi-Ethnic Cohort	12,408 (43%); 21-65 years	Replacing dietary carbohydrates and refined grains; 169-item FFQ	Incidence of nonfatal acute myocardial infarction, nonfatal stroke and cardiovascular disease mortality
Liu et al., 2019a (149)	USA	Prospective cohort studies with mean follow-up 26 years	Nurses' Health Study (NHS) and Health Professionals Follow-up Study (HPFS)	16,217 (not reported); 30-75 years	Nut consumption; 131-item semi-quantitative FFQ	Incidence of coronary heart disease and stroke, cardiovascular disease mortality
Liu et al., 2019b (150)	Australia	Prospective cohort studies with mean follow-up 14 years	Blue Mountains Eye Study	2,229 (41%); 49 years or above	Dietary nitrate intake from vegetables; 145-item semi-quantitative FFQ	Cardiovascular disease mortality
Liu et al., 2020 (151)	USA	Prospective cohort studies with mean follow-up up to 26 years	Nurses' health study (NHS), NHSII, and Health Professionals Follow-up Study (HPFS)	192,655 (18%); 24-75 years	Nut consumption; ~130-item FFQ	Incidence of cardiovascular disease, coronary heart disease, and stroke
Liu et al., 2021 (152)	China	Prospective cohort studies with mean follow-up 8.9 years	Prospective Urban and Rural Epidemiological (PURE) study	41,243 (42%); 42-80 years	Fruit, vegetable and legume consumption; FFQ	Cardiovascular disease mortality
Liu et al., 2023 (153)	China	Prospective cohort studies with median follow-up 9.2 years	Guangdong Coronary Artery Disease Cohort	1,292 (68%); 64 ± 12 years	Dietary betaine intake; FFQ	Cardiovascular disease mortality
Liu et al., 2024a (154)	China	Prospective cohort studies with mean follow-up 3.8 years	Prospective cohort study in Chongqing	22,661 (47%); 51 ± 12 years	Alcohol, red meat, fruits and vegetables intake; 12-item FFQ	Total stroke and ischemic stroke
Liu et al., 2024b (155)	China	Prospective cohort studies with median follow-up 5.5 years	PFS-CMMC	15,518 (32%); 35-74 years	Trimethylamine N-oxide, b-alanine, tryptophan index, and vitamin B6-related dietary patterns; semi-quantitative FFQ	Incidence of total stroke and non-ischemic stroke
Livingstone et al., 2022 (156)	Australia	Prospective cohort studies with mean follow-up 17.7 years	Australian Diabetes, Obesity, and Lifestyle study	10,009 (48%); 52 ± 14 years	Australian Dietary Guideline Index, Dietary Inflammatory Index, Mediterranean-Dietary Approaches to Stop Hypertension Intervention for Neurodegenerative Delay; 74-item semi-quantitative FFQ	Cardiovascular disease mortality
Lo et al., 2021 (157)	China	Prospective cohort studies with median follow-up 11.1 years	Mr. OS and Ms. OS study	3,991 (50%); 68-76 years	Portfolio diet; 280-item semi-quantitative FFQ	Cardiovascular disease mortality
Lukic et al., 2020 (158)	Norway	Prospective cohort studies with median follow-up 20.5 years	Norwegian Women and Cancer Study (NOWAC)	117,228 (0%); 47 ± 9 years	Coffee consumption; semiquantitative FFQ	Cardiovascular disease mortality
Luong et al., 2023 (159)	Australia	Prospective cohort studies with median follow-up 5.3 years	Concord Health and Ageing in Men Project (CHAMP)	539 (100%); 77-83 years	Dietary iron intake; dietitian-administered diet history questionnaire	Myocardial infarction, congestive cardiac failure, coronary revascularization, and/or ischemic stroke
Luong et al., 2024 (160)	Australia	Prospective cohort studies with mean follow-up 5.3 years	Concord Health and Ageing in Men Project (CHAMP)	539 (100%); 75 years or older	Empirically derived dietary patterns; dietitian-administered diet history questionnaire	Myocardial infarction, congestive cardiac failure, coronary revascularization, and/or ischemic stroke
Ma et al., 2020 (161)	USA	Prospective cohort studies with mean follow-up 22.9 years	Nurses' health study (NHS), NHSII, and Health Professionals Follow-up Study (HPFS)	210,700 (20%); 25–75 years	Isoflavone Intake; 130-item FFQ	Incidence of coronary heart disease
Ma et al., 2023 (162)	USA	Prospective cohort studies with mean follow-up 18.5 years	Nurses' Health Study (NHS) and Health Professionals Follow-up Study (HPFS)	15,486 (26%); 61 years	Beverage consumption; FFQ	Incidence of cardiovascular disease and mortality
Malik et al., 2019 (163)	USA	Prospective cohort studies with median follow-up 28.9 years	Nurses' Health Study (NHS) and Health Professionals Follow-up Study (HPFS)	118,363 (32%); 30-75 years	Sugar sweetened and artificially sweetened beverages; 131- to 166-item FFQ	Cardiovascular disease mortality
Mao et al., 2022 (164)	USA	Prospective cohort studies with median follow-up 11 years	REasons for Geographic and Racial Differences in Stroke (REGARDS) study	15,467 (41%); 45 years or above	13-component evolutionary-concordance diet score; 110-item Block 98 FFQ	Incidence of cardiovascular disease, coronary heart disease, and stroke
Matre et al., 2021 (165)	Norway	Prospective cohort studies with median follow-up 14.1 years	Western Norway B-Vitamin Intervention Trial	1,929 (80%); 18 years or above	Meat intake; 169-item semi-quantitative FFQ	Incidence of acute myocardial infarction and cardiovascular mortality

Author, year	Country	Study design and mean/median follow-up period	Cohort used	No. of participants (% of male) and age at baseline	Dietary exposure and assessment tool	CVD outcomes
Matsuyama et al., 2021 (166)	Japan	Prospective cohort studies with median follow-up 18.9 years	Japan Public Health Center–based Prospective (JPHC) Study	92,969 (46%); 45–74 years	Japanese diet index;147-item FFQ	Cardiovascular disease mortality
Mendonça et al., 2019 (167)	Spain	Prospective cohort studies with mean follow-up 10.1 years	Seguimiento University of Navarra (SUN) project	17,065 (39%); 20-89 years	Total polyphenol intake; 136-item FFQ	Incidence of cardiovascular disease
Meng et al., 2023a (168)	China	Prospective cohort studies with median follow-up 3.2 years	Tianjin Chronic Low-grade Systemic Inflammation and Health (TCLSIH) cohort study	3,828 (52%); 18 years or above	Soft drink consumption; 100-item FFQ	Incidence of carotid atherosclerosis
Meng et al., 2023b (169)	China	Prospective cohort studies with median follow-up 4.2 years	Tianjin Chronic Low-grade Systemic Inflammation and Health (TCLSIH) cohort study	2,166 (55%); 19 - 85 years	Whole-grain: 100-item FFQ	Incidence of carotid atherosclerosis
Michaëlsson et al., 2020 (170)	Sweden	Prospective cohort studies with mean follow-up 17.4 years	Swedish Mammography Cohort (SMC) and Cohort of Swedish Men (COSM)	79,003 (56%); 61 years	Modified mediterranean-like diet (mMED) score; FFQ	Cardiovascular disease mortality
Mirmiran et al., 2020 (171)	Iran	Prospective cohort studies with median follow-up 6.7 years	Tehran Lipid and Glucose Study (TLGS)	2,369 (44%); 19–70 years	Dietary fatty acids; 168-item FFQ	Incidence of cardiovascular diseases, coronary heart disease, stroke, or cardiovascular disease mortality
Mirmiran et al., 2023 (172)	Iran	Prospective cohort studies with median follow-up 10.6 years	Tehran Lipid and Glucose Study (TLGS)	2,918 (45%); 39 ± 14 years	Modified Nordic diet; 168-item FFQ	Incidence of cardiovascular disease, myocardial infarction, unstable angina, angiography-proven coronary heart disease (CHD), heart failure, or death from CHD, a stroke, or a temporary ischemic event)
Mohammadifard et al., 2021 (173)	Iran	Prospective cohort studies with median follow-up 11.3 years	Isfahan Cohort Study (ICS)	5,432 (49%); 35 years or above	Nut consumption; 48-item FFQ	Cardiovascular disease mortality
Mohammadifard et al., 2022 (174)	Iran	Prospective cohort studies with mean follow-up 11.3 years	Isfahan Cohort Study (ICS)	4,367 (50%); 35 years or above	Egg consumption; 48-item FFQ	Incidence of myocardial infarction, ischemic coronary heart disease, stroke, cardiovascular disease and CVD mortality
Mohan et al., 2021 (175)	58 Countries	Prospective cohort studies with median follow-up 9.1 years	Perspective Urban and Rural Epidemiology (PURE) study	191,558 (48%); 54 ± 8 years	Fish consumption; country-specific FFQ	Incidence of cardiovascular diseases and cardiovascular disease mortality
Mohseni et al., 2023 (176)	Iran	Prospective cohort studies with median follow-up 11.3 years	Isfahan Cohort Study (ICS)	5,432 (49%); 35 years or above	Hydrogenated vegetable oil, non-hydrogenated vegetable oil, red meat, fish, fast foods, high fat dairy products, fast foods, sweets and sof drinks; 48-item FFQ	Incidence of myocardial infarction, stroke, cardiovascular disease and cardiovascular mortality
Mori et al., 2019 (177)	Japan	Prospective cohort studies with median follow-up 16.9 years	Japan Public Health Center–based Prospective (JPHC) Study	88,184 (46%); 45-74 years	Cruciferous vegetable intake; 138-item FFQ	Heart disease and cerebrovascular disease mortality
Mosallanezhad et al., 2023 (178)	Iran	Prospective cohort studies with mean follow-up 10.6 years	Tehran Lipid and Glucose Study (TLGS)	2,050 (46%); 30–84 years	Sodium and potassium intake; 168-item FFQ	Coronary heart disease, stroke and cardiovascular disease mortality
Murai et al., 2019 (179)	Japan	Prospective cohort studies with mean follow-up 17.3 years	Japan Public Health Center–based Prospective (JPHC) Study	86,113 (47%); 40-69 years	Seaweed intake; 44- to 52-item FFQ	Incidences of stroke and ischemic heart disease
Musicus et al., 2022 (180)	USA	Prospective cohort studies with mean follow-up 14.7 years	Nurses' Health Study II	156,509 (0%); 25-42 years	Healthy dietary index and three plant-based indices by their environmental impacts; 156-item FFQ	Incidence of cardiovascular disease
Nanri et al., 2023 (181)	Japan	Prospective cohort studies with mean follow-up 18.9 years	Japan Public Health Center–based Prospective (JPHC) Study	93,685 (46%); 40-69 years	Vitamin D intake; 147-item FFQ	Cardiovascular disease mortality
Norouzzadeh et al., 2024 (182)	Iran	Prospective cohort studies with mean follow-up 9 years	Tehran Lipid and Glucose Study (TLGS)	5,048 (45%); 46.42 years	Dietary quality indices; 168-item FFQ	Incidence of stroke, cardiovascular diseases and cardiovascular disease mortality
Nozue et al., 2021 (183)	Japan	Prospective cohort studies with mean follow-up 13.3 years	Japan Public Health Center–based Prospective (JPHC) Study	79,648 (46%); 45–74 years	Fermented soy products intake; 138-item FFQ	Incidence of cardiovascular disease
Oh et al., 2022 (184)	USA	Prospective cohort studies with median follow-up 15.9 years	Multi-Ethnic Study of Atherosclerosis	6,109 (48%); 45-84 years	Low-carbohydrate diets; 120-item FFQ	Cardiovascular disease mortality
Okada et al., 2019 (185)	Japan	Prospective cohort studies with median follow-up 19.3 years	Japan Collaborative Cohort Study (JACC)	58,782 (39%); 40 - 79 years	Dietary inflammatory index; FFQ	Cardiovascular disease, coronary heart disease, and stroke mortality
Pacheco et al., 2020 (186)	USA	Prospective cohort studies with mean follow-up 20 years	California Teachers Study	106,178 (0%); 52 ± 13 years	Sugar-sweetened beverage consumption; 103-item FFQ	Incidence of cardiovascular disease
Pacheco et al., 2022a (187)	USA	Prospective cohort studies with mean follow-up 20 years	California Teachers Study	100,314 (0%); 53 years	Sugar-sweetened beverage consumption; 103-item FFQ	Cardiovascular disease mortality
Pacheco et al., 2022b (188)	USA	Prospective cohort studies with mean follow-up up to 30 years	Nurses' Health Study (NHS) and Health Professionals Follow-up Study (HPFS)	110,487 (38%); 30-75 years	Avocado consumption; 130-item FFQ	Incidence of total cardiovascular disease, total coronary heart disease, and total stroke
Palmer et al., 2021 (189)	Denmark	Prospective cohort studies with median follow-up 21 years	Danish Diet, Cancer, and Health cohort	56,048 (48%); 52 - 60 years	Vitamin K1 intake; 192-item semiquantitative FFQ	Cardiovascular disease mortality
Pan et al., 2021 (190)	China and USA	Prospective cohort studies with median follow-up 12 years (SCCS) and 15.5 years (SWHS/SMHS)	Southern Community Cohort Study (SCCS), Shanghai Men's Health Study (SMHS), and Shanghai Women's Health Study (SWHS)	202,429 (44%); 40 - 79 years	Cholesterol and egg intakes; FFQ	Cardiovascular disease, coronary heart disease, and stroke mortality
Panagiotakos et al., 2019 (191)	Greece	Prospective cohort studies with mean follow-up 8.4 years	ATTICA study	2,020 (not reported); 18 - 89 years	Beer and wine consumption; EPIC-Greek semiquantitative FFQ	Incidence of cardiovascular disease
Papandreou et al., 2019 (192)	Spain	Prospective cohort studies with median follow-up 6 years	PREvencion con DIeta MEDiterranea (PREDIMED) study	7,212 (43%); 67 ± 6 years	Total legumes consumption and grain legumes species (dry beans, chickpeas, lentils, and fresh peas); 137-item semi-quantitative FFQ	Cardiovascular disease mortality
Parmenter et al., 2021 (193)	Denmark	Prospective cohort studies with mean follow-up 21 years	Danish Diet, Cancer, and Health cohort	55,169 (47%); 52-60 years	Habitual flavonoid consumption; 192-item FFQ	Incidence of ischemic stroke
Parmenter et al., 2023a (194)	Denmark	Prospective cohort studies with mean follow-up 12.8 to 14.2 years	Danish Diet, Cancer, and Health cohort	55,094 (47%); 52-60 years	Habitual dietary intakes of flavanols and anthocyanins; 192-item FFQ	Incidence of ischemic stroke subtypes
Parmenter et al., 2023b (195)	Denmark	Prospective cohort studies with mean follow-up 18.5 to 21.7 years	Danish Diet, Cancer, and Health cohort	54,496 (47%); 52-60 years	Intake of dietary flavonoids; 192-item FFQ	Incidence of ischemic heart disease
Patel et al., 2021 (196)	USA	Prospective cohort studies with mean follow-up 9.8 years	Physicians' Health Study	15,768 (100%); 66 ± 9 years	Mediterranean, the Dietary Approaches to Stop Hypertension and the Alternate Healthy Eating Index diet; semi-quantitative FFQ	Cardiovascular disease mortality
Pertiwi et al., 2021 (197)	The Netherlands	Prospective cohort studies with median follow-up 12 years	Alpha Omega Cohort (AOC)	4,067 (79%); 60 - 80 years	Dietary long-chain omega-3 polyunsaturated fatty acids intake; 203-item FFQ	Cardiovascular disease and coronary heart disease mortality
Praagman et al., 2019 (198)	UK and Denmark	Prospective cohort studies with median follow-up 18.8 (UK) and 13.6 (Denmark) years	The European Investigation into Cancer and Nutrition-Norfolk (EPIC-Norfolk) cohort) and the Danish Diet, Cancer and Health cohort (EPIC-Denmark)	75,425 (45%); 40 - 74 years	Individual saturated fatty acids; country-specific FFQ	Incidence of myocardial infarction

Author, year	Country	Study design and mean/median follow-up period	Cohort used	No. of participants (% of male) and age at baseline	Dietary exposure and assessment tool	CVD outcomes
Ruggiero et al., 2021a (199)	Italy	Prospective cohort studies with median follow-up 8.3 years	Moli-sani Study	20,487 (47%); 35–94 years	Daily coffee drinking; 188-item FFQ	Cardiovascular mortality and ischemic heart disease or cerebrovascular disease mortality
Ruggiero et al., 2021b (200)	Italy	Prospective cohort studies with mean follow-up 8.2 years	Moli-sani Study	20,562 (47%); 35-94 years	Egg consumption; 188-item semi-quantitative FFQ	Cardiovascular disease mortality
Ruggiero et al., 2024 (201)	Italy	Prospective cohort studies with mean follow-up 13.1 years	Moli-sani Study	22,892 (48%); 55 ± 12 years	Olive oil consumption; 188-item FFQ	Cardiovascular disease mortality
Sadeghi et al., 2021 (202)	Iran	Prospective cohort studies with median follow-up 11.3 years	Isfahan Cohort Study (ICS)	5,432 (49%); 35 years or above	Dietary fat intake; Persian FFQ	Incidence of ischemic heart disease, stroke and cardiovascular mortality
Saglimbene et al., 2019a (203)	Europe and South America	Prospective cohort studies with median follow-up up to 2.7 years	Dietary Intake, Death and Hospitalization in Adults with End-Stage Kidney Disease Treated with Hemodialysis (DIET-HD) study	8,110 (58%); 63 ± 15 years	Dietary n-3 polyunsaturated fatty acid intake; Global Allergy and Asthma European Network (GA2LEN) FFQ	Cardiovascular disease mortality
Saglimbene et al., 2019b (204)	Europe	Prospective cohort studies with mean follow-up 2.7 years	Dietary Intake, Death and Hospitalization in Adults with End-Stage Kidney Disease Treated with Hemodialysis (DIET-HD) study	8,078 (58%); 63 ± 15 years	Fruit and vegetable intake; Global Allergy and Asthma European Network (GA2LEN) FFQ	Cardiovascular disease mortality
Saglimbene et al., 2020 (205)	Europe and South America	Prospective cohort studies with median follow-up 2.7 years	Dietary Intake, Death and Hospitalization in Adults with End-Stage Kidney Disease Treated with Hemodialysis (DIET-HD) study	8,110 (58%); 63 ± 15 years	Fruit and vegetable dietary pattern and western dietary pattern; Global Allergy and Asthma European Network (GA2LEN) FFQ	Cardiovascular disease mortality
Sahashi et al., 2022 (206)	Japan	Prospective cohort studies with median follow-up 20.9 years	Japan Public Health Center–based Prospective (JPHC) Study	94,658 (46%); 40–69 years	Fruit and vegetable intake; 147-item FFQ	Cardiovascular disease mortality
Saito et al., 2020 (207)	Japan	Prospective cohort studies with mean follow-up 14 years	Japan Public Health Center–based Prospective (JPHC) Study	87,507 (46%); 45 - 74 years	Meat intake; 138-item semiquantitative FFQ	Heart disease mortality
Sakamaki et al., 2021 (208)	Japan	Prospective cohort studies with mean follow-up 18.4 years	Jichi Medical School Cohort Study	9,946 (39%); 19 - 93 years	Coffee consumption; semiquantitative FFQ	Coronary heart disease and stroke mortality
Sawicki et al., 2024 (209)	USA	Prospective cohort studies with mean follow-up 23.5 years	Nurses' health study (NHS), NHSII, and Health Professionals Follow-up Study (HPFS)	193,618 (22%); 25-75 years	Planetary health diet; >110-item semiquantitative FFQ	Incidence of cardiovascular disease
Scheffers et al., 2019 (210)	Netherlands	Prospective cohort studies with mean follow-up 14.6 years	European Prospective Investigation into Cancer and Nutrition-Netherlands cohort (EPIC-NL)	34,560 (26%); 20–69 years	Pure fruit juice and fruit consumption; 178-item FFQ	Incidence of cardiovascular disease, coronary heart disease and stroke
Schmidt et al., 2020 (211)	Denmark	Prospective cohort studies (follow-up period not reported)	Danish National Birth Cohort	66,387 (0%); not reported	Dietary glycemic index and glycemic load sugar-sweetened beverages; FFQ	Incidence of congenital heart disease
Shams-White et al., 2022 (212)	USA	Prospective cohort studies with mean follow-up 14.2 years	National Institutes of Health-AARP (NIH-AARP) Diet and Health Study	177,410 (54%); 50–71 years	2018 World Cancer Research Fund/American Institute for Cancer Research (WCRF/AICR) Score; 124-item FFQ	Cardiovascular disease mortality
Shan et al., 2020 (213)	USA	Prospective cohort studies with mean follow-up up to 32 years	Nurses' health study (NHS), NHSII, and Health Professionals Follow-up Study (HPFS)	165,794 (26%); 36 - 53 years	Healthy Eating Index–2015 (HEI-2015), Alternate Mediterranean Diet Score (AMED), Healthful Plant-Based Diet Index (HPDI), and Alternate Healthy Eating Index (AHEI); FFQ	Incidence of cardiovascular disease, coronary heart disease, and stroke
Shao et al., 2022 (214)	China	Prospective cohort studies with mean follow-up 11.4 years	Guangzhou Biobank Cohort Study	18,215 (29%); 63 ± 7 years	Fish intake; FFQ	Cardiovascular disease, ischemic heart disease, or stroke mortality
Sheng et al., 2022 (215)	Singapore	Prospective cohort studies with mean follow-up 19.5 years	Singapore Chinese Health Study	62,063 (44%); 45-74 years	Dietary total antioxidant capacity; 165-item structured quantitative FFQ	Cardiovascular disease mortality
Shikany et al., 2021 (216)	USA	Prospective cohort studies with mean follow-up 9.8 years	REasons for Geographic and Racial Differences in Stroke (REGARDS) study	21,069 (44%); 45 years or above	Mediterranean diet score; 110-item semi-quantitative FFQ	Incidence of sudden cardiac death
Shin et al., 2022 (217)	China, Japan, South Korea, and Singapore	Prospective cohort studies with mean follow-up 6.5 - 22.7 years	Asia Cohort Consortium (12 prospective cohorts)	528,504 (47%); 54 ± 10 years	Coffee and tea consumption; FFQ and DHQ	Cardiovascular disease mortality
Silva et al., 2022 (218)	Brazil	Prospective cohort studies with mean follow-up 8.1 years	Brazilian Longitudinal Study of Adult Health (ELSA-Brazil)	6,671 (50%); 52 ± 9 years	Dairy product consumption; 114-item semiquantitative FFQ	Cardiovascular disease mortality
Sjöblom et al., 2024 (219)	Sweden	Prospective cohort studies with mean follow-up 17.9 years	Swedish National March Cohort (SNMC)	34,898 (34%); 49 ± 16 years	Adherence to the Nordic Nutrition Recommendations; 85-item semi-quantitative FFQ	Incidence of myocardial infarction and stroke
Son et al., 2024 (220)	South Korea	Prospective cohort studies with mean follow-up 10.1 years	Korean Genome and Epidemiology Study	143,050 (36%); 40 years or above	Carbohydrate to protein or fat ratio; Semiquantitative FFQ	Cardiovascular disease mortality
Son et al., 2019 (221)	South Korea	Prospective cohort studies with mean follow-up 10 years	Ansan–Ansung cohort study	8,618 (48%); 40–69 years	Processed red meat consumption; 103-item semi-quantitative FFQ	Incidence of cardiovascular disease
Stefler et al., 2021 (222)	Russia, Poland and the Czech Republic	Prospective cohort studies with mean follow-up 11 years	HAPIEE study	18,852 (not reported); 45-70 years	Eastern European diet score; semi-quantitative FFQ	Cardiovascular disease mortality
Strengers et al., 2021 (223)	Netherlands	Prospective cohort studies with median follow-up 15 years	European Prospective Investigation into Cancer and Nutrition-Netherlands cohort (EPIC-NL)	36,961 (25%); 49-70 years	Adherence to a Mediterranean-style diet; semi-quantitative FFQ	Incidence of heart failure
Su et al., 2023 (224)	China	Prospective cohort studies with median follow-up 10.1 years	China Kadoorie Biobank study	51,929 (42%); 52 years	Health dietary pattern score; Qualitative FFQ	Incidence of cardiovascular disease, ischemic heart disease, and ischemic stroke
Sun et al., 2019 (225)	USA	Prospective cohort studies with mean follow-up 17.9 years	Women's Health Initiative (WHI)	15,166 (0%); 65 ± 7 years	Fried food consumption; 122-item FFQ	Cardiovascular disease mortality
Sun et al., 2023 (226)	China	Prospective cohort studies with mean follow-up 14.6 years	Guangzhou Biobank Cohort Study	19,597 (28%); 50 years or above	Fruit and vegetable consumption; 300-item FFQ	Cardiovascular disease mortality
Sun et al., 2024 (227)	China	Prospective cohort studies with mean follow-up 15.8 years	Guangzhou Biobank Cohort Study	19,598 (not reported); >50 years	Cantonese dietary patterns; 300-item FFQ	Cardiovascular disease mortality
Swaminathan et al., 2021 (228)	21 countries	Prospective cohort studies with median follow-up 9.5 years	Prospective Urban and Rural Epidemiological (PURE) study	137,130 (42%); 50 ± 10 years	Cereal grains intake; country-specific FFQ	Cardiovascular disease mortality
Taguchi et al., 2020 (229)	Japan	Prospective cohort studies with mean follow-up 14.1 years	Takayama Study	29,079 (46%); 35 years or above	Dietary intake of total polyphenols; 169-item semi-quantitative FFQ	Cardiovascular disease mortality, stroke mortality, and ischemic heart disease mortality
Talaei et al., 2019a (230)	Singapore	Prospective cohort studies with mean follow-up 17.2 years	Singapore Chinese Health Study	57,078 (46%); 45 - 74 years	Dietary approaches to stop hypertension (DASH) diet; 165-item FFQ	Stroke and coronary artery disease mortality
Talaei et al., 2019b (231)	Iran	Prospective cohort studies with median follow-up 10.9 years	Isfahan Cohort Study (ICS)	5,432 (49%); 35 years or above	Whole milk consumption; 48-item FFQ	Incidence of cardiovascular diseases and cardiovascular disease mortality

Author, year	Country	Study design and mean/median follow-up period	Cohort used	No. of participants (% of male) and age at baseline	Dietary exposure and assessment tool	CVD outcomes
Tamura et al., 2023 (232)	Japan	Prospective cohort studies with mean follow-up 8.9 years	Japan Multi-Institutional Collaborative Cohort (J-MICC) study	81,333 (43%); 35 - 69 years	Dietary carbohydrate and fat intakes; 46-item FFQ	Cardiovascular disease mortality
Tang et al., 2023 (233)	Japan	Prospective cohort studies with median follow-up 19 years	Japan Collaborative Cohort Study (JACC)	85,319 (42%); 40-79 years	Breakfast type; FFQ	Cardiovascular disease, stroke, hemorrhagic stroke, and cerebral infarction mortality
Tanno et al., 2021 (234)	Japan	Prospective cohort studies with mean follow-up 10.7 years	Iwate-Kenpoku cohort (Iwate-KENCO) study	14,121 (30%); 40 - 69 years	Milk intake; 58-item brief-type self-administered diet history questionnaire (BDHQ)	Incident stroke
Tong et al., 2023 (235)	Seven countries (Denmark, Germany, Italy, the Netherlands, Spain, Sweden, and the UK),)	Prospective cohort studies with median follow-up 112.9 years	European Prospective Investigation into Cancer and Nutrition cohort (EPIC)	356,142 (36%); 35 - 70 years	Dietary amino acids; country-specific FFQ/diet records	Incidence of hemorrhagic stroke and ischemic stroke
Torres-Collado et al., 2021 (236)	Spain	Prospective cohort studies with median follow-up 18 years	Valencia Nutrition Study (VNS)	1,567 (46%); 46 ± 18 years	Coffee consumption; semiquantitative FFQ	Cardiovascular disease mortality
Torres-Collado et al., 2022 (237)	Spain	Prospective cohort studies with median follow-up 18 years	Valencia Nutrition Study (VNS)	1,540 (47%); 20 - 97 years	Dietary diversity score; 93-item FFQ	Cardiovascular disease mortality
Troeschel et al., 2021 (238)	USA	Prospective cohort studies with median follow-up 10.3 years	REasons for Geographic and Racial Differences in Stroke (REGARDS) study	17,465 (43%); > 45 years	13-component evolutionary-concordance diet score; 110-item Block 98 FFQ	Cardiovascular disease mortality
Troeschel et al., 2023 (239)	USA	Prospective cohort studies with median follow-up 10.3 years	REasons for Geographic and Racial Differences in Stroke (REGARDS) study	18,484 (43%); 64 ± 9 years	19-component dietary inflammation score (DIS); Block 98 FFQ	Cardiovascular disease mortality
Um et al., 2019 (240)	USA	Prospective cohort studies with mean follow-up 26 years	Iowa Women's Health Study (IWHS)	35,221 (0%); 55 - 69 years	Calcium and dairy product intakes; 127-item Willett FFQ	Coronary heart disease mortality
Ushula et al., 2023 (241)	Australia	Prospective cohort studies with mean follow-up 30 years	Mater-University of Queensland Study of Pregnancy (MUSP) birth cohort study	875 (35%); 30 ± 1 years	Dietary pattern score; 74-item semiquantitative FFQ	Incidence of cardiovascular events
Van Parys et al., 2020 (242)	Norway	Prospective cohort studies with median follow-up 7.5 years	Western Norway B-Vitamin Intervention Trial	1,981 (80%); 62 years	Dietary choline; 169-item FFQ	Incidence of acute myocardial infarction
Vanegas et al., 2022 (243)	Spain	Prospective cohort studies with mean follow-up 14 years	Seguimiento University of Navarra (SUN) project	18,418 (39%); 36 ± 12 years	Macronutrient quality index; 136-item FFQ	Incidence of cardiovascular disease
Vázquez-Ruiz et al., 2022 (244)	Spain	Prospective cohort studies with mean follow-up 11.5 years	Seguimiento University of Navarra (SUN) project	16,147 (39%); 37 ± 12 years	Dietary phenolic compounds intake; 130-item semi-quantitative FFQ	Incidence of cardiovascular disease
Venø et al., 2019 (245)	Denmark	Prospective cohort studies with mean follow-up 13.5 years	Danish Diet, Cancer, and Health cohort	55,338 (48%); 50-65 years	Total marine n-3 polyunsaturated fatty acids; 192-item semiquantitative FFQ	Incidence of ischemic stroke
Veronese et al., 2020 (246)	Italy	Prospective cohort studies with median follow-up 12 years	Multicentrica Italiana COLeleitiasi (MICOL) III	1,565 (55%); 66 years	Dietary inflammatory index; 70-item FFQ	Cardiovascular disease mortality
Vissers et al., 2019 (247)	Dutch	Prospective cohort studies with mean follow-up 15 years	European Prospective Investigation into Cancer and Nutrition-Netherlands cohort (EPIC-NL)	35,767 (25%); 20-70 years	Fatty acids from dairy and meat; 178-item FFQ	Incidence of coronary heart disease
Vogtschmidt et al., 2024 (248)	UK	Prospective cohort studies with median follow-up 21 years	European Prospective Investigation into Cancer and Nutrition-Norfolk study	21,841 (44%); 40-79 years	Replacement of saturated fatty acids from total meat by total dairy; 130-item semiquantitative FFQ	Incidence of fatal or nonfatal cardiovascular disease, coronary artery disease and stroke
Voortman et al., 2021 (249)	The Netherlands	Prospective cohort studies with mean follow-up 12.7 years	Rotterdam study	5,783 (39%); 62 ± 8 years	Macronutrient intake: FFQ	Incidence of coronary heart disease
Wada et al., 2022 (250)	Japan	Prospective cohort studies with mean follow-up 14.1 years	Takayama Study	29,079 (46%); 35 years or above	Rice-Based diet; 169-item FFQ	Cardiovascular disease mortality
Wan et al., 2022 (251)	USA	Prospective cohort studies with mean follow-up up to 30 years	Nurses' Health Study (NHS) and Health Professionals Follow-up Study (HPFS)	106,344 (40%); 30-75 years	Dietary insulinemic potential; >130-item FFQ	Cardiovascular disease mortality
Wang et al., 2021a (252)	China	Prospective cohort studies with median follow-up 7.8 years	China Kadoorie Biobank study	487,034 (40%); 52 ± 11 years	Consumption of soy products; FFQ	Cardiovascular disease mortality
Wang et al., 2021b (253)	China	Prospective cohort studies with mean follow-up 7.8 years	China Kadoorie Biobank study	26,139 (39%); 30-79 years	Soy food consumption; FFQ with 12 food groups	Cardiovascular disease mortality, including death from chronic heart disease, acute myocardial infarction, stroke, ischemic stroke and hemorrhagic stroke
Wang et al., 2022a (254)	USA	Prospective cohort studies with mean follow-up 3.5 years	Million Veteran Program (MVP)	180,156 (not reported); 19–107 years	Sodium and potassium intake; 61-item semiquantitative FFQ	Incidence of non-fatal myocardial infarction or acute ischemic stroke
Wang et al., 2022b (255)	China	Prospective cohort studies with median follow-up 6 years	China Family Panel Studies (CFPS)	20,688 (49%); 49 ± 13 years	Egg intake; FFQ	Incidence of total cardiovascular disease
Wang et al., 2022c (256)	USA	Prospective cohort studies with mean follow-up 13.6 years	Prostate, Lung, Colorectal, and Ovarian (PLCO) Cancer Screening Trial	86,633 (46%); 55-74 years	Type 2 diabetes–prevention diet; 137-item FFQ	Cardiovascular disease mortality
Wang et al., 2023a (257)	USA	Prospective cohort studies with mean follow-up 12 years	Southern Community Cohort Study (SCCS)	77,060 (40%); 52 ± 9 years	Ultra-processed food consumption; 89-item FFQ	Coronary heart disease and stroke mortality
Wang et al., 2023b (258)	USA	Prospective cohort studies with mean follow-up 4 years	Million Veteran Program (MVP)	315,919 (92%); 66 years	Plant-based diet index; 61-item semiquantitative FFQ	Cardiovascular disease mortality
Wang et al., 2023c (259)	China	Prospective cohort studies with mean follow-up 3.8 years	Chinese Longitudinal Healthy Longevity Survey (CLHLS)	9,740 (43%); 88 ± 11 years	Dietary fruits and vegetables intake; FFQ	Incidence of cardiovascular disease
Wang et al., 2023d (260)	UK	Prospective cohort studies with median follow-up 19.3 years	British Regional Heart Study	2,873 (100%); 60–79 years	Elderly Dietary Index; self-administered FFQ	Incidence of myocardial infarction and stroke
Wang et al., 2024a (261)	USA	Prospective cohort studies with mean follow-up 27.4 years	Nurses' health study (NHS), NHSII, and Health Professionals Follow-up Study (HPFS)	201,244 (not reported); 25-75 years	Phytosterol intake; FFQ	Incidence of coronary artery disease
Wang et al., 2024b (262)	USA	Prospective cohort studies with mean follow-up 3.8 years	Million Veteran Program (MVP)	148,506 (88%); 59-62 years	Red meat intake; semiquantitative FFQ	Incidence of cardiovascular disease
Ward et al., 2020 (263)	USA	Prospective cohort studies with mean follow-up 2.2 to 4.7 years	Million Veteran Program (MVP)	197,761 (92%); 66 ± 12 years	Intake of omega-3 fatty acid; FFQ	Incidence of non-fatal coronary artery disease and ischemic stroke
Weikart et al., 2022 (264)	USA	Prospective cohort studies with median follow-up 6.3 years	California Teachers Study	18,533 (0%); 56 ± 12 years	Pre-cancer diagnosis diet quality; 103-item semiquantitative FFQ	Cardiovascular disease mortality

Author, year	Country	Study design and mean/median follow-up period	Cohort used	No. of participants (% of male) and age at baseline	Dietary exposure and assessment tool	CVD outcomes
Weston et al., 2022 (265)	USA	Prospective cohort studies with median follow-up 13 to 15 years	Jackson Heart Study	3,635 (36%); 21–95 years	Plant-based diet; 158-item FFQ	Cardiovascular disease mortality
Wu et al., 2020 (266)	USA	Prospective cohort studies with median follow-up 8.1 years	Women's Health Initiative (WHI)	97,725 (0%); 50-70 years	Dietary magnesium intake; 122-item FFQ	Incidence of heart failure
Würtz et al., 2021 (267)	Denmark	Prospective cohort studies with mean follow-up 13.6 years	Danish Diet, Cancer, and Health cohort	55,171 (47%); 50-64 years	Replacement of potatoes with other vegetables; 192-item semi-quantitative FFQ	Incidence of myocardial infarction
Xia et al., 2020 (268)	China	Prospective cohort studies with mean follow-up 7.6 years	Prediction for Atherosclerotic Cardiovascular Disease Risk in China (China-PAR)	102,136 (40%); 51 years	Egg consumption; FFQ	Incidence of cardiovascular disease, coronary heart disease, stroke, ischemic stroke, and hemorrhagic stroke.
Xia et al., 2023 (269)	China	Prospective cohort studies with mean follow-up 11.7 years	China Multi-Center Collaborative Study of Cardiovascular Epidemiology (ChinaMUCA) (1998), the International Collaborative Study of Cardiovascular Disease in Asia (InterASIA), and the Community Intervention of Metabolic Syndrome in China & Chinese Family Health Study (CIMIC)	34,111 (42%); 52 ± 11 years	Egg consumption; cohort-specific FFQ	Incidence of coronary artery disease
Xu et al., 2020 (270)	USA	Prospective cohort studies with median follow-up 26 years	Atherosclerosis Risk in Communities (ARIC) study	10,808 (44%); 54 ± 6 years	The Healthy Eating Index (HEI)-2015 and the Alternative HEI (AHEI)-2010 score; 66-item FFQ	Incidence of cardiovascular disease
Xue et al., 2022 (271)	China	Prospective cohort studies with median follow-up 3.16 years	4C study	97,930 (34%); 40 years or above	Soy intake; 16-item FFQ	Incidence of CVD events (heart failure, non-fatal stroke, non-fatal myocardial infarction, and cardiovascular mortality)
Yamakawa et al., 2019 (272)	Japan	Prospective cohort studies with mean follow-up 14.1 years	Takayama Study	29,079 (46%); 35 years or above	Coffee consumption; 169-item FFQ	Cardiovascular disease mortality
Yamakawa et al., 2022 (273)	Japan	Prospective cohort studies with mean follow-up 13.7 years	Takayama Study	31,552 (42%); 35 years or above	Total nut and peanut intakes; 169-item FFQ	Cardiovascular disease mortality
Yang et al., 2020a (274)	China and USA	Prospective cohort studies with median follow-up 11.7 years	Southern Community Cohort Study (SCCS), Shanghai Men's Health Study (SMHS), and Shanghai Women's Health Study (SWHS)	207,625 (not reported); 40 - 79 years	Choline-related nutrients; FFQ	Ischemic heart disease and stroke mortality
Yang et al., 2020b (275)	Japan	Prospective cohort studies with mean follow-up 13.2 years	Japan Public Health Center–based Prospective (JPHC) Study	87,177 (46%); 57 ± 8 years	Consumption of flavonoid-rich fruits; FFQ	Incidence of coronary heart disease (CHD)
Yang et al., 2022 (276)	China	Prospective cohort studies with median follow-up 11 years	China Kadoorie Biobank study	461,047 (41%); 52 ± 11 years	Coarse grain consumption; semiquantitative FFQ	Incidence of cardiovascular disease, ischemic stroke, hemorrhagic stroke, fatal ischemic heart disease, and nonfatal myocardial infarction
Yao et al., 2021 (277)	USA	Prospective cohort studies with median follow-up 17 years	Prostate, Lung, Colorectal, and Ovarian (PLCO) Cancer Screening Trial	101,832 (49%); 62 ± 5 years	Dietary fat intake; 124-item DHQ	Cardiovascular disease mortality
Yazdanpanah et al., 2024 (278)	Iran	Prospective cohort studies with median follow-up 14.1 years	Golestan Cohort Study (GCS)	41,863 (43%); 40-75 years	Mineral intake; 116-item semiquantitative FFQ	Cardiovascular disease mortality
Yeung et al., 2021 (279)	China	Prospective cohort studies with mean follow-up 14 years	Mr. OS and Ms. OS study	3,992 (50%); 68-76 years	Fruit and vegetable variety; 280-item semi-quantitative FFQ	Cardiovascular disease mortality
Yoshizaki et al., 2020 (280)	Japan	Prospective cohort studies with mean follow-up 13.2 years	Japan Public Health Center–based Prospective (JPHC) Study	16,498 (47%); 45 - 74 years	Vegetable, fruit, and Okinawan vegetable consumption; 138-item FFQ	Incident Stroke and Coronary heart disease
Yoshizaki et al., 2022 (281)	Japan	Prospective cohort studies with mean follow-up 13.3 years	Japan Public Health Center–based Prospective (JPHC) Study	78,115 (46%); 45 - 74 years	Vegetable and fruit consumption; 138-item FFQ	Incidence of stroke and coronary heart disease
Yuan et al., 2022 (282)	Sweden	Prospective cohort studies with mean follow-up 18.1 years	Swedish Infrastructure for Medical Population based Life course Environmental Research (SIMPLER)	69,449 (54%); 45-83 years	A modified Mediterranean diet scale; 96-item semiquantitative FFQ	Incidence of peripheral artery disease (PAD)
Zhang et al., 2021 (283)	USA	Prospective cohort studies with median follow-up 16 years	National Institutes of Health-AARP (NIH-AARP) Diet and Health Study	521,120 (59%); 50 - 71 years	Cooking oil/fat consumption; 124-item FFQ (DHQ)	Cardiovascular disease mortality
Zhang et al., 2023 (284)	Sweden	Prospective cohort studies with mean follow-up 21 years	Malmö Diet and Cancer Study (MDCS)	20,499 (38%); 58 ± 8 years	Milk intake; 168-item FFQ	Cardiovascular disease mortality
Zhang et al., 2024a (285)	China	Prospective cohort studies with mean follow-up 3.9 years	Henan Rural Cohort	22,536 (39%); 55 ± 12 years	Healthy diet habits; FFQ	Incidence of non-fatal ischemic stroke
Zhang et al., 2024b (286)	USA	Prospective cohort studies with mean follow-up 32 years	Nurses' health study (NHS), NHSII, and Health Professionals Follow-up Study (HPFS)	41,714 (16%); 25-75 years	Total fruits and vegetables intake; semiquantitative FFQ	Incidence of atherosclerotic cardiovascular disease
Zhao et al., 2021 (287)	USA	Prospective cohort studies with mean follow-up 14.4 years	Coronary Artery Risk Development in Young Adults (CARDIA) Study	1,928 (46%); 46 ± 3 years	Dairy foods, fruits, nuts and legumes, processed meat, red meat, refined grains, seafood, sugar-sweetened beverages, starchy vegetables, non starchy vegetables, whole grains, dietary sodium; interviewer-administrated FFQ	Incidence of atherosclerotic cardiovascular disease
Zhao et al., 2023 (288)	USA	Prospective cohort studies with median follow-up 23.5 years	National Institutes of Health-AARP (NIH-AARP) Diet and Health Study	369,827 (56%); 61 ± 5 years	Dietary flavonoids and subclasses; 124-item FFQ	Cardiovascular disease mortality
Zhao et al., 2024 (289)	USA	Prospective cohort studies with mean follow-up 24 years	National Institutes of Health-AARP (NIH-AARP) Diet and Health Study	407,531 (57%); 61 ± 5 years	Plant and animal fat intake; 124-item diet history questionnaire	Cardiovascular disease mortality
Zheng et al., 2023 (290)	USA	Prospective cohort studies with median follow-up 13 years	Women's Health Initiative (WHI)	3,434 (0%); 65-66 years	Energy-adjusted dietary inflammatory index scores (E-DII); FFQ	Cardiovascular disease mortality
Zhong et al., 2020 (291)	USA	Prospective cohort studies with median follow-up 19 years	Atherosclerosis Risk in Communities (ARIC), Coronary Artery Risk Development in Young Adults (CARDIA), Cardiovascular Health Study (CHS), Framingham Heart Study (FHS), Framingham Offspring Study (FOS), and Multi-Ethnic Study of Atherosclerosis (MESA).	29,682 (44%); 54 ± 16 years	Processed meat, unprocessed red meat, poultry, or fish intake; FFQ	Incidence cardiovascular disease and mortality
Zhong et al., 2021a (292)	USA	Prospective cohort studies with mean follow-up 13.5 years	Prostate, Lung, Colorectal, and Ovarian (PLCO) Cancer Screening Trial	91,891 (46%); 64 - 66 years	Ultra-processed food consumption; 137-item DHQ	Cardiovascular disease mortality
Zhong et al., 2021b (293)	USA	Prospective cohort studies with mean follow-up 13.5 years	Prostate, Lung, Colorectal, and Ovarian (PLCO) Cancer Screening Trial	91,891 (46%); 65 ± 6 years	Chocolate consumption; DHQ	Cardiovascular disease mortality

Author, year	Country	Study design and mean/median follow-up period	Cohort used	No. of participants (% of male) and age at baseline	Dietary exposure and assessment tool	CVD outcomes
Zhong et al., 2021c (294)	USA	Prospective cohort studies with mean follow-up 40 years	Atherosclerosis Risk in Communities (ARIC), Coronary Artery Risk Development in Young Adults (CARDIA), Cardiovascular Health Study (CHS), Framingham Heart Study (FHS), Framingham Offspring Study (FOS), and Multi-Ethnic Study of Atherosclerosis (MESA).	29,497 (45%); 53 ± 15 years	The alternate Healthy Eating Index 2010 (aHEI-2010), alternate Mediterranean (aMED) diet score, and Dietary Approaches to Stop Hypertension (DASH) score; FFQ or diet history	Incidence of cardiovascular disease
Zhong et al., 2021d (295)	USA	Prospective cohort studies with mean follow-up 19 years	Atherosclerosis Risk in Communities (ARIC), Coronary Artery Risk Development in Young Adults (CARDIA), Cardiovascular Health Study (CHS), Framingham Heart Study (FHS), Framingham Offspring Study (FOS), and Multi-Ethnic Study of Atherosclerosis (MESA).	29,682 (44%); 54 ± 16 years	Protein foods from animal sources; diet history or FFQ	Incidence of cardiovascular disease, cardiovascular mortality
Zhuang et al., 2019 (296)	USA	Prospective cohort studies with median follow-up 16 years	National Institutes of Health-AARP (NIH-AARP) Diet and Health Study	521,120 (59%); 50 - 71 years	Dietary fat intake; 124-item FFQ	Cardiovascular disease mortality
Zhuang et al., 2021 (297)	USA	Prospective cohort studies with mean follow-up 16 years	National Institutes of Health-AARP (NIH-AARP) Diet and Health Study	521,120 (59%); 50–71 years	Egg and cholesterol consumption; 124-item FFQ	Cardiovascular disease mortality
Zhuang et al., 2023 (298)	China	Prospective cohort studies with mean follow-up 8.6 years	China Kadoorie Biobank study	487,212 (not reported); not reported	Dairy consumption; FFQ	Incidence of incident total cardiovascular disease, coronary heart disease and stroke

Supplementary Table S2. Quality assessment of included cohort studies (n=298).

Author, year	Selection cohorts	of Comparability of Cohorts	Ascertainment of Outcomes	Nutrition-Specific	Overall study rating
Abris et al., 2024 (1)	●	●	○	○	○
Akter et al., 2020 (2)	●	●	○	●	●
Al-Ramady et al., 2022 (3)	●	●	●	●	●
Al-Shaar et al., 2020 (4)	●	●	○	○	○
Amba et al., 2019 (5)	●	●	●	○	●
Arnesen et al., 2024a (6)	●	●	●	○	●
Arnesen et al., 2024b (7)	●	●	○	○	○
Asadi et al., 2019 (8)	●	●	○	○	○
Asadi et al., 2020 (9)	○	●	○	○	○
Asghari et al., 2022 (10)	●	●	○	●	●
Atefatfar et al., 2023 (11)	●	●	○	○	○
Baden et al., 2019 (12)	●	●	○	●	●
Bahadoran et al., 2022 (13)	●	●	○	●	●
Bellinge et al., 2021 (14)	●	●	○	○	○
Blekkenhorst et al., 2020 (15)	●	●	●	●	●
Bodar et al., 2020 (16)	●	●	○	○	○
Bodar et al., 2021 (17)	○	○	○	-	-
Bonaccio et al., 2023 (18)	○	●	○	○	○
Bonekamp et al., 2024 (19)	●	●	○	●	●
Bork et al., 2019 (20)	●	●	○	○	○
Bork et al., 2023 (21)	●	●	○	○	○
Budhathoki et al., 2019 (22)	●	●	○	●	●
Bui et al., 2024 (23)	●	●	○	○	○
Buziau et al., 2019 (24)	●	●	○	○	○
Carballo-Casla et al., 2024 (25)	●	●	●	●	●
Carroll et al., 2024 (26)	●	●	○	○	○
Castañeda et al., 2024 (27)	●	●	●	●	●
Chan et al., 2019 (28)	●	●	○	●	●
Chen et al., 2021 (29)	●	●	○	○	○
Chen et al., 2023a (30)	●	●	●	●	●
Chen et al., 2023b (31)	●	●	●	●	●
Chiu et al., 2020 (32)	●	●	○	○	○
Chuang et al., 2021 (33)	●	●	○	●	●
Chung et al., 2023 (34)	●	●	○	○	○
Colizzi et al., 2023 (35)	●	●	○	○	○

Author, year	Selection cohorts	of Comparability of Cohorts	Ascertainment of Outcomes	Nutrition-Specific	Overall study rating
Cordova et al., 2023 (36)	○	●	●	○	●
Critselis et al., 2021 (37)	●	●	○	●	●
Critselis et al., 2023 (38)	●	●	●	●	●
Cruijsen et al., 2021 (39)	●	●	○	●	●
Cui et al., 2022 (40)	○	○	○	-	-
Cupino et al., 2022 (41)	●	●	○	●	●
Dalgaard et al., 2019 (42)	●	●	○	○	○
Damigou et al., 2023 (43)	●	●	○	●	●
Damigou et al., 2024a (44)	●	●	○	○	○
Damigou et al., 2024b (45)	●	●	○	○	○
Das et al., 2021 (46)	●	●	○	○	○
Das et al., 2022 (47)	●	●	○	○	○
Davitte et al., 2021 (48)	○	○	●	○	○
de la O et al., 2022 (49)	●	●	○	○	○
de Souza et al., 2020 (50)	●	●	○	○	○
Dehghan et al., 2020 (51)	●	●	●	●	●
Dehghan et al., 2023 (52)	●	●	●	●	●
Dennis et al., 2023 (53)	●	●	●	●	●
Devaraj et al., 2021 (54)	●	●	○	○	●
Dibaba et al., 2019 (55)	●	●	○	○	●
Djoussé et al., 2020 (56)	●	●	●	●	●
Djoussé et al., 2021 (57)	○	○	○	-	○
Donat-Vargas et al., 2020 (58)	●	●	●	●	●
Du et al., 2021 (59)	●	●	●	●	●
Dupuy et al., 2024 (60)	●	●	○	○	○
Ergas et al., 2024 (61)	○	○	○	-	-
Evers et al., 2022 (62)	●	●	○	●	●
Fang et al., 2024 (63)	●	●	○	○	○
Farhadnejad et al., 2020 (64)	●	●	○	●	●
Frederiksen et al., 2021 (65)	●	●	○	○	○
Fruh et al., 2024 (66)	●	●	○	○	○
Fu et al., 2024 (67)	●	●	○	○	○
Gaeini et al., 2019 (68)	●	●	●	●	●
Gaeini et al., 2021 (69)	●	●	●	●	●
Gamba et al., 2023 (70)	●	●	○	○	○
Ganbat et al., 2024 (71)	●	●	○	○	○

Author, year	Selection cohorts	of Comparability of Cohorts	Ascertainment of Outcomes	Nutrition-Specific	Overall study rating
Gao et al., 2021a (72)	●	●	○	●	●
Gao et al., 2021b (73)	●	●	○	○	○
Ge et al., 2023 (74)	●	●	●	●	●
Georgoulis et al., 2024 (75)	●	●	○	○	○
Glenn et al., 2021 (76)	●	●	○	○	○
Glenn et al., 2023 (77)	●	●	○	○	○
Golzarand et al., 2022 (78)	●	●	○	○	○
Gómez-Donoso et al., 2021 (79)	●	●	○	○	○
Grau et al., 2022 (80)	●	●	○	○	○
Gu et al., 2022 (81)	●	●	○	○	○
Guasch-Ferré et al., 2019 (82)	●	●	●	●	●
Guasch-Ferre et al., 2020 (83)	●	●	○	●	●
Guasch-Ferré et al., 2022 (84)	●	●	○	●	●
Hansen et al., 2021 (85)	●	●	●	●	●
Harbers et al., 2020 (86)	●	●	○	●	●
Hashemian et al., 2019a (87)	●	●	○	●	●
Hashemian et al., 2019b (88)	●	●	○	●	●
Haugsgjerd et al., 2020 (89)	●	●	●	●	●
Haugsgjerd et al., 2022 (90)	●	●	●	●	●
Hejazi et al., 2020 (91)	●	●	○	●	●
Hirahatake et al., 2019 (92)	●	●	●	●	●
Hjelmgaard et al., 2023 (93)	●	●	○	○	○
Horikawa et al., 2019 (94)	●	●	●	●	●
Horikawa et al., 2021 (95)	●	●	○	○	○
Hosseini-Esfahani et al., 2020 (96)	●	●	○	●	●
Hu et al., 2019 (97)	●	●	●	●	●
Hu et al., 2020 (98)	●	●	○	○	○
Hu et al., 2021 (99)	●	●	○	○	○
Hu et al., 2022 (100)	●	●	●	●	●
Huang et al., 2020 (101)	●	●	●	●	●
Huang et al., 2021a (102)	●	●	●	●	●
Huang et al., 2021b (103)	●	●	○	○	○
Ibsen et al., 2022 (104)	●	●	●	●	●
Ikehara et al., 2021 (105)	●	●	○	○	○

Author, year	Selection of cohorts	Comparability of Cohorts	Ascertainment of Outcomes	Nutrition-Specific	Overall study rating
Im et al., 2021 (106)	●	●	○	○	○
Imran et al., 2021 (107)	●	●	○	○	○
Ivey et al., 2021 (108)	●	●	○	○	○
Jackson et al., 2019a (109)	●	●	●	●	●
Jackson et al., 2019b (110)	●	●	●	●	●
Jackson et al., 2020 (111)	●	●	●	●	●
Jalali et al., 2024 (112)	●	●	○	●	●
Jeon & Park, 2019 (113)	●	●	○	○	○
Jeong et al., 2023 (114)	●	●	●	●	●
Jo & Park, 2023 (115)	●	●	○	○	○
Johansson et al., 2019 (116)	●	●	○	●	●
Johansson et al., 2020 (117)	○	○	○	-	-
Jung et al., 2023 (118)	●	●	○	○	○
Juul et al., 2021 (119)	●	●	○	○	○
Kang et al., 2020 (120)	●	●	○	●	●
Kashino et al., 2019 (121)	●	●	○	●	●
Katagiri et al., 2020a (122)	●	●	●	●	●
Katagiri et al., 2020b (123)	●	●	●	●	●
Kazemi et al., 2022 (124)	●	●	○	●	●
Keller et al., 2020 (125)	●	●	●	●	●
Kermani-Alghoraishi et al., 2024 (126)	●	●	●	●	●
Khan et al., 2020 (127)	●	●	○	○	○
Kim et al., 2021 (128)	●	●	○	○	○
Kityo & Lee, 2023 (129)	●	●	○	○	○
Kjeldsen et al., 2022 (130)	○	○	●	○	○
Kouvari et al., 2020a (131)	●	●	●	●	●
Kouvari et al., 2020b (132)	●	●	●	●	●
Kouvari et al., 2022 (133)	●	●	○	○	○
Kvist et al., 2020 (134)	●	●	○	●	●
Kwon et al., 2022 (135)	○	○	○	-	-
Kwon et al., 2023 (136)	●	●	○	-	-
Langsetmo et al., 2020 (137)	○	○	○	-	-
Lara et al., 2019 (138)	●	●	○	○	○
Laursen et al., 2019 (139)	●	●	○	○	○
Lee et al., 2019 (140)	●	●	●	●	●

Author, year	Selection cohorts	of Comparability of Cohorts	Ascertainment of Outcomes	Nutrition-Specific	Overall study rating
Li et al., 2020a (141)	●	●	●	●	●
Li et al., 2020b (142)	●	●	○	○	○
Li et al., 2021 (143)	●	●	○	○	○
Li et al., 2023a (144)	●	●	○	○	○
Li et al., 2023b (145)	○	○	○	-	-
Liang et al., 2022 (146)	●	●	○	○	○
Lilja et al., 2019 (147)	○	○	○	-	-
Lim et al., 2022 (148)	●	●	○	●	●
Liu et al., 2019a (149)	●	●	●	●	●
Liu et al., 2019b (150)	●	●	●	●	●
Liu et al., 2020 (151)	●	●	●	●	●
Liu et al., 2021 (152)	●	●	●	●	●
Liu et al., 2023 (153)	●	●	●	●	●
Liu et al., 2024a (154)	○	○	○	-	-
Liu et al., 2024b (155)	●	●	●	●	●
Livingstone et al., 2022 (156)	●	●	○	○	○
Lo et al., 2021 (157)	●	●	○	●	●
Lukic et al., 2020 (158)	●	●	●	●	●
Luong et al., 2023 (159)	●	●	●	●	●
Luong et al., 2024 (160)	●	●	○	○	○
Ma et al., 2020 (161)	●	●	●	●	●
Ma et al., 2023 (162)	●	●	●	●	●
Malik et al., 2019 (163)	●	●	●	●	●
Mao et al., 2022 (164)	●	●	○	○	○
Matre et al., 2021 (165)	○	○	○	-	-
Matsuyama et al., 2021 (166)	●	●	●	●	●
Mendonça et al., 2019 (167)	●	●	●	●	●
Meng et al., 2023a (168)	●	●	○	○	○
Meng et al., 2023b (169)	●	●	○	○	○
Michaëlsson et al., 2020 (170)	●	●	○	○	○
Mirmiran et al., 2020 (171)	●	●	○	○	○
Mirmiran et al., 2023 (172)	●	●	○	○	○
Mohammadifard et al., 2021 (173)	●	●	○	●	●
Mohammadifard et al., 2022 (174)	●	●	●	●	●

Author, year	Selection cohorts	of Comparability of Cohorts	Ascertainment of Outcomes	Nutrition-Specific	Overall study rating
Mohan et al., 2021 (175)	●	●	●	●	●
Mohseni et al., 2023 (176)	●	●	○	○	○
Mori et al., 2019 (177)	●	●	○	○	○
Mosallanezhad et al., 2023 (178)	●	●	○	●	●
Murai et al., 2019 (179)	●	●	●	●	●
Musicus et al., 2022 (180)	●	●	○	●	●
Nanri et al., 2023 (181)	●	●	●	●	●
Norouzzadeh et al., 2024 (182)	●	●	○	●	●
Nozue et al., 2021 (183)	●	●	○	●	●
Oh et al., 2022 (184)	●	●	○	●	●
Okada et al., 2019 (185)	●	●	○	○	○
Pacheco et al., 2020 (186)	●	●	●	●	●
Pacheco et al., 2022a (187)	●	●	●	●	●
Pacheco et al., 2022b (188)	●	●	●	●	●
Palmer et al., 2021 (189)	●	●	○	○	○
Pan et al., 2021 (190)	●	●	○	○	○
Panagiotakos et al., 2019 (191)	●	●	●	●	●
Papandreou et al., 2019 (192)	●	●	○	○	○
Parmenter et al., 2021 (193)	○	○	○	-	-
Parmenter et al., 2023a (194)	●	●	○	○	○
Parmenter et al., 2023b (195)	●	●	○	○	○
Patel et al., 2021 (196)	●	●	●	●	●
Pertiwi et al., 2021 (197)	●	●	●	●	●
Praagman et al., 2019 (198)	●	●	○	●	●
Ruggiero et al., 2021a (199)	●	●	●	●	●
Ruggiero et al., 2021b (200)	●	●	●	●	●
Ruggiero et al., 2024 (201)	●	●	●	●	●
Sadeghi et al., 2021 (202)	●	●	○	●	●
Saglimbene et al., 2019a (203)	●	●	●	●	●
Saglimbene et al., 2019b (204)	●	●	○	●	●
Saglimbene et al., 2020 (205)	●	●	○	●	●
Sahashi et al., 2022 (206)	●	●	●	●	●
Saito et al., 2020 (207)	●	●	●	●	●
Sakamaki et al., 2021 (208)	●	●	●	●	●
Sawicki et al., 2024 (209)	●	●	○	○	○

Author, year	Selection cohorts	of Comparability of Cohorts	Ascertainment of Outcomes	Nutrition-Specific	Overall study rating
Scheffers et al., 2019 (210)	●	●	●	●	●
Schmidt et al., 2020 (211)	●	●	●	●	●
Shams-White et al., 2022 (212)	○	○	○	-	-
Shan et al., 2020 (213)	●	●	●	●	●
Shao et al., 2022 (214)	●	●	●	●	●
Sheng et al., 2022 (215)	●	●	○	○	○
Shikany et al., 2021 (216)	●	●	○	○	○
Shin et al., 2022 (217)	●	●	○	○	○
Silva et al., 2022 (218)	●	●	○	○	○
Sjöblom et al., 2024 (219)	●	●	○	○	○
Son et al., 2019 (221)	●	●	●	●	●
Son et al., 2024 (220)	●	●	●	●	●
Stefler et al., 2021 (222)	●	●	●	●	●
Strengers et al., 2021 (223)	●	●	○	○	○
Su et al., 2023 (224)	●	●	○	○	○
Sun et al., 2019 (225)	●	●	●	●	●
Sun et al., 2023 (226)	●	●	●	●	●
Sun et al., 2024 (227)	●	●	●	●	●
Swaminathan et al., 2021 (228)	●	●	○	○	○
Taguchi et al., 2020 (229)	●	●	○	○	○
Talaei et al., 2019a (230)	●	●	○	○	○
Talaei et al., 2019b (231)	●	●	●	●	●
Tamura et al., 2023 (232)	●	●	●	●	●
Tang et al., 2023 (233)	●	●	●	●	●
Tanno et al., 2021 (234)	●	●	○	●	●
Tong et al., 2024 (235)	●	●	●	○	○
Torres-collado et al., 2021 (236)	●	●	●	●	●
Torres-Collado et al., 2022 (237)	●	●	●	●	●
Troeschel et al., 2021 (238)	●	●	●	●	●
Troeschel et al., 2023 (239)	●	●	●	●	●
Um et al., 2019 (240)	●	●	○	○	○
Ushula et al., 2023 (241)	●	●	○	○	○
Van Parys et al., 2020 (242)	●	●	○	●	●
Vanegas et al., 2022 (243)	●	●	●	●	●

Author, year	Selection cohorts	of Comparability of Cohorts	Ascertainment of Outcomes	Nutrition-Specific	Overall study rating
Vázquez-Ruiz et al., 2022 (244)	●	●	●	●	●
Venø et al., 2019 (245)	●	●	○	●	●
Veronese et al., 2020 (246)	●	●	○	○	○
Vissers et al., 2019 (247)	●	●	●	●	●
Vogtschmidt et al., 2024 (248)	●	●	○	●	●
Voortman et al., 2021 (249)	●	●	○	●	●
Wada et al., 2022 (250)	●	●	○	○	○
Wan et al., 2022 (251)	●	●	●	●	●
Wang et al., 2021a (252)	●	●	○	○	○
Wang et al., 2021b (253)	●	●	●	●	●
Wang et al., 2022a (254)	●	●	●	●	●
Wang et al., 2022b (255)	○	○	○	-	-
Wang et al., 2022c (256)	●	●	●	●	●
Wang et al., 2023a (257)	●	●	●	○	○
Wang et al., 2023b (258)	●	●	○	○	○
Wang et al., 2023c (259)	●	●	●	●	●
Wang et al., 2023d (260)	●	●	○	○	○
Wang et al., 2024a (261)	●	●	●	●	●
Wang et al., 2024b (262)	●	●	●	●	●
Ward et al., 2020 (263)	○	○	○	-	-
Weikart et al., 2022 (264)	●	●	○	○	○
Weston et al., 2022 (265)	●	●	●	●	●
Wu et al., 2020 (266)	●	●	○	○	○
Würtz et al., 2021 (267)	●	●	○	○	○
Xia et al., 2020 (268)	○	○	●	○	○
Xia et al., 2023 (269)	○	○	●	○	○
Xu et al., 2020 (270)	●	●	●	●	●
Xue et al., 2022 (271)	○	○	○	-	-
Yamakawa et al., 2019 (272)	●	●	○	○	○
Yamakawa et al., 2022 (273)	●	●	○	○	○
Yang et al., 2020a (274)	●	●	●	●	●
Yang et al., 2020b (275)	●	●	○	●	●
Yang et al., 2022 (276)	●	●	●	●	●
Yao et al., 2021 (277)	●	●	●	●	●
Yazdanpanah et al., 2024 (278)	●	●	●	●	●

Author, year	Selection cohorts	of Comparability of Cohorts	Ascertainment of Outcomes	Nutrition-Specific	Overall study rating
Yeung et al., 2021 (279)	●	●	●	●	●
Yoshizaki et al., 2020 (280)	●	●	●	●	●
Yoshizaki et al., 2022 (281)	●	●	●	●	●
Yuan et al., 2022 (282)	●	●	○	●	●
Zhang et al., 2021 (283)	●	●	○	●	●
Zhang et al., 2023 (284)	●	●	○	○	○
Zhang et al., 2024a (285)	○	○	●	○	○
Zhang et al., 2024b (286)	●	●	○	○	○
Zhao et al., 2021 (287)	●	●	●	●	●
Zhao et al., 2023 (288)	●	●	○	○	○
Zhao et al., 2024 (289)	●	●	○	●	●
Zheng et al., 2023 (290)	○	○	○	-	-
Zhong et al., 2020 (291)	●	●	●	●	●
Zhong et al., 2021a (292)	●	●	○	○	○
Zhong et al., 2021b (293)	●	●	●	●	●
Zhong et al., 2021c (294)	●	●	○	○	○
Zhong et al., 2021d (295)	●	●	○	○	○
Zhuang et al., 2019 (296)	●	●	○	●	●
Zhuang et al., 2021 (297)	●	●	●	●	●
Zhuang et al., 2023 (298)	●	●	●	●	●

● **(Solid circle)**: *Good (+) indicates Almost all criteria met. Little or no concern over study design. Low RoB.*

○ **(Hollow circle)**: *Neutral (0)* suggests *most criteria met. Some flaws in the study with an associated concern over study design. Moderate RoB.*

– **(Dash)**: *Poor (-)* reflects *most or all criteria not met, or significant flaws related to key aspects of the study design. High RoB.*

References

1. Abris GP, Shavlik DJ, Mathew RO, Butler FM, Oh J, Sirirat R, et al. Cause-specific and all-cause mortalities in vegetarian compared with those in nonvegetarian participants from the Adventist Health Study-2 cohort. Am J Clin Nutr. 2024;120(4):907-17.

2. Akter S, Mizoue T, Nanri A, Goto A, Noda M, Sawada N, et al. Low carbohydrate diet and all cause and cause-specific mortality. Clin Nutr. 2021;40(4):2016-24.

3. Al-Ramady O, Latifi AN, Treu T, Ho YL, Seshadri S, Aparicio HJ, et al. Egg consumption and risk of acute stroke in the Million Veteran Program. Clin Nutr ESPEN. 2022;50:178-82.

4. Al-Shaar L, Satija A, Wang DD, Rimm EB, Smith-Warner SA, Stampfer MJ, et al. Red meat intake and risk of coronary heart disease among US men: prospective cohort study. Bmj. 2020;371:m4141.
5. Amba V, Murphy G, Etemadi A, Wang S, Abnet CC, Hashemian M. Nut and Peanut Butter Consumption and Mortality in the National Institutes of Health-AARP Diet and Health Study. Nutrients. 2019;11(7).
6. Arnesen EK, Laake I, Carlsen MH, Veierød MB, Retterstøl K. Potato Consumption and All-Cause and Cardiovascular Disease Mortality - A Long-Term Follow-Up of a Norwegian Cohort. J Nutr. 2024;154(7):2226-35.
7. Arnesen EK, Laake I, Veierød MB, Retterstøl K. Saturated fatty acids and total and CVD mortality in Norway: a prospective cohort study with up to 45 years of follow-up. Br J Nutr. 2024;132(4):1-13.
8. Asadi Z, Shafiee M, Sadabadi F, Heidari-Bakavoli A, Moohebati M, Khorrami MS, et al. Association of dietary patterns and risk of cardiovascular disease events in the MASHAD cohort study. J Hum Nutr Diet. 2019;32(6):789-801.
9. Asadi Z, Yaghooti-Khorasani M, Ghazizadeh H, Sadabadi F, Mosa-Farkhany E, Darroudi S, et al. Association between dietary inflammatory index and risk of cardiovascular disease in the Mashhad stroke and heart atherosclerotic disorder study population. IUBMB Life. 2020;72(4):706-15.
10. Asghari G, Farhadnejad H, Teymoori F, Emamat H, Shahrzad MK, Habibi-Moeini AS, et al. Association of Dietary Diabetes Risk Reduction Score With Risk of Cardiovascular Diseases in the Iranian Population: Tehran Lipid and Glucose Study. Heart Lung Circ. 2022;31(1):101-9.
11. Atefatfar A, Babajafari S, Mohammadifard N, Nouri F, Boshtam M, Sadeghi M, et al. A healthy diet, physical activity, or either in relation to cardiovascular and all-cause mortality: A prospective cohort study. Nutrition. 2023;116:112186.
12. Baden MY, Liu G, Satija A, Li Y, Sun Q, Fung TT, et al. Changes in Plant-Based Diet Quality and Total and Cause-Specific Mortality. Circulation. 2019;140(12):979-91.
13. Bahadoran Z, Mirmiran P, Azizi F. Dietary oxalate to calcium ratio and incident cardiovascular events: a 10-year follow-up among an Asian population. Nutr J. 2022;21(1):21.
14. Bellinge JW, Dalgaard F, Murray K, Connolly E, Blekkenhorst LC, Bondonno CP, et al. Vitamin K Intake and Atherosclerotic Cardiovascular Disease in the Danish Diet Cancer and Health Study. J Am Heart Assoc. 2021;10(16):e020551.
15. Blekkenhorst LC, Lewis JR, Bondonno CP, Sim M, Devine A, Zhu K, et al. Vegetable diversity in relation with subclinical atherosclerosis and 15-year atherosclerotic vascular disease deaths in older adult women. Eur J Nutr. 2020;59(1):217-30.
16. Bodar V, Chen J, Sesso HD, Gaziano JM, Djoussé L. Coffee consumption and risk of heart failure in the Physicians' Health Study. Clin Nutr ESPEN. 2020;40:133-7.

17. Bodar V, Ho YL, Cho K, Gagnon D, Gaziano JM, Djoussé L. Consumption of potatoes and incidence rate of coronary artery disease: The Million Veteran Program. Clin Nutr ESPEN. 2021;42:201-5.
18. Bonaccio M, Di Castelnuovo A, Costanzo S, Ruggiero E, Esposito S, Persichillo M, et al. Ultraprocessed food consumption is associated with all-cause and cardiovascular mortality in participants with type 2 diabetes independent of diet quality: a prospective observational cohort study. Am J Clin Nutr. 2023;118(3):627-36.
19. Bonekamp NE, Geleijnse JM, van der Schouw YT, Dorresteijn JAN, van der Meer MG, Ruigrok YM, et al. Dietary habits and compliance with dietary guidelines in patients with established cardiovascular disease. Eur J Clin Nutr. 2024;78(8):709-17.
20. Bork CS, Lasota AN, Lundbye-Christensen S, Jakobsen MU, Tjønneland A, Calder PC, et al. Intake of α-linolenic acid is not consistently associated with a lower risk of peripheral artery disease: results from a Danish cohort study. Br J Nutr. 2019;122(1):86-92.
21. Bork CS, Lundbye-Christensen S, Venø SK, Lasota AN, Tjønneland A, Schmidt EB, et al. Intake of marine and plant-derived n-3 fatty acids and development of atherosclerotic cardiovascular disease in the Danish Diet, Cancer and Health cohort. Eur J Nutr. 2023;62(3):1389-401.
22. Budhathoki S, Sawada N, Iwasaki M, Yamaji T, Goto A, Kotemori A, et al. Association of Animal and Plant Protein Intake With All-Cause and Cause-Specific Mortality in a Japanese Cohort. JAMA Intern Med. 2019;179(11):1509-18.
23. Bui LP, Pham TT, Wang F, Chai B, Sun Q, Hu FB, et al. Planetary Health Diet Index and risk of total and cause-specific mortality in three prospective cohorts. Am J Clin Nutr. 2024;120(1):80-91.
24. Buziau AM, Soedamah-Muthu SS, Geleijnse JM, Mishra GD. Total Fermented Dairy Food Intake Is Inversely Associated with Cardiovascular Disease Risk in Women. J Nutr. 2019;149(10):1797-804.
25. Carballo-Casla A, Stefler D, Ortolá R, Chen Y, Knuppel A, Kubinova R, et al. The Southern European Atlantic diet and all-cause and cause-specific mortality: a European multicohort study. Eur J Prev Cardiol. 2024;31(3):358-67.
26. Carroll HA, Ericson U, Ottosson F, Enhörning S, Melander O. The association between water intake and future cardiometabolic disease outcomes in the Malmö Diet and Cancer cardiovascular cohort. PLoS One. 2024;19(1):e0296778.
27. Castañeda J, Almanza-Aguilera E, Monge A, Lozano-Esparza S, Hernández-Ávila JE, Lajous M, et al. Dietary Intake of (Poly)phenols and Risk of All-Cause and Cause-Specific Mortality in the Mexican Teachers' Cohort Study. J Nutr. 2024;154(8):2459-69.
28. Chan R, Leung J, Woo J. High Protein Intake Is Associated with Lower Risk of All-Cause Mortality in Community-Dwelling Chinese Older Men and Women. J Nutr Health Aging. 2019;23(10):987-96.

29. Chen GC, Chen LH, Mossavar-Rahmani Y, Kamensky V, Shadyab AH, Haring B, et al. Dietary cholesterol and egg intake in relation to incident cardiovascular disease and all-cause and cause-specific mortality in postmenopausal women. Am J Clin Nutr. 2021;113(4):948-59.
30. Chen Z, Hu Y, Hu FB, Manson JE, Rimm EB, Doria A, et al. Dietary Glutamine and Glutamate in Relation to Cardiovascular Disease Incidence and Mortality in the United States Men and Women with Diabetes Mellitus. J Nutr. 2023;153(11):3247-58.
31. Chen Z, Qian F, Hu Y, Voortman T, Li Y, Rimm EB, et al. Dietary phytoestrogens and total and cause-specific mortality: results from 2 prospective cohort studies. Am J Clin Nutr. 2023;117(1):130-40.
32. Chiu THT, Chang HR, Wang LY, Chang CC, Lin MN, Lin CL. Vegetarian diet and incidence of total, ischemic, and hemorrhagic stroke in 2 cohorts in Taiwan. Neurology. 2020;94(11):e1112-e21.
33. Chuang SY, Chang HY, Fang HL, Lee SC, Hsu YY, Yeh WT, et al. The Healthy Taiwanese Eating Approach is inversely associated with all-cause and cause-specific mortality: A prospective study on the Nutrition and Health Survey in Taiwan, 1993-1996. PLoS One. 2021;16(5):e0251189.
34. Chung S, Hwang JT, Joung H, Shin S. Associations of Meat and Fish/Seafood Intake with All-Cause and Cause-Specific Mortality from Three Prospective Cohort Studies in Korea. Mol Nutr Food Res. 2023;67(16):e2200900.
35. Colizzi C, Harbers MC, Vellinga RE, Verschuren WMM, Boer JMA, Biesbroek S, et al. Adherence to the EAT-Lancet Healthy Reference Diet in Relation to Risk of Cardiovascular Events and Environmental Impact: Results From the EPIC-NL Cohort. J Am Heart Assoc. 2023;12(8):e026318.
36. Cordova R, Viallon V, Fontvieille E, Peruchet-Noray L, Jansana A, Wagner KH, et al. Consumption of ultra-processed foods and risk of multimorbidity of cancer and cardiometabolic diseases: a multinational cohort study. Lancet Reg Health Eur. 2023;35:100771.
37. Critselis E, Kontogianni MD, Georgousopoulou E, Chrysohoou C, Tousoulis D, Pitsavos C, et al. Comparison of the Mediterranean diet and the Dietary Approach Stop Hypertension in reducing the risk of 10-year fatal and non-fatal CVD events in healthy adults: the ATTICA Study (2002-2012). Public Health Nutr. 2021;24(9):2746-57.
38. Critselis E, Tsiampalis T, Damigou E, Georgousopoulou E, Barkas F, Chrysohoou C, et al. High fish intake rich in n-3 polyunsaturated fatty acids reduces cardiovascular disease incidence in healthy adults: The ATTICA cohort study (2002-2022). Front Physiol. 2023;14:1158140.
39. Cruijsen E, Jacobo Cejudo MG, Küpers LK, Busstra MC, Geleijnse JM. Dairy consumption and mortality after myocardial infarction: a prospective analysis in the Alpha Omega Cohort. Am J Clin Nutr. 2021;114(1):59-69.

40. Cui S, Yi K, Wu Y, Su X, Xiang Y, Yu Y, et al. Fish Consumption and Risk of Stroke in Chinese Adults: A Prospective Cohort Study in Shanghai, China. Nutrients. 2022;14(20).
41. Cupino A, Fraser G, Knutsen S, Knutsen R, Heskey C, Sabaté J, et al. Are total omega-3 and omega-6 polyunsaturated fatty acids predictors of fatal stroke in the Adventist Health Study 2 prospective cohort? PLoS One. 2022;17(9):e0274109.
42. Dalgaard F, Bondonno NP, Murray K, Bondonno CP, Lewis JR, Croft KD, et al. Associations between habitual flavonoid intake and hospital admissions for atherosclerotic cardiovascular disease: a prospective cohort study. Lancet Planet Health. 2019;3(11):e450-e9.
43. Damigou E, Kouvari M, Chrysohoou C, Barkas F, Kravvariti E, Dalmyras D, et al. Diet Quality and Consumption of Healthy and Unhealthy Foods Measured via the Global Diet Quality Score in Relation to Cardiometabolic Outcomes in Apparently Healthy Adults from the Mediterranean Region: The ATTICA Epidemiological Cohort Study (2002-2022). Nutrients. 2023;15(20).
44. Damigou E, Detopoulou P, Antonopoulou S, Chrysohoou C, Barkas F, Vlachopoulou E, et al. Food Compass Score predicts incident cardiovascular disease: The ATTICA cohort study (2002-2022). J Hum Nutr Diet. 2024;37(1):203-16.
45. Damigou E, Chrysohoou C, Vafia C, Barkas F, Kravvariti E, Vlachopoulou E, et al. Mediterranean Diet and Cardiovascular Disease: The Moderating Role of Adequate Sleep-Results from the ATTICA Cohort Study (2002-2022). Nutrients. 2023;16(1).
46. Das A, Cumming RG, Naganathan V, Blyth F, Le Couteur DG, Handelsman DJ, et al. Dietary and supplemental antioxidant intake and risk of major adverse cardiovascular events in older men: The concord health and ageing in men project. Nutr Metab Cardiovasc Dis. 2021;31(4):1102-12.
47. Das A, Cumming R, Naganathan V, Blyth F, Couteur DGL, Handelsman DJ, et al. Associations between dietary intake of total protein and sources of protein (plant vs. animal) and risk of all-cause and cause-specific mortality in older Australian men: The Concord Health and Ageing in Men Project. J Hum Nutr Diet. 2022;35(5):845-60.
48. Davitte J, Laughlin GA, Kritz-Silverstein D, McEvoy LK. Dietary Potassium Intake and 20-Year All-Cause Mortality in Older Adults: The Rancho Bernardo Study. J Nutr Gerontol Geriatr. 2021;40(1):46-57.
49. de la OV, Zazpe I, Goni L, Santiago S, Martín-Calvo N, Bes-Rastrollo M, et al. A score appraising Paleolithic diet and the risk of cardiovascular disease in a Mediterranean prospective cohort. Eur J Nutr. 2022;61(2):957-71.
50. de Souza RJ, Dehghan M, Mente A, Bangdiwala SI, Ahmed SH, Alhabib KF, et al. Association of nut intake with risk factors, cardiovascular disease, and mortality in 16 countries from 5 continents: analysis from the Prospective Urban and Rural Epidemiology (PURE) study. Am J Clin Nutr. 2020;112(1):208-19.

51. Dehghan M, Mente A, Rangarajan S, Mohan V, Lear S, Swaminathan S, et al. Association of egg intake with blood lipids, cardiovascular disease, and mortality in 177,000 people in 50 countries. Am J Clin Nutr. 2020;111(4):795-803.
52. Dehghan M, Mente A, Rangarajan S, Mohan V, Swaminathan S, Avezum A, et al. Ultra-processed foods and mortality: analysis from the Prospective Urban and Rural Epidemiology study. Am J Clin Nutr. 2023;117(1):55-63.
53. Dennis KK, Wang F, Li Y, Manson JE, Rimm EB, Hu FB, et al. Associations of dietary sugar types with coronary heart disease risk: a prospective cohort study. Am J Clin Nutr. 2023;118(5):1000-9.
54. Devaraj SM, Miller RG, Orchard TJ, Kriska AM, Gary-Webb T, Costacou T. Data driven patterns of nutrient intake and coronary artery disease risk in adults with type 1 diabetes. J Diabetes Complications. 2021;35(10):108016.
55. Dibaba DT, Xun P, Fly AD, Bidulescu A, Tsinovoi CL, Judd SE, et al. Calcium Intake and Serum Calcium Level in Relation to the Risk of Ischemic Stroke: Findings from the REGARDS Study. J Stroke. 2019;21(3):312-23.
56. Djoussé L, Ho YL, Nguyen XT, Quaden RM, Gagnon DR, Gaziano JM, et al. Egg consumption and risk of coronary artery disease in the Million Veteran Program. Clin Nutr. 2020;39(9):2842-7.
57. Djoussé L, Zhou G, McClelland RL, Ma N, Zhou X, Kabagambe EK, et al. Egg consumption, overall diet quality, and risk of type 2 diabetes and coronary heart disease: A pooling project of US prospective cohorts. Clin Nutr. 2021;40(5):2475-82.
58. Donat-Vargas C, Bellavia A, Berglund M, Glynn A, Wolk A, Åkesson A. Cardiovascular and cancer mortality in relation to dietary polychlorinated biphenyls and marine polyunsaturated fatty acids: a nutritional-toxicological aspect of fish consumption. J Intern Med. 2020;287(2):197-209.
59. Du S, Kim H, Rebholz CM. Higher Ultra-Processed Food Consumption Is Associated with Increased Risk of Incident Coronary Artery Disease in the Atherosclerosis Risk in Communities Study. J Nutr. 2021;151(12):3746-54.
60. Dupuy M, Radavelli-Bagatini S, Zhong L, Dalla Via J, Zhu K, Blekkenhorst LC, et al. Vitamin K1 intake is associated with lower risk for all-cause and cardiovascular disease mortality in community-dwelling older Australian women. Nutr Metab Cardiovasc Dis. 2024;34(5):1189-97.
61. Ergas IJ, Cheng RK, Roh JM, Kushi LH, Kresovich JK, Iribarren C, et al. Diet quality and cardiovascular disease risk among breast cancer survivors in the Pathways Study. JNCI Cancer Spectr. 2024;8(2).
62. Evers I, Cruijsen E, Kornaat I, Winkels RM, Busstra MC, Geleijnse JM. Dietary magnesium and risk of cardiovascular and all-cause mortality after myocardial infarction: A prospective analysis in the Alpha Omega Cohort. Front Cardiovasc Med. 2022;9:936772.

63. Fang Z, Rossato SL, Hang D, Khandpur N, Wang K, Lo CH, et al. Association of ultra-processed food consumption with all cause and cause specific mortality: population based cohort study. Bmj. 2024;385:e078476.
64. Farhadnejad H, Asghari G, Teymoori F, Tahmasebinejad Z, Mirmiran P, Azizi F. Low-carbohydrate diet and cardiovascular diseases in Iranian population: Tehran Lipid and Glucose Study. Nutr Metab Cardiovasc Dis. 2020;30(4):581-8.
65. Frederiksen SB, Themsen HH, Overvad K, Dahm CC. Dietary patterns generated by the Treelet Transform and risk of stroke: a Danish cohort study. Public Health Nutr. 2021;24(1):84-94.
66. Fruh V, Babalola T, Sears C, Wellenius GA, Webster TF, Mann KK, et al. Dietary Minerals and Incident Cardiovascular Outcomes among Never-Smokers in a Danish Case-Cohort Study. Int J Environ Res Public Health. 2024;21(7).
67. Fu J, Deng Y, Ma Y, Man S, Yang X, Yu C, et al. Adherence to a Healthy Diet and Risk of Multiple Carotid Atherosclerosis Subtypes: Insights from the China MJ Health Check-Up Cohort. Nutrients. 2024;16(14).
68. Gaeini Z, Bahadoran Z, Mirmiran P, Azizi F. Tea, coffee, caffeine intake and the risk of cardio-metabolic outcomes: findings from a population with low coffee and high tea consumption. Nutr Metab (Lond). 2019;16:28.
69. Gaeini Z, Mirmiran P, Bahadoran Z, Aghayan M, Azizi F. The association between dietary fats and the incidence risk of cardiovascular outcomes: Tehran Lipid and Glucose Study. Nutr Metab (Lond). 2021;18(1):96.
70. Gamba M, Pano O, Raguindin PF, Roa-Diaz ZM, Muka T, Glisic M, et al. Association between Total Dietary Phytochemical Intake and Cardiometabolic Health Outcomes-Results from a 10-Year Follow-Up on a Middle-Aged Cohort Population. Nutrients. 2023;15(22).
71. Ganbat K, Nasan Ulzii B, Shin S. Association between plant-based diets and the risk of coronary heart disease predicted using the Framingham Risk Score in Korean men: data from the HEXA cohort study. Epidemiol Health. 2024;46:e2024035.
72. Gao Q, Dong JY, Cui R, Muraki I, Yamagishi K, Sawada N, et al. Consumption of flavonoid-rich fruits, flavonoids from fruits and stroke risk: a prospective cohort study. Br J Nutr. 2021;126(11):1717-24.
73. Gao Q, Eshak ES, Muraki I, Shirai K, Yamagishi K, Tamakoshi A, et al. The apparent inverse association between dietary carotene intake and risk of cardiovascular mortality disappeared after adjustment for other cardioprotective dietary intakes: The Japan collaborative cohort study. Nutr Metab Cardiovasc Dis. 2021;31(11):3064-75.
74. Ge S, Zha L, Sobue T, Kitamura T, Iso H, Ishihara J, et al. Associations between dairy intake and mortality due to all-cause and cardiovascular disease: the Japan Public Health Center-based prospective study. Eur J Nutr. 2023;62(5):2087-104.

75. Georgoulis M, Damigou E, Chrysohoou C, Barkas F, Anastasiou G, Kravvariti E, et al. Mediterranean diet trajectories and 20-year incidence of cardiovascular disease: The ATTICA cohort study (2002-2022). Nutr Metab Cardiovasc Dis. 2024;34(1):153-66.
76. Glenn AJ, Lo K, Jenkins DJA, Boucher BA, Hanley AJ, Kendall CWC, et al. Relationship Between a Plant-Based Dietary Portfolio and Risk of Cardiovascular Disease: Findings From the Women's Health Initiative Prospective Cohort Study. J Am Heart Assoc. 2021;10(16):e021515.
77. Glenn AJ, Guasch-Ferré M, Malik VS, Kendall CWC, Manson JE, Rimm EB, et al. Portfolio Diet Score and Risk of Cardiovascular Disease: Findings From 3 Prospective Cohort Studies. Circulation. 2023;148(22):1750-63.
78. Golzarand M, Mirmiran P, Azizi F. Adherence to the MIND diet and the risk of cardiovascular disease in adults: a cohort study. Food Funct. 2022;13(3):1651-8.
79. Gómez-Donoso C, Martínez-González M, Perez-Cornago A, Sayón-Orea C, Martínez JA, Bes-Rastrollo M. Association between the nutrient profile system underpinning the Nutri-Score front-of-pack nutrition label and mortality in the SUN project: A prospective cohort study. Clin Nutr. 2021;40(3):1085-94.
80. Grau N, Mohammadifard N, Hassannejhad R, Haghighatdoost F, Sadeghi M, Talaei M, et al. Red and processed meat consumption and risk of incident cardiovascular disease and mortality: Isfahan cohort study. Int J Food Sci Nutr. 2022;73(4):503-12.
81. Gu X, Wang DD, Fung TT, Mozaffarian D, Djoussé L, Rosner B, et al. Dietary quality and risk of heart failure in men. Am J Clin Nutr. 2022;116(2):378-85.
82. Guasch-Ferré M, Zong G, Willett WC, Zock PL, Wanders AJ, Hu FB, et al. Associations of Monounsaturated Fatty Acids From Plant and Animal Sources With Total and Cause-Specific Mortality in Two US Prospective Cohort Studies. Circ Res. 2019;124(8):1266-75.
83. Guasch-Ferré M, Liu G, Li Y, Sampson L, Manson JE, Salas-Salvadó J, et al. Olive Oil Consumption and Cardiovascular Risk in U.S. Adults. J Am Coll Cardiol. 2020;75(15):1729-39.
84. Guasch-Ferré M, Li Y, Willett WC, Sun Q, Sampson L, Salas-Salvadó J, et al. Consumption of Olive Oil and Risk of Total and Cause-Specific Mortality Among U.S. Adults. J Am Coll Cardiol. 2022;79(2):101-12.
85. Hansen MD, Würtz AML, Hansen CP, Tjønneland A, Rimm EB, Johnsen SP, et al. Substitutions between potatoes and other vegetables and risk of ischemic stroke. Eur J Nutr. 2021;60(1):229-37.
86. Harbers MC, de Kroon AM, Boer JMA, Asselbergs FW, Geleijnse JM, Verschuren WMM, et al. Adherence to the Dutch dietary guidelines and 15-year incidence of heart failure in the EPIC-NL cohort. Eur J Nutr. 2020;59(8):3405-13.
87. Hashemian M, Farvid MS, Poustchi H, Murphy G, Etemadi A, Hekmatdoost A, et al. The application of six dietary scores to a Middle Eastern population: a comparative analysis of mortality in a prospective study. Eur J Epidemiol. 2019;34(4):371-82.

88. Hashemian M, Poustchi H, Murphy G, Etemadi A, Kamangar F, Pourshams A, et al. Turmeric, Pepper, Cinnamon, and Saffron Consumption and Mortality. J Am Heart Assoc. 2019;8(18):e012240.
89. Haugsgjerd TR, Egeland GM, Nygård OK, Vinknes KJ, Sulo G, Lysne V, et al. Association of dietary vitamin K and risk of coronary heart disease in middle-age adults: the Hordaland Health Study Cohort. BMJ Open. 2020;10(5):e035953.
90. Haugsgjerd TR, Egeland GM, Nygård OK, Igland J, Sulo G, Lysne V, et al. Intake of carbohydrates and SFA and risk of CHD in middle-age adults: the Hordaland Health Study (HUSK). Public Health Nutr. 2022;25(3):634-48.
91. Hejazi J, Ghanavati M, Hejazi E, Poustchi H, Sepanlou SG, Khoshnia M, et al. Habitual dietary intake of flavonoids and all-cause and cause-specific mortality: Golestan cohort study. Nutr J. 2020;19(1):108.
92. Hirahatake KM, Jiang L, Wong ND, Shikany JM, Eaton CB, Allison MA, et al. Diet Quality and Cardiovascular Disease Risk in Postmenopausal Women With Type 2 Diabetes Mellitus: The Women's Health Initiative. J Am Heart Assoc. 2019;8(19):e013249.
93. Hjelmgaard K, Bork CS, Lundbye-Christensen S, Lühdorf P, Bach FW, Schmidt EB, et al. Intake of Marine n-3 Polyunsaturated Fatty Acids and Risk of Hemorrhagic Stroke and Its Subtypes: A Danish Follow-Up Study. Cerebrovasc Dis. 2024;53(5):536-46.
94. Horikawa C, Kamada C, Tanaka S, Tanaka S, Araki A, Ito H, et al. Meat intake and incidence of cardiovascular disease in Japanese patients with type 2 diabetes: analysis of the Japan Diabetes Complications Study (JDCS). Eur J Nutr. 2019;58(1):281-90.
95. Horikawa C, Aida R, Tanaka S, Kamada C, Tanaka S, Yoshimura Y, et al. Sodium Intake and Incidence of Diabetes Complications in Elderly Patients with Type 2 Diabetes-Analysis of Data from the Japanese Elderly Diabetes Intervention Study (J-EDIT). Nutrients. 2021;13(2).
96. Hosseini-Esfahani F, Koochakpoor G, Tahmasebinejad Z, Khalili D, Mirmiran P, Azizi F. The association of dietary macronutrients composition with the incidence of cardiovascular disease, using iso-energetic substitution models: Tehran lipid and glucose study. Nutr Metab Cardiovasc Dis. 2020;30(12):2186-93.
97. Hu EA, Steffen LM, Coresh J, Appel LJ, Rebholz CM. Adherence to the Healthy Eating Index-2015 and Other Dietary Patterns May Reduce Risk of Cardiovascular Disease, Cardiovascular Mortality, and All-Cause Mortality. J Nutr. 2020;150(2):312-21.
98. Hu EA, Anderson CAM, Crews DC, Mills KT, He J, Shou H, et al. A Healthy Beverage Score and Risk of Chronic Kidney Disease Progression, Incident Cardiovascular Disease, and All-Cause Mortality in the Chronic Renal Insufficiency Cohort. Curr Dev Nutr. 2020;4(6):nzaa088.
99. Hu Y, Li Y, Sampson L, Wang M, Manson JE, Rimm E, et al. Lignan Intake and Risk of Coronary Heart Disease. J Am Coll Cardiol. 2021;78(7):666-78.

100. Hu Y, Willett WC, Manson JAE, Rosner B, Hu FB, Sun Q. Intake of whole grain foods and risk of coronary heart disease in US men and women. BMC Med. 2022;20(1):192.
101. Huang J, Liao LM, Weinstein SJ, Sinha R, Graubard BI, Albanes D. Association Between Plant and Animal Protein Intake and Overall and Cause-Specific Mortality. JAMA Intern Med. 2020;180(9):1173-84.
102. Huang HL, Abe SK, Sawada N, Takachi R, Ishihara J, Iwasaki M, et al. Association of sugary drink consumption with all-cause and cause-specific mortality: the Japan Public Health Center-based Prospective Study. Prev Med. 2021;148:106561.
103. Huang M, Lo K, Li J, Allison M, Wu WC, Liu S. Pasta meal intake in relation to risks of type 2 diabetes and atherosclerotic cardiovascular disease in postmenopausal women : findings from the Women's Health Initiative. BMJ Nutr Prev Health. 2021;4(1):195-205.
104. Ibsen DB, Christiansen AH, Olsen A, Tjønneland A, Overvad K, Wolk A, et al. Adherence to the EAT-Lancet Diet and Risk of Stroke and Stroke Subtypes: A Cohort Study. Stroke. 2022;53(1):154-63.
105. Ikehara S, Iso H, Kokubo Y, Yamagishi K, Saito I, Yatsuya H, et al. Peanut Consumption and Risk of Stroke and Ischemic Heart Disease in Japanese Men and Women: The JPHC Study. Stroke. 2021;52(11):3543-50.
106. Im J, Park K. Association between Soy Food and Dietary Soy Isoflavone Intake and the Risk of Cardiovascular Disease in Women: A Prospective Cohort Study in Korea. Nutrients. 2021;13(5).
107. Imran TF, Kim E, Buring JE, Lee IM, Gaziano JM, Djousse L. Nut consumption, risk of cardiovascular mortality, and potential mediating mechanisms: The Women's Health Study. J Clin Lipidol. 2021;15(2):266-74.
108. Ivey KL, Nguyen XT, Quaden RM, Ho YL, Cho K, Gaziano JM, et al. Association of Nut Consumption with Risk of Stroke and Cardiovascular Disease: The Million Veteran Program. Nutrients. 2021;13(9).
109. Jackson JK, Zong G, MacDonald-Wicks LK, Patterson AJ, Willett WC, Rimm EB, et al. Dietary nitrate consumption and risk of CHD in women from the Nurses' Health Study. Br J Nutr. 2019;121(7):831-8.
110. Jackson JK, Patterson AJ, MacDonald-Wicks LK, Forder PM, Blekkenhorst LC, Bondonno CP, et al. Vegetable Nitrate Intakes Are Associated with Reduced Self-Reported Cardiovascular-Related Complications within a Representative Sample of Middle-Aged Australian Women, Prospectively Followed up for 15 Years. Nutrients. 2019;11(2).
111. Jackson JK, MacDonald-Wicks LK, McEvoy MA, Forder PM, Holder C, Oldmeadow C, et al. Better diet quality scores are associated with a lower risk of hypertension and non-fatal CVD in middle-aged Australian women over 15 years of follow-up. Public Health Nutr. 2020;23(5):882-93.

112. Jalali M, Bahadoran Z, Mirmiran P, Khalili D, Symonds ME, Azizi F, et al. Higher ultra-processed food intake is associated with an increased incidence risk of cardiovascular disease: the Tehran lipid and glucose study. Nutr Metab (Lond). 2024;21(1):14.
113. Jeon J, Park K. Dietary Vitamin B(6) Intake Associated with a Decreased Risk of Cardiovascular Disease: A Prospective Cohort Study. Nutrients. 2019;11(7).
114. Jeong J, Lim K, Shin S. The association between meat intake and the risk of coronary heart disease in Korean men using the Framingham risk score: A prospective cohort study. Nutr Metab Cardiovasc Dis. 2023;33(6):1158-66.
115. Jo U, Park K. Carbohydrate-based diet may increase the risk of cardiovascular disease: A pooled analysis of two prospective cohort studies. Clin Nutr. 2023;42(8):1301-7.
116. Johansson I, Esberg A, Nilsson LM, Jansson JH, Wennberg P, Winkvist A. Dairy Product Intake and Cardiometabolic Diseases in Northern Sweden: A 33-Year Prospective Cohort Study. Nutrients. 2019;11(2).
117. Johansson A, Acosta S. Diet and Lifestyle as Risk Factors for Carotid Artery Disease: A Prospective Cohort Study. Cerebrovasc Dis. 2020;49(5):563-9.
118. Jung H, Shin J, Lim K, Shin S. Edible mushroom intake and risk of all-cause and cause-specific mortality: results from the Korean Genome and Epidemiology Study (KoGES) Cohort. Food Funct. 2023;14(19):8829-37.
119. Juul F, Vaidean G, Lin Y, Deierlein AL, Parekh N. Ultra-Processed Foods and Incident Cardiovascular Disease in the Framingham Offspring Study. J Am Coll Cardiol. 2021;77(12):1520-31.
120. Kang M, Park SY, Boushey CJ, Wilkens LR, Le Marchand L, Hankin JH, et al. Does Incorporating Gender Differences into Quantifying a Food Frequency Questionnaire Influence the Association of Total Energy Intake with All-Cause and Cause-Specific Mortality? Nutrients. 2020;12(10).
121. Kashino I, Mizoue T, Serafini M, Akter S, Sawada N, Ishihara J, et al. Higher Dietary Non-enzymatic Antioxidant Capacity Is Associated with Decreased Risk of All-Cause and Cardiovascular Disease Mortality in Japanese Adults. J Nutr. 2019.
122. Katagiri R, Sawada N, Goto A, Yamaji T, Iwasaki M, Noda M, et al. Association of soy and fermented soy product intake with total and cause specific mortality: prospective cohort study. Bmj. 2020;368:m34.
123. Katagiri R, Goto A, Sawada N, Yamaji T, Iwasaki M, Noda M, et al. Dietary fiber intake and total and cause-specific mortality: the Japan Public Health Center-based prospective study. Am J Clin Nutr. 2020;111(5):1027-35.
124. Kazemi A, Sasani N, Mokhtari Z, Keshtkar A, Babajafari S, Poustchi H, et al. Comparing the risk of cardiovascular diseases and all-cause mortality in four lifestyles with a combination of high/low physical activity and healthy/unhealthy diet: a prospective cohort study. Int J Behav Nutr Phys Act. 2022;19(1):138.

125. Keller A, O'Reilly EJ, Malik V, Buring JE, Andersen I, Steffen L, et al. Substitution of sugar-sweetened beverages for other beverages and the risk of developing coronary heart disease: Results from the Harvard Pooling Project of Diet and Coronary Disease. Prev Med. 2020;131:105970.
126. Kermani-Alghoraishi M, Behrouzi A, Hassannejad R, Sarrafzadegan N, Nouri F, Boshatam M, et al. Ultra-processed food consumption and cardiovascular events rate: An analysis from Isfahan Cohort Study (ICS). Nutr Metab Cardiovasc Dis. 2024;34(6):1438-47.
127. Khan I, Kwon M, Shivappa N, Hébert JR, Kim MK. Positive Association of Dietary Inflammatory Index with Incidence of Cardiovascular Disease: Findings from a Korean Population-Based Prospective Study. Nutrients. 2020;12(2).
128. Kim J, Kim H, Giovannucci EL. Plant-based diet quality and the risk of total and disease-specific mortality: A population-based prospective study. Clin Nutr. 2021;40(12):5718-25.
129. Kityo A, Lee SA. The intake of ultra-processed foods, all-cause, cancer and cardiovascular mortality in the Korean Genome and Epidemiology Study-Health Examinees (KoGES-HEXA) cohort. PLoS One. 2023;18(5):e0285314.
130. Kjeldsen EW, Thomassen JQ, Rasmussen KL, Nordestgaard BG, Tybjærg-Hansen A, Frikke-Schmidt R. Impact of diet on ten-year absolute cardiovascular risk in a prospective cohort of 94 321 individuals: A tool for implementation of healthy diets. Lancet Reg Health Eur. 2022;19:100419.
131. Kouvari M, Panagiotakos DB, Chrysohoou C, Georgousopoulou EN, Yannakoulia M, Tousoulis D, et al. Dairy products, surrogate markers, and cardiovascular disease; a sex-specific analysis from the ATTICA prospective study. Nutr Metab Cardiovasc Dis. 2020;30(12):2194-206.
132. Kouvari M, Panagiotakos DB, Chrysohoou C, Yannakoulia M, Georgousopoulou EN, Tousoulis D, et al. Dietary vitamin D intake, cardiovascular disease and cardiometabolic risk factors: a sex-based analysis from the ATTICA cohort study. J Hum Nutr Diet. 2020;33(5):708-17.
133. Kouvari M, Tsiampalis T, Chrysohoou C, Georgousopoulou E, Skoumas J, Mantzoros CS, et al. Quality of plant-based diets in relation to 10-year cardiovascular disease risk: the ATTICA cohort study. Eur J Nutr. 2022;61(5):2639-49.
134. Kvist K, Laursen ASD, Overvad K, Jakobsen MU. Substitution of Milk with Whole-Fat Yogurt Products or Cheese Is Associated with a Lower Risk of Myocardial Infarction: The Danish Diet, Cancer and Health cohort. J Nutr. 2020;150(5):1252-8.
135. Kwon YJ, Lee HS, Park G, Kim HM, Lee JW. Association of Dietary Fiber Intake with All-Cause Mortality and Cardiovascular Disease Mortality: A 10-Year Prospective Cohort Study. Nutrients. 2022;14(15).
136. Kwon YJ, Lee HS, Park G, Yang J, Kim HM, Lee JW. Dietary Zinc Intake and All-Cause and Cardiovascular Mortality in Korean Middle-Aged and Older Adults. Nutrients. 2023;15(2).

137. Langsetmo L, Harrison S, Jonnalagadda S, Pereira SL, Shikany JM, Farsijani S, et al. Low Protein Intake Irrespective of Source is Associated with Higher Mortality Among Older Community-dwelling Men. J Nutr Health Aging. 2020;24(8):900-5.
138. Lara KM, Levitan EB, Gutierrez OM, Shikany JM, Safford MM, Judd SE, et al. Dietary Patterns and Incident Heart Failure in U.S. Adults Without Known Coronary Disease. J Am Coll Cardiol. 2019;73(16):2036-45.
139. Laursen ASD, Sluijs I, Boer JMA, Verschuren WMM, van der Schouw YT, Jakobsen MU. Substitutions between dairy products and risk of stroke: results from the European Investigation into Cancer and Nutrition-Netherlands (EPIC-NL) cohort. Br J Nutr. 2019;121(12):1398-404.
140. Lee DH, Yang M, Giovannucci EL, Sun Q, Chavarro JE. Mushroom consumption, biomarkers, and risk of cardiovascular disease and type 2 diabetes: a prospective cohort study of US women and men. Am J Clin Nutr. 2019;110(3):666-74.
141. Li J, Lee DH, Hu J, Tabung FK, Li Y, Bhupathiraju SN, et al. Dietary Inflammatory Potential and Risk of Cardiovascular Disease Among Men and Women in the U.S. J Am Coll Cardiol. 2020;76(19):2181-93.
142. Li J, Hovey KM, Andrews CA, Quddus A, Allison MA, Van Horn L, et al. Association of Dietary Magnesium Intake with Fatal Coronary Heart Disease and Sudden Cardiac Death. J Womens Health (Larchmt). 2020;29(1):7-12.
143. Li Z, Gao Y, Byrd DA, Gibbs DC, Prizment AE, Lazovich D, et al. Novel Dietary and Lifestyle Inflammation Scores Directly Associated with All-Cause, All-Cancer, and All-Cardiovascular Disease Mortality Risks Among Women. J Nutr. 2021;151(4):930-9.
144. Li X, Dehghan M, Tse LA, Lang X, Rangarajan S, Liu W, et al. Associations of dietary copper intake with cardiovascular disease and mortality: findings from the Chinese Perspective Urban and Rural Epidemiology (PURE-China) Study. BMC Public Health. 2023;23(1):2525.
145. Li Q, Chang M, Lai R, Zhang H, Song L, Wang X, et al. Potential benefits of spicy food consumption on cardiovascular outcomes in patients with diabetes: A cohort study of the China Kadoorie Biobank. Nutrition. 2023;112:112062.
146. Liang Z, Feng Y, Shivappa N, Hebert JR, Xu X. Dietary Inflammatory Index and Mortality from All Causes, Cardiovascular Disease, and Cancer: A Prospective Study. Cancers (Basel). 2022;14(19).
147. Lilja E, Bergwall S, Sonestedt E, Gottsäter A, Acosta S. The association between dietary intake, lifestyle and incident symptomatic peripheral arterial disease among individuals with diabetes mellitus: insights from the Malmö Diet and Cancer study. Ther Adv Endocrinol Metab. 2019;10:2042018819890532.
148. Lim CGY, Tai ES, van Dam RM. Replacing dietary carbohydrates and refined grains with different alternatives and risk of cardiovascular diseases in a multi-ethnic Asian population. Am J Clin Nutr. 2022;115(3):854-63.

149. Liu G, Guasch-Ferré M, Hu Y, Li Y, Hu FB, Rimm EB, et al. Nut Consumption in Relation to Cardiovascular Disease Incidence and Mortality Among Patients With Diabetes Mellitus. Circ Res. 2019;124(6):920-9.
150. Liu AH, Bondonno CP, Russell J, Flood VM, Lewis JR, Croft KD, et al. Relationship of dietary nitrate intake from vegetables with cardiovascular disease mortality: a prospective study in a cohort of older Australians. Eur J Nutr. 2019;58(7):2741-53.
151. Liu X, Guasch-Ferré M, Drouin-Chartier JP, Tobias DK, Bhupathiraju SN, Rexrode KM, et al. Changes in Nut Consumption and Subsequent Cardiovascular Disease Risk Among US Men and Women: 3 Large Prospective Cohort Studies. J Am Heart Assoc. 2020;9(7):e013877.
152. Liu W, Hu B, Dehghan M, Mente A, Wang C, Yan R, et al. Fruit, vegetable, and legume intake and the risk of all-cause, cardiovascular, and cancer mortality: A prospective study. Clin Nutr. 2021;40(6):4316-23.
153. Liu S, Wang D, Li B, Li K, Dai X, Cheng L, et al. Dietary betaine intake and risk of mortality in patients with coronary artery disease: the prospective Guangdong Coronary Artery Disease Cohort. Br J Nutr. 2023;130(1):10-9.
154. Liu X, Hu Y, Chen L, Luo Y, Tang W, Liu X, et al. Effect of health lifestyle on the risk of stroke: A prospective cohort study from Chongqing, China. J Stroke Cerebrovasc Dis. 2024;33(9):107846.
155. Liu D, Tan S, Zhou Z, Gu S, Zuo H. Trimethylamine N-oxide, β-alanine, tryptophan index, and vitamin B6-related dietary patterns in association with stroke risk. Nutrition, Metabolism and Cardiovascular Diseases. 2024;34(5):1179-88.
156. Livingstone KM, Milte CM, Torres SJ, Hart MJ, Dingle SE, Shaw JE, et al. Nineteen-Year Associations between Three Diet Quality Indices and All-Cause and Cardiovascular Disease Mortality: The Australian Diabetes, Obesity, and Lifestyle Study. J Nutr. 2022;152(3):805-15.
157. Lo K, Glenn AJ, Yeung S, Kendall CWC, Sievenpiper JL, Jenkins DJA, et al. Prospective Association of the Portfolio Diet with All-Cause and Cause-Specific Mortality Risk in the Mr. OS and Ms. OS Study. Nutrients. 2021;13(12).
158. Lukic M, Barnung RB, Skeie G, Olsen KS, Braaten T. Coffee consumption and overall and cause-specific mortality: the Norwegian Women and Cancer Study (NOWAC). Eur J Epidemiol. 2020;35(10):913-24.
159. Luong R, Ribeiro RV, Rangan A, Naganathan V, Blyth F, Waite LM, et al. Haem Iron Intake Is Associated with Increased Major Adverse Cardiovascular Events, All-Cause Mortality, Congestive Cardiac Failure, and Coronary Revascularisation in Older Men: The Concord Health and Ageing in Men Project. J Nutr Health Aging. 2023;27(7):559-70.
160. Luong R, Ribeiro R, Naganathan V, Blyth F, Waite LM, Handelsman DJ, et al. Empirically derived dietary patterns are associated with major adverse cardiovascular events, all-cause mortality, and congestive cardiac failure in older men: The Concord Health and Ageing in Men Project. J Nutr Health Aging. 2024;28(2):100020.

161. Ma L, Liu G, Ding M, Zong G, Hu FB, Willett WC, et al. Isoflavone Intake and the Risk of Coronary Heart Disease in US Men and Women: Results From 3 Prospective Cohort Studies. Circulation. 2020;141(14):1127-37.
162. Ma L, Hu Y, Alperet DJ, Liu G, Malik V, Manson JE, et al. Beverage consumption and mortality among adults with type 2 diabetes: prospective cohort study. Bmj. 2023;381:e073406.
163. Malik VS, Li Y, Pan A, De Koning L, Schernhammer E, Willett WC, et al. Long-Term Consumption of Sugar-Sweetened and Artificially Sweetened Beverages and Risk of Mortality in US Adults. Circulation. 2019;139(18):2113-25.
164. Mao Z, Troeschel AN, Judd SE, Shikany JM, Levitan EB, Safford MM, et al. Association of an evolutionary-concordance lifestyle pattern score with incident CVD among Black and White men and women. Br J Nutr. 2022:1-10.
165. Matre Å O, Van Parys A, Olsen T, Haugsgjerd TR, Baravelli CM, Nygård O, et al. The Association of Meat Intake With All-Cause Mortality and Acute Myocardial Infarction Is Age-Dependent in Patients With Stable Angina Pectoris. Front Nutr. 2021;8:642612.
166. Matsuyama S, Sawada N, Tomata Y, Zhang S, Goto A, Yamaji T, et al. Association between adherence to the Japanese diet and all-cause and cause-specific mortality: the Japan Public Health Center-based Prospective Study. Eur J Nutr. 2021;60(3):1327-36.
167. Mendonça RD, Carvalho NC, Martin-Moreno JM, Pimenta AM, Lopes ACS, Gea A, et al. Total polyphenol intake, polyphenol subtypes and incidence of cardiovascular disease: The SUN cohort study. Nutr Metab Cardiovasc Dis. 2019;29(1):69-78.
168. Meng G, Liu T, Rayamajhi S, Thapa A, Zhang S, Wang X, et al. Association between soft drink consumption and carotid atherosclerosis in a large-scale adult population: The TCLSIH cohort study. Nutr Metab Cardiovasc Dis. 2023;33(11):2209-19.
169. Meng G, Yao J, Li J, Gu Y, Wu H, Zhang Q, et al. Association between whole-grain consumption and carotid atherosclerosis: the Tianjin chronic low-grade systemic inflammation and health (TCLSIH) cohort study. Food Funct. 2023;14(24):10955-63.
170. Michaëlsson K, Baron JA, Byberg L, Höijer J, Larsson SC, Svennblad B, et al. Combined associations of body mass index and adherence to a Mediterranean-like diet with all-cause and cardiovascular mortality: A cohort study. PLoS Med. 2020;17(9):e1003331.
171. Mirmiran P, Houshialsadat Z, Bahadoran Z, Khalili-Moghadam S, Sheikholeslami F, Azizi F. Association of dietary fatty acids and the incidence risk of cardiovascular disease in adults: the Tehran Lipid and Glucose Prospective Study. BMC Public Health. 2020;20(1):1743.
172. Mirmiran P, Estaki S, Yadegari A, Golzarand M, Azizi F. Adherence to a modified nordic diet and the risk of cardiovascular events in a non-nordic population: a prospective cohort study. Eur J Clin Nutr. 2023;77(9):919-24.
173. Mohammadifard N, Ghaderian N, Hassannejad R, Sajjadi F, Sadeghi M, Roohafza H, et al. Longitudinal Association of Nut Consumption and the Risk of Cardiovascular Events: A Prospective Cohort Study in the Eastern Mediterranean Region. Front Nutr. 2020;7:610467.

174. Mohammadifard N, Taheri M, Haghighatdoost F, Grau N, Najafian J, Sadeghi M, et al. Egg consumption and risk of cardiovascular events among Iranians: results from Isfahan Cohort Study (ICS). Eur J Clin Nutr. 2022;76(10):1409-14.
175. Mohan D, Mente A, Dehghan M, Rangarajan S, O'Donnell M, Hu W, et al. Associations of Fish Consumption With Risk of Cardiovascular Disease and Mortality Among Individuals With or Without Vascular Disease From 58 Countries. JAMA Intern Med. 2021;181(5):631-49.
176. Mohseni M, Mohammadifard N, Hassannejad R, Aghabozorgi M, Shirani F, Sadeghi M, et al. Longitudinal association of dietary habits and the risk of cardiovascular disease among Iranian population between 2001 and 2013: the Isfahan Cohort Study. Sci Rep. 2023;13(1):5364.
177. Mori N, Shimazu T, Charvat H, Mutoh M, Sawada N, Iwasaki M, et al. Cruciferous vegetable intake and mortality in middle-aged adults: A prospective cohort study. Clin Nutr. 2019;38(2):631-43.
178. Mosallanezhad Z, Jalali M, Bahadoran Z, Mirmiran P, Azizi F. Dietary sodium to potassium ratio is an independent predictor of cardiovascular events: a longitudinal follow-up study. BMC Public Health. 2023;23(1):705.
179. Murai U, Yamagishi K, Sata M, Kokubo Y, Saito I, Yatsuya H, et al. Seaweed intake and risk of cardiovascular disease: the Japan Public Health Center-based Prospective (JPHC) Study. Am J Clin Nutr. 2019;110(6):1449-55.
180. Musicus AA, Wang DD, Janiszewski M, Eshel G, Blondin SA, Willett W, et al. Health and environmental impacts of plant-rich dietary patterns: a US prospective cohort study. Lancet Planet Health. 2022;6(11):e892-e900.
181. Nanri A, Mizoue T, Goto A, Noda M, Sawada N, Tsugane S. Vitamin D intake and all-cause and cause-specific mortality in Japanese men and women: the Japan Public Health Center-based prospective study. Eur J Epidemiol. 2023;38(3):291-300.
182. Norouzzadeh M, Teymoori F, Farhadnejad H, Moslehi N, Rahideh ST, Mirmiran P, et al. The interaction between diet quality and cigarette smoking on the incidence of hypertension, stroke, cardiovascular diseases, and all-cause mortality. Sci Rep. 2024;14(1):12371.
183. Nozue M, Shimazu T, Charvat H, Mori N, Mutoh M, Sawada N, et al. Fermented soy products intake and risk of cardiovascular disease and total cancer incidence: The Japan Public Health Center-based Prospective study. Eur J Clin Nutr. 2021;75(6):954-68.
184. Oh SW, Wood AC, Hwang SS, Allison M. Racial and Ethnic Differences in the Association of Low-Carbohydrate Diet With Mortality in the Multi-Ethnic Study of Atherosclerosis. JAMA Netw Open. 2022;5(10):e2237552.
185. Okada E, Shirakawa T, Shivappa N, Wakai K, Suzuki K, Date C, et al. Dietary Inflammatory Index Is Associated with Risk of All-Cause and Cardiovascular Disease Mortality but Not with Cancer Mortality in Middle-Aged and Older Japanese Adults. J Nutr. 2019;149(8):1451-9.

186. Pacheco LS, Lacey JV, Jr., Martinez ME, Lemus H, Araneta MRG, Sears DD, et al. Sugar-Sweetened Beverage Intake and Cardiovascular Disease Risk in the California Teachers Study. J Am Heart Assoc. 2020;9(10):e014883.
187. Pacheco LS, Lacey JV, Jr., Martinez ME, Lemus H, Sears DD, Araneta MRG, et al. Association Between Sugar-Sweetened Beverage Intake and Mortality Risk in Women: The California Teachers Study. J Acad Nutr Diet. 2022;122(2):320-33.e6.
188. Pacheco LS, Li Y, Rimm EB, Manson JE, Sun Q, Rexrode K, et al. Avocado Consumption and Risk of Cardiovascular Disease in US Adults. J Am Heart Assoc. 2022;11(7):e024014.
189. Palmer CR, Bellinge JW, Dalgaard F, Sim M, Murray K, Connolly E, et al. Association between vitamin K(1) intake and mortality in the Danish Diet, Cancer, and Health cohort. Eur J Epidemiol. 2021;36(10):1005-14.
190. Pan XF, Yang JJ, Lipworth LP, Shu XO, Cai H, Steinwandel MD, et al. Cholesterol and Egg Intakes with Cardiometabolic and All-Cause Mortality among Chinese and Low-Income Black and White Americans. Nutrients. 2021;13(6).
191. Panagiotakos DB, Kouli GM, Magriplis E, Kyrou I, Georgousopoulou EN, Chrysohoou C, et al. Beer, wine consumption, and 10-year CVD incidence: the ATTICA study. Eur J Clin Nutr. 2019;73(7):1015-23.
192. Papandreou C, Becerra-Tomás N, Bulló M, Martínez-González M, Corella D, Estruch R, et al. Legume consumption and risk of all-cause, cardiovascular, and cancer mortality in the PREDIMED study. Clin Nutr. 2019;38(1):348-56.
193. Parmenter BH, Dalgaard F, Murray K, Cassidy A, Bondonno CP, Lewis JR, et al. Habitual flavonoid intake and ischemic stroke incidence in the Danish Diet, Cancer, and Health Cohort. Am J Clin Nutr. 2021;114(1):348-57.
194. Parmenter BH, Pokharel P, Dalgaard F, Murray K, Cassidy A, Bondonno CP, et al. Higher Habitual Dietary Intakes of Flavanols and Anthocyanins Differentially Associate with Lower Incidence of Ischemic Stroke Subtypes-A Follow-Up Analysis. J Nutr. 2023;153(11):3280-6.
195. Parmenter BH, Dalgaard F, Murray K, Marquis-Gravel G, Cassidy A, Bondonno CP, et al. Intake of dietary flavonoids and incidence of ischemic heart disease in the Danish Diet, Cancer, and Health cohort. Eur J Clin Nutr. 2023;77(2):270-7.
196. Patel YR, Robbins JM, Gaziano JM, Djoussé L. Mediterranean, DASH, and Alternate Healthy Eating Index Dietary Patterns and Risk of Death in the Physicians' Health Study. Nutrients. 2021;13(6).
197. Pertiwi K, Küpers LK, de Goede J, Zock PL, Kromhout D, Geleijnse JM. Dietary and Circulating Long-Chain Omega-3 Polyunsaturated Fatty Acids and Mortality Risk After Myocardial Infarction: A Long-Term Follow-Up of the Alpha Omega Cohort. J Am Heart Assoc. 2021;10(23):e022617.

198. Praagman J, Vissers LET, Mulligan AA, Laursen ASD, Beulens JWJ, van der Schouw YT, et al. Consumption of individual saturated fatty acids and the risk of myocardial infarction in a UK and a Danish cohort. Int J Cardiol. 2019;279:18-26.
199. Ruggiero E, Di Castelnuovo A, Costanzo S, Persichillo M, De Curtis A, Cerletti C, et al. Daily Coffee Drinking Is Associated with Lower Risks of Cardiovascular and Total Mortality in a General Italian Population: Results from the Moli-sani Study. J Nutr. 2021;151(2):395-404.
200. Ruggiero E, Di Castelnuovo A, Costanzo S, Persichillo M, De Curtis A, Cerletti C, et al. Egg consumption and risk of all-cause and cause-specific mortality in an Italian adult population. Eur J Nutr. 2021;60(7):3691-702.
201. Ruggiero E, Di Castelnuovo A, Costanzo S, Esposito S, De Curtis A, Persichillo M, et al. Olive oil consumption is associated with lower cancer, cardiovascular and all-cause mortality among Italian adults: prospective results from the Moli-sani Study and analysis of potential biological mechanisms. Eur J Clin Nutr. 2024;78(8):684-93.
202. Sadeghi M, Simani M, Mohammadifard N, Talaei M, Roohafza H, Hassannejad R, et al. Longitudinal association of dietary fat intake with cardiovascular events in a prospective cohort study in Eastern Mediterranean region. Int J Food Sci Nutr. 2021;72(8):1095-104.
203. Saglimbene VM, Wong G, Ruospo M, Palmer SC, Campbell K, Larsen VG, et al. Dietary n-3 polyunsaturated fatty acid intake and all-cause and cardiovascular mortality in adults on hemodialysis: The DIET-HD multinational cohort study. Clin Nutr. 2019;38(1):429-37.
204. Saglimbene VM, Wong G, Ruospo M, Palmer SC, Garcia-Larsen V, Natale P, et al. Fruit and Vegetable Intake and Mortality in Adults undergoing Maintenance Hemodialysis. Clin J Am Soc Nephrol. 2019;14(2):250-60.
205. Saglimbene VM, Wong G, Teixeira-Pinto A, Ruospo M, Garcia-Larsen V, Palmer SC, et al. Dietary Patterns and Mortality in a Multinational Cohort of Adults Receiving Hemodialysis. Am J Kidney Dis. 2020;75(3):361-72.
206. Sahashi Y, Goto A, Takachi R, Ishihara J, Kito K, Kanehara R, et al. Inverse Association between Fruit and Vegetable Intake and All-Cause Mortality: Japan Public Health Center-Based Prospective Study. J Nutr. 2022;152(10):2245-54.
207. Saito E, Tang X, Abe SK, Sawada N, Ishihara J, Takachi R, et al. Association between meat intake and mortality due to all-cause and major causes of death in a Japanese population. PLoS One. 2020;15(12):e0244007.
208. Sakamaki T, Kayaba K, Kotani K, Namekawa M, Hamaguchi T, Nakaya N, et al. Coffee consumption and mortality in Japan with 18 years of follow-up: the Jichi Medical School Cohort Study. Public Health. 2021;191:23-30.
209. Sawicki CM, Ramesh G, Bui L, Nair NK, Hu FB, Rimm EB, et al. Planetary health diet and cardiovascular disease: results from three large prospective cohort studies in the USA. Lancet Planet Health. 2024;8(9):e666-e74.

210. Scheffers FR, Boer JMA, Verschuren WMM, Verheus M, van der Schouw YT, Sluijs I, et al. Pure fruit juice and fruit consumption and the risk of CVD: the European Prospective Investigation into Cancer and Nutrition-Netherlands (EPIC-NL) study. Br J Nutr. 2019;121(3):351-9.
211. Schmidt AB, Lund M, Corn G, Halldorsson TI, Øyen N, Wohlfahrt J, et al. Dietary glycemic index and glycemic load during pregnancy and offspring risk of congenital heart defects: a prospective cohort study. Am J Clin Nutr. 2020;111(3):526-35.
212. Shams-White MM, Brockton NT, Mitrou P, Kahle LL, Reedy J. The 2018 World Cancer Research Fund/American Institute for Cancer Research (WCRF/AICR) Score and All-Cause, Cancer, and Cardiovascular Disease Mortality Risk: A Longitudinal Analysis in the NIH-AARP Diet and Health Study. Curr Dev Nutr. 2022;6(6):nzac096.
213. Shan Z, Li Y, Baden MY, Bhupathiraju SN, Wang DD, Sun Q, et al. Association Between Healthy Eating Patterns and Risk of Cardiovascular Disease. JAMA Intern Med. 2020;180(8):1090-100.
214. Shao MY, Jiang CQ, Zhang WS, Zhu F, Jin YL, Woo J, et al. Association of fish consumption with risk of all-cause and cardiovascular disease mortality: an 11-year follow-up of the Guangzhou Biobank Cohort Study. Eur J Clin Nutr. 2022;76(3):389-96.
215. Sheng LT, Jiang YW, Pan A, Koh WP. Dietary total antioxidant capacity and mortality outcomes: the Singapore Chinese Health Study. Eur J Nutr. 2022;61(5):2375-82.
216. Shikany JM, Safford MM, Soroka O, Brown TM, Newby PK, Durant RW, et al. Mediterranean Diet Score, Dietary Patterns, and Risk of Sudden Cardiac Death in the REGARDS Study. Journal of the American Heart Association. 2021;10(13):e019158.
217. Shin S, Lee JE, Loftfield E, Shu XO, Abe SK, Rahman MS, et al. Coffee and tea consumption and mortality from all causes, cardiovascular disease and cancer: a pooled analysis of prospective studies from the Asia Cohort Consortium. Int J Epidemiol. 2022;51(2):626-40.
218. Silva FM, Giatti L, Diniz M, Brant LCC, Barreto SM. Dairy product consumption reduces cardiovascular mortality: results after 8 year follow-up of ELSA-Brasil. Eur J Nutr. 2022;61(2):859-69.
219. Sjöblom L, Hantikainen E, Bellocco R, Ye W, Adami HO, Trolle Lagerros Y, et al. Nordic Nutrition Recommendations and risk of myocardial infarction and stroke: a prospective cohort study. Eur J Nutr. 2024;63(4):1151-62.
220. Son GH, Lee HS, Kwon YJ, Lee JW. Association between carbohydrate to protein or fat ratio and mortality: A prospective cohort study. Clin Nutr ESPEN. 2024;63:805-12.
221. Son J, Lee Y, Park K. Effects of processed red meat consumption on the risk of type 2 diabetes and cardiovascular diseases among Korean adults: the Korean Genome and Epidemiology Study. Eur J Nutr. 2019;58(6):2477-84.

222. Stefler D, Brett D, Sarkadi-Nagy E, Kopczynska E, Detchev S, Bati A, et al. Traditional Eastern European diet and mortality: prospective evidence from the HAPIEE study. Eur J Nutr. 2021;60(2):1091-100.
223. Strengers JG, den Ruijter HM, Boer JMA, Asselbergs FW, Verschuren WMM, van der Schouw YT, et al. The association of the Mediterranean diet with heart failure risk in a Dutch population. Nutr Metab Cardiovasc Dis. 2021;31(1):60-6.
224. Su J, Geng H, Chen L, Fan X, Zhou J, Wu M, et al. Association of healthy lifestyle with incident cardiovascular diseases among hypertensive and normotensive Chinese adults. Front Cardiovasc Med. 2023;10:1046943.
225. Sun Y, Liu B, Snetselaar LG, Robinson JG, Wallace RB, Peterson LL, et al. Association of fried food consumption with all cause, cardiovascular, and cancer mortality: prospective cohort study. Bmj. 2019;364:k5420.
226. Sun C, Zhang WS, Jiang CQ, Jin YL, Zhu T, Zhu F, et al. Quantity and Variety in Fruit and Vegetable Consumption and Mortality in Older Chinese: A 15-year Follow-Up of a Prospective Cohort Study. J Nutr. 2023;153(7):2061-72.
227. Sun C, Zhang WS, Jiang CQ, Jin YL, Au Yeung SL, Woo J, et al. Association of Cantonese dietary patterns with mortality risk in older Chinese: a 16-year follow-up of a Guangzhou Biobank cohort study. Food Funct. 2024;15(8):4538-51.
228. Swaminathan S, Dehghan M, Raj JM, Thomas T, Rangarajan S, Jenkins D, et al. Associations of cereal grains intake with cardiovascular disease and mortality across 21 countries in Prospective Urban and Rural Epidemiology study: prospective cohort study. Bmj. 2021;372:m4948.
229. Taguchi C, Kishimoto Y, Fukushima Y, Kondo K, Yamakawa M, Wada K, et al. Dietary intake of total polyphenols and the risk of all-cause and specific-cause mortality in Japanese adults: the Takayama study. Eur J Nutr. 2020;59(3):1263-71.
230. Talaei M, Koh WP, Yuan JM, van Dam RM. DASH Dietary Pattern, Mediation by Mineral Intakes, and the Risk of Coronary Artery Disease and Stroke Mortality. J Am Heart Assoc. 2019;8(5):e011054.
231. Talaei M, Hosseini N, van Dam RM, Sadeghi M, Oveisgharan S, Dianatkhah M, et al. Whole milk consumption and risk of cardiovascular disease and mortality: Isfahan Cohort Study. Eur J Nutr. 2019;58(1):163-71.
232. Tamura T, Wakai K, Kato Y, Tamada Y, Kubo Y, Okada R, et al. Dietary Carbohydrate and Fat Intakes and Risk of Mortality in the Japanese Population: the Japan Multi-Institutional Collaborative Cohort Study. J Nutr. 2023;153(8):2352-68.
233. Tang J, Dong JY, Eshak ES, Cui R, Shirai K, Liu K, et al. Breakfast Type and Cardiovascular Mortality: The Japan Collaborative Cohort Study. J Atheroscler Thromb. 2023;30(9):1255-64.

234. Tanno K, Yonekura Y, Okuda N, Kuribayashi T, Yabe E, Tsubota-Utsugi M, et al. Association between Milk Intake and Incident Stroke among Japanese Community Dwellers: The Iwate-KENCO Study. Nutrients. 2021;13(11).
235. Tong TYN, Clarke R, Schmidt JA, Huybrechts I, Noor U, Forouhi NG, et al. Dietary amino acids and risk of stroke subtypes: a prospective analysis of 356,000 participants in seven European countries. Eur J Nutr. 2024;63(1):209-20.
236. Torres-Collado L, Compañ-Gabucio LM, González-Palacios S, Notario-Barandiaran L, Oncina-Cánovas A, Vioque J, et al. Coffee Consumption and All-Cause, Cardiovascular, and Cancer Mortality in an Adult Mediterranean Population. Nutrients. 2021;13(4).
237. Torres-Collado L, García-de la Hera M, Cano-Ibañez N, Bueno-Cavanillas A, Vioque J. Association between Dietary Diversity and All-Cause Mortality: A Multivariable Model in a Mediterranean Population with 18 Years of Follow-Up. Nutrients. 2022;14(8).
238. Troeschel AN, Hartman TJ, Flanders WD, Akinyemiju T, Judd S, Bostick RM. A novel evolutionary-concordance lifestyle score is inversely associated with all-cause, all-cancer, and all-cardiovascular disease mortality risk. Eur J Nutr. 2021;60(6):3485-97.
239. Troeschel AN, Byrd DA, Judd S, Flanders WD, Bostick RM. Associations of dietary and lifestyle inflammation scores with mortality due to CVD, cancer, and all causes among Black and White American men and women. Br J Nutr. 2023;129(3):523-34.
240. Um CY, Prizment A, Hong CP, Lazovich D, Bostick RM. Associations of calcium and dairy product intakes with all-cause, all-cancer, colorectal cancer and CHD mortality among older women in the Iowa Women's Health Study. Br J Nutr. 2019;121(10):1188-200.
241. Ushula TW, Mamun A, Darssan D, Wang WYS, Williams GM, Whiting SJ, et al. Dietary patterns explaining variations in blood biomarkers in young adults are associated with the 30-year predicted cardiovascular disease risks in midlife: A follow-up study. Nutr Metab Cardiovasc Dis. 2023;33(5):1007-18.
242. Van Parys A, Lysne V, Svingen GFT, Ueland PM, Dhar I, Øyen J, et al. Dietary choline is related to increased risk of acute myocardial infarction in patients with stable angina pectoris. Biochimie. 2020;173:68-75.
243. Vanegas P, Zazpe I, Santiago S, Fernandez-Lazaro CI, de la OV, Martínez-González M. Macronutrient quality index and cardiovascular disease risk in the Seguimiento Universidad de Navarra (SUN) cohort. Eur J Nutr. 2022;61(7):3517-30.
244. Vázquez-Ruiz Z, Toledo E, Vitelli-Storelli F, Goni L, de la OV, Bes-Rastrollo M, et al. Effect of Dietary Phenolic Compounds on Incidence of Cardiovascular Disease in the SUN Project; 10 Years of Follow-Up. Antioxidants (Basel). 2022;11(4).
245. Venø SK, Bork CS, Jakobsen MU, Lundbye-Christensen S, McLennan PL, Bach FW, et al. Marine n-3 Polyunsaturated Fatty Acids and the Risk of Ischemic Stroke. Stroke. 2019;50(2):274-82.

246. Veronese N, Cisternino AM, Shivappa N, Hebert JR, Notarnicola M, Reddavide R, et al. Dietary inflammatory index and mortality: a cohort longitudinal study in a Mediterranean area. J Hum Nutr Diet. 2020;33(1):138-46.
247. Vissers LET, Rijksen J, Boer JMA, Verschuren WMM, van der Schouw YT, Sluijs I. Fatty acids from dairy and meat and their association with risk of coronary heart disease. Eur J Nutr. 2019;58(7):2639-47.
248. Vogtschmidt YD, Soedamah-Muthu SS, Imamura F, Givens DI, Lovegrove JA. Replacement of Saturated Fatty Acids from Meat by Dairy Sources in Relation to Incident Cardiovascular Disease: The European Prospective Investigation into Cancer and Nutrition (EPIC)-Norfolk Study. Am J Clin Nutr. 2024;119(6):1495-503.
249. Voortman T, Chen Z, Girschik C, Kavousi M, Franco OH, Braun KVE. Associations between macronutrient intake and coronary heart disease (CHD): The Rotterdam Study. Clin Nutr. 2021;40(11):5494-9.
250. Wada K, Oba S, Nagata C. Rice-Based Diet and Cardiovascular Disease Mortality in Japan: From the Takayama Study. Nutrients. 2022;14(11).
251. Wan Y, Tabung FK, Lee DH, Fung TT, Willett WC, Giovannucci EL. Dietary Insulinemic Potential and Risk of Total and Cause-Specific Mortality in the Nurses' Health Study and the Health Professionals Follow-up Study. Diabetes Care. 2022;45(2):451-9.
252. Wang X, Yu C, Lv J, Li L, Hu Y, Liu K, et al. Consumption of soy products and cardiovascular mortality in people with and without cardiovascular disease: a prospective cohort study of 0.5 million individuals. Eur J Nutr. 2021;60(8):4429-38.
253. Wang X, Lv J, Yu C, Li L, Hu Y, Qin LQ, et al. Dietary Soy Consumption and Cardiovascular Mortality among Chinese People with Type 2 Diabetes. Nutrients. 2021;13(8).
254. Wang DD, Li Y, Nguyen XT, Song RJ, Ho YL, Hu FB, et al. Dietary Sodium and Potassium Intake and Risk of Non-Fatal Cardiovascular Diseases: The Million Veteran Program. Nutrients. 2022;14(5).
255. Wang K, Wang L, Liu L, Zhou P, Mo S, Luo S, et al. Longitudinal association of egg intake frequency with cardiovascular disease in Chinese adults. Nutr Metab Cardiovasc Dis. 2022;32(4):908-17.
256. Wang CR, Hu TY, Hao FB, Chen N, Peng Y, Wu JJ, et al. Type 2 Diabetes-Prevention Diet and All-Cause and Cause-Specific Mortality: A Prospective Study. Am J Epidemiol. 2022;191(3):472-86.
257. Wang L, Pan XF, Munro HM, Shrubsole MJ, Yu D. Consumption of ultra-processed foods and all-cause and cause-specific mortality in the Southern Community Cohort Study. Clin Nutr. 2023;42(10):1866-74.
258. Wang DD, Li Y, Nguyen XT, Song RJ, Ho YL, Hu FB, et al. Degree of adherence to plant-based diet and total and cause-specific mortality: prospective cohort study in the Million Veteran Program. Public Health Nutr. 2023;26(2):381-92.

259. Wang K, Chen Z, Shen M, Chen P, Xiao Y, Fang Z, et al. Dietary fruits and vegetables and risk of cardiovascular diseases in elderly Chinese. Eur J Public Health. 2023;33(6):1088-94.
260. Wang Q, Schmidt AF, Lennon LT, Papacosta O, Whincup PH, Wannamethee SG. Prospective associations between diet quality, dietary components, and risk of cardiometabolic multimorbidity in older British men. Eur J Nutr. 2023;62(7):2793-804.
261. Wang Y, Liu B, Hu Y, Sampson L, Manson JE, Rimm EB, et al. Phytosterol intake and risk of coronary artery disease: Results from 3 prospective cohort studies. Am J Clin Nutr. 2024;119(2):344-53.
262. Wang DD, Li Y, Nguyen XM, Ho YL, Hu FB, Willett WC, et al. Red Meat Intake and the Risk of Cardiovascular Diseases: A Prospective Cohort Study in the Million Veteran Program. J Nutr. 2024;154(3):886-95.
263. Ward RE, Cho K, Nguyen XT, Vassy JL, Ho YL, Quaden RM, et al. Omega-3 supplement use, fish intake, and risk of non-fatal coronary artery disease and ischemic stroke in the Million Veteran Program. Clin Nutr. 2020;39(2):574-9.
264. Weikart D, Lin D, Dhingra R, Al-Shaar L, Sturgeon K. Pre-Diagnosis Diet and Physical Activity and Risk of Cardiovascular Disease Mortality among Female Cancer Survivors. Cancers (Basel). 2022;14(13).
265. Weston LJ, Kim H, Talegawkar SA, Tucker KL, Correa A, Rebholz CM. Plant-based diets and incident cardiovascular disease and all-cause mortality in African Americans: A cohort study. PLoS Med. 2022;19(1):e1003863.
266. Wu WC, Huang M, Taveira TH, Roberts MB, Martin LW, Wellenius GA, et al. Relationship Between Dietary Magnesium Intake and Incident Heart Failure Among Older Women: The WHI. J Am Heart Assoc. 2020;9(7):e013570.
267. Würtz AML, Hansen MD, Tjønneland A, Rimm EB, Schmidt EB, Overvad K, et al. Replacement of potatoes with other vegetables and risk of myocardial infarction in the Danish Diet, Cancer and Health cohort. Br J Nutr. 2021;126(11):1709-16.
268. Xia X, Liu F, Yang X, Li J, Chen J, Liu X, et al. Associations of egg consumption with incident cardiovascular disease and all-cause mortality. Sci China Life Sci. 2020;63(9):1317-27.
269. Xia X, Liu F, Huang K, Chen S, Li J, Cao J, et al. Egg consumption and risk of coronary artery disease, potential amplification by high genetic susceptibility: a prospective cohort study. Am J Clin Nutr. 2023;118(4):773-81.
270. Xu Z, Steffen LM, Selvin E, Rebholz CM. Diet quality, change in diet quality and risk of incident CVD and diabetes. Public Health Nutr. 2020;23(2):329-38.
271. Xue T, Wen J, Wan Q, Qin G, Yan L, Wang G, et al. Association of soy food with cardiovascular outcomes and all-cause mortality in a Chinese population: a nationwide prospective cohort study. Eur J Nutr. 2022;61(3):1609-20.

272. Yamakawa M, Wada K, Goto Y, Mizuta F, Koda S, Uji T, et al. Associations between coffee consumption and all-cause and cause-specific mortality in a Japanese city: the Takayama study. Public Health Nutr. 2019;22(14):2561-8.
273. Yamakawa M, Wada K, Koda S, Uji T, Nakashima Y, Onuma S, et al. Associations of total nut and peanut intakes with all-cause and cause-specific mortality in a Japanese community: the Takayama study. Br J Nutr. 2022;127(9):1378-85.
274. Yang JJ, Lipworth LP, Shu XO, Blot WJ, Xiang YB, Steinwandel MD, et al. Associations of choline-related nutrients with cardiometabolic and all-cause mortality: results from 3 prospective cohort studies of blacks, whites, and Chinese. Am J Clin Nutr. 2020;111(3):644-56.
275. Yang Y, Dong JY, Cui R, Muraki I, Yamagishi K, Sawada N, et al. Consumption of flavonoid-rich fruits and risk of CHD: a prospective cohort study. Br J Nutr. 2020;124(9):952-9.
276. Yang J, Du H, Guo Y, Bian Z, Yu C, Chen Y, et al. Coarse Grain Consumption and Risk of Cardiometabolic Diseases: A Prospective Cohort Study of Chinese Adults. J Nutr. 2022;152(6):1476-86.
277. Yao X, Xu X, Wang S, Xia D. Associations of Dietary Fat Intake With Mortality From All Causes, Cardiovascular Disease, and Cancer: A Prospective Study. Front Nutr. 2021;8:701430.
278. Yazdanpanah MH, Sharafkhah M, Poustchi H, Etemadi A, Sheikh M, Kamangar F, et al. Mineral Intake and Cardiovascular Disease, Cancer, and All-Cause Mortality: Findings from the Golestan Cohort Study. Nutrients. 2024;16(3).
279. Yeung SSY, Zhu ZLY, Chan RSM, Kwok T, Woo J. Prospective Analysis of Fruit and Vegetable Variety on Health Outcomes in Community-Dwelling Chinese Older Adults. J Nutr Health Aging. 2021;25(6):735-41.
280. Yoshizaki T, Ishihara J, Kotemori A, Yamamoto J, Kokubo Y, Saito I, et al. Association of Vegetable, Fruit, and Okinawan Vegetable Consumption With Incident Stroke and Coronary Heart Disease. J Epidemiol. 2020;30(1):37-45.
281. Yoshizaki T, Ishihara J, Kotemori A, Kokubo Y, Saito I, Yatsuya H, et al. Association between irregular daily routine and risk of incident stroke and coronary heart disease in a large Japanese population. Scientific Reports. 2022;12(1):15750.
282. Yuan S, Damrauer SM, Håkansson N, Åkesson A, Larsson SC. A Prospective Evaluation of Modifiable Lifestyle Factors in Relation to Peripheral Artery Disease Risk. Eur J Vasc Endovasc Surg. 2022;64(1):83-91.
283. Zhang Y, Zhuang P, Wu F, He W, Mao L, Jia W, et al. Cooking oil/fat consumption and deaths from cardiometabolic diseases and other causes: prospective analysis of 521,120 individuals. BMC Med. 2021;19(1):92.
284. Zhang S, Li H, Engström G, Niu K, Qi L, Borné Y, et al. Milk intake, lactase persistence genotype, plasma proteins and risks of cardiovascular events in the Swedish general population. Eur J Epidemiol. 2023;38(2):211-24.

285. Zhang H, Yang J, Gu R, Yang J, Dong X, Ren Z, et al. Healthy diet habits attenuate the association of poor sleep quality with nonfatal ischemic stroke: A prospective rural cohort. Nutrition. 2024;126:112485.
286. Zhang Y, Tabung FK, Smith-Warner SA, Giovannucci E. High-quality fruit and vegetable characterized by cardiometabolic biomarkers and its relation to major chronic disease risk: results from 3 prospective United States cohort studies. Am J Clin Nutr. 2024;120(2):369-79.
287. Zhao Y, Naumova EN, Bobb JF, Claus Henn B, Singh GM. Joint Associations of Multiple Dietary Components With Cardiovascular Disease Risk: A Machine-Learning Approach. Am J Epidemiol. 2021;190(7):1353-65.
288. Zhao Y, Li D, Huang T. Associations of dietary flavonoids and subclasses with total and cardiovascular mortality among 369,827 older people: The NIH-AARP Diet and Health Study. Atherosclerosis. 2023;365:1-8.
289. Zhao B, Gan L, Graubard BI, Männistö S, Fang F, Weinstein SJ, et al. Plant and Animal Fat Intake and Overall and Cardiovascular Disease Mortality. JAMA Intern Med. 2024;184(10):1234-45.
290. Zheng J, Tabung FK, Zhang J, Caan B, Hebert JR, Kroenke CH, et al. Association between dietary inflammatory potential and mortality after cancer diagnosis in the Women's Health Initiative. Br J Cancer. 2023;128(4):606-17.
291. Zhong VW, Van Horn L, Greenland P, Carnethon MR, Ning H, Wilkins JT, et al. Associations of Processed Meat, Unprocessed Red Meat, Poultry, or Fish Intake With Incident Cardiovascular Disease and All-Cause Mortality. JAMA Intern Med. 2020;180(4):503-12.
292. Zhong GC, Gu HT, Peng Y, Wang K, Wu YQ, Hu TY, et al. Association of ultra-processed food consumption with cardiovascular mortality in the US population: long-term results from a large prospective multicenter study. Int J Behav Nutr Phys Act. 2021;18(1):21.
293. Zhong GC, Hu TY, Yang PF, Peng Y, Wu JJ, Sun WP, et al. Chocolate consumption and all-cause and cause-specific mortality in a US population: a post hoc analysis of the PLCO cancer screening trial. Aging (Albany NY). 2021;13(14):18564-85.
294. Zhong VW, Ning H, Van Horn L, Carnethon MR, Wilkins JT, Lloyd-Jones DM, et al. Diet Quality and Long-Term Absolute Risks for Incident Cardiovascular Disease and Mortality. Am J Med. 2021;134(4):490-8.e24.
295. Zhong VW, Allen NB, Greenland P, Carnethon MR, Ning H, Wilkins JT, et al. Protein foods from animal sources, incident cardiovascular disease and all-cause mortality: a substitution analysis. Int J Epidemiol. 2021;50(1):223-33.
296. Zhuang P, Zhang Y, He W, Chen X, Chen J, He L, et al. Dietary Fats in Relation to Total and Cause-Specific Mortality in a Prospective Cohort of 521 120 Individuals With 16 Years of Follow-Up. Circ Res. 2019;124(5):757-68.

297. Zhuang P, Wu F, Mao L, Zhu F, Zhang Y, Chen X, et al. Egg and cholesterol consumption and mortality from cardiovascular and different causes in the United States: A population-based cohort study. PLoS Med. 2021;18(2):e1003508.
298. Zhuang P, Liu X, Li Y, Ao Y, Wu Y, Ye H, et al. Dairy consumption and incident cardiovascular disease: a global analysis. medRxiv. 2023:2023.11.14.23298545.
